# Reduced IGF-1 signaling fails to limit Alzheimer’s disease progression in a novel rat model of *IGF-1R* haploinsufficiency

**DOI:** 10.1038/s41598-025-31601-1

**Published:** 2025-12-16

**Authors:** Sushma Narayan, Kai Mao, Alberto R. Williams-Medina, Todd Richmann, Maya Gal, Matthew Engel, Yongwei Zhang, Sarah Graff, Simone Sidoli, Nir Barzilai, Derek M. Huffman

**Affiliations:** 1 Department of Molecular Pharmacology, Albert Einstein College of Medicine, Bronx, NY 10461 USA; 2 Department of Medicine, Albert Einstein College of Medicine, Bronx, NY 10461 USA; 3 Department of Biochemistry, Albert Einstein College of Medicine, Bronx, NY 10461 USA; 4 Department of Genetics, Albert Einstein College of Medicine, Bronx, NY 10461 USA; 5 Institute for Aging Research, Albert Einstein College of Medicine, Bronx, NY 10461 USA; 6 Gene Targeting Core Facility, Albert Einstein College of Medicine, Bronx, NY 10461 USA; 7 Institute for Aging Research, Albert Einstein College of Medicine, 1300 Morris Park Ave, Golding Building Room 201, Bronx, NY 10461 USA

**Keywords:** Alzheimer’s disease, IGF-1, IGF-1R, Insulin resistance, Rat, Neuroscience, Physiology, Endocrinology, Medical research

## Abstract

The growth hormone/insulin-like growth factor-1 (GH/IGF-1) signaling pathway has been strongly implicated in the aging process. Lifespan is profoundly increased in both male and female dwarf mice, while low IGF-1 signaling per se leads to more modest improvements in female lifespan. However, as opposed to the consistency offered by studies in dwarf mice, nuances in the relationship of this axis with health and disease have also been observed, including in rat models of low GH/IGF-1 signaling. This complexity further extends to cognitive decline and Alzheimer’s disease (AD), where this relationship has proven to be nuanced. To help address these gap, we have generated a new rat model of *Igf1r*^+*/−*^ haploinsufficiency, to assess effects on metabolic health and AD. Similar to mice, we find that constitutively reduced IGF-1R levels leads to ~ 15% reduced adult body size in male and female rats, while not impairing insulin sensitivity. However, when crossed with TgF344-AD rats, lowering IGF-1 signaling per se failed to confer protection against AD-related pathology, including amyloid burden, phosphorylated tau or neuroinflammation in male and female TgF344-AD rats, and even appeared to exacerbate facets of disease in females, including an increase in cortical small amyloid plaques. Moreover, a unique hippocampal proteomic signature emerges in female Het/AD rats, including lower levels of proteins involved in redox balance. Overall, these data suggest a nuanced relationship of IGF-1R tone and AD exists and that better defining a more precise role of growth factor signaling in CNS health and disease throughout the life course is warranted.

**Supplementary Information:**

The online version contains supplementary material available at 10.1038/s41598-025-31601-1.

## Introduction

The growth hormone/insulin-like growth factor-1 (GH/IGF-1) signaling pathway is a conserved regulator of the aging process across nature, including, *C. elegans, Drosophila*, mice and humans^1–4^. In mammals, combined reductions in GH/IGF-1 action are associated with improvements in metabolic health and lifespan. For example, long-lived dwarf mice, which exhibit profound GH and IGF-1 deficiency, display enhanced insulin sensitivity and robust lifespan extension in both sexes^5,6^. Partial reductions in IGF-1 receptor (IGF-1R) signaling also promote longevity, though this effect is more modest than in dwarf models, and appears to be sex-specific, with benefits largely restricted to females^7–11^. Furthermore, heterozygosity for *Igf1r* is reported to promote insulin resistance in mice, though effects appear to be most pronounced in males^7,8,12^.

However, not all data support a pro-longevity effect of reduced GH/IGF-1 signaling. For example, in the Lewis dwarf (*dw/dw*) rat, which lacks GH and has low IGF-1 throughout life, lifespan is not extended unless GH is replaced during development^13^. In another rat model, overexpression of GH antisense led to a dose-dependent effect such that partial GH suppression slightly increased lifespan, while more complete suppression reduced longevity^14^. Importantly, these findings highlight the importance of dosage, timing and species differences in determining the role of GH/IGF-1 signaling on mammalian aging. Yet, the importance of IGF-1R signaling per se on health and aging beyond the mouse is mainly limited to human trials involving IGF-1R mAb treatment in cancer patients, where the goal is to substantially block IGF-1Rs, which can result in hyperglycemia and elevated hemoglobin A1c levels in some patients^15^.

Beyond its role in metabolism and longevity, the GH/IGF-1 signaling pathway has also been extensively investigated in brain aging and Alzheimer’s disease (AD), though definitive links remain inconclusive^16^. Long-lived dwarf models with combined reductions in GH and IGF-1 signaling show preserved cognitive function with age and attenuated AD-related pathology in the context of amyloidosis^17,18^. Likewise, whole body or neuron–specific reductions in IGF-1Rs also delay the accumulation of Aβ pathology, suggesting that dampened IGF-1 signaling may be protective against early AD-like changes^19–22^. However, in human AD brains, impaired responsiveness to IGF-1 and insulin has been observed^23^, although whether this purported resistance was a cause or consequence of disease is unclear. Human epidemiological studies linking IGF-1 to cognitive function are equally inconsistent^16^. As with survival outcomes, the impact of IGF-1 on cognition may be sex-dependent. For example, low serum IGF-1 correlates with better cognitive performance in elderly women, but not in men^24,25^. Given that AD disproportionately affects women, and drugs targeting the GH/IGF-1R pathway already exist, a better understanding of IGF-1 signaling on brain aging and AD, and potential sex-specific effects are needed, as it could hold important therapeutic implications.

While mouse models comprise a vast and important role in GH/IGF-1 research, rats may offer greater translational relevance to humans^26^. Compared to mice, rats exhibit more human-like physiology, such as insulin-stimulated glucose uptake^26^, possess larger brains, and demonstrate more complex social behaviors. However, there is a lack of genetically tractable rat models to directly manipulate IGF-1R signaling for studies on metabolic health and disease. To address this gap, we generated a novel rat model of *Igf1r* haploinsufficiency, resulting in > 50% reduction in IGF-1R levels across tissues. Consistent with comparable mouse models, *Igf1r* heterozygous rats exhibited a smaller body size and elevated IGF-1 levels in older females, without evidence of impaired glucose metabolism into adulthood. When *Igf1r* heterozygous rats were crossed with the TgF344-AD rat model of cerebral amyloidosis, low IGF-1R signaling did not attenuate Aβ pathology in either sex, but instead appeared to exacerbate the abundance of small plaques in females. Collectively, these data suggest that the role of IGF-1 signaling in rats on metabolic health and AD can be somewhat divergent from lessons in mouse models, and emphasize the importance of complementary species, such as the rat, in refining translational insights from preclinical models.

## Methods

### Generation of *Igf1r* mutant rat models

**Fig. 1:**
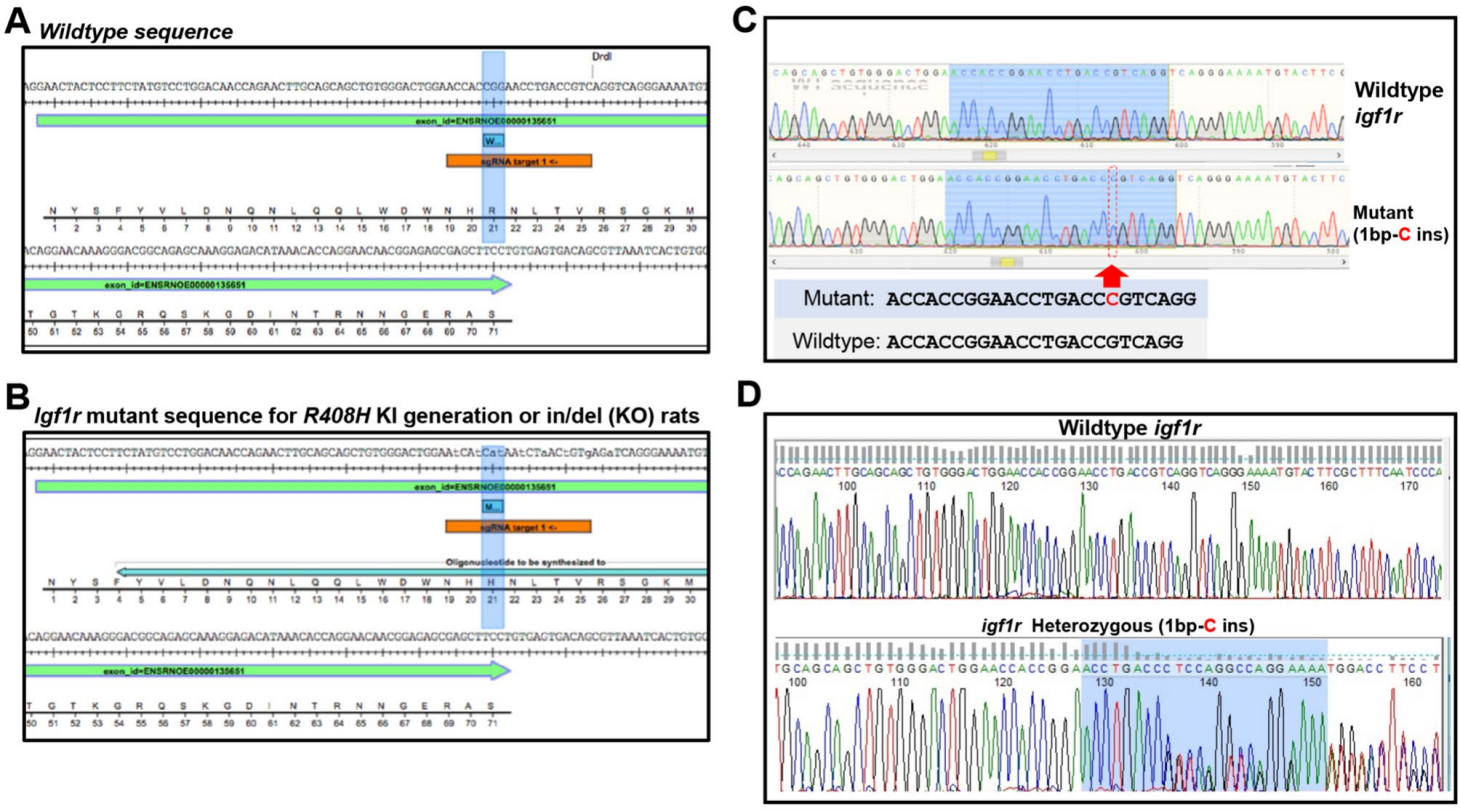
Strategy for generating and identifying novel *igf1r* heterozygous rats. (**A**,**B**) Mutant rats were derived by microinjecting fertilized eggs obtained from the mating of Fischer 344(cdf) animals with a CRISPR/Cas9 insertion of 24 base pairs to introduce the nonsynonomous mutation and 8 silent mutations, to remove *DdrI* restriction sites around the *R408* locus. This strategy was developed and executed at the University of Michigan Transgenic Core Facility. (**C**) Deep sanger sequencing of recombinant clones from both wild-type and mutant rats confirmed that heterozygous knockout animals harbored an insertion of a single nucleotide 'C' at the CRISPR target site creating a base shift mutation in the *igf1r* gene. **(D)** Due to the subtle shift created in the *rat igf1r* sequence, sequencing was conducted, often in duplicate, to confirm presence or absence of the mutant *igf1r* allele in offspring from the WT x *igf1r* Het breeding strategy.

Novel IGF-1R rat models, including a whole-body constitutive *Igf1r* knock-in (*R408H*), which corresponds to the *R407H* mutation found in human centenarians^27,28^, as well as rats harboring *Igf1r* haploinsufficiency were generated via a CRISPR/Cas9 gene editing strategy at the University of Michigan Transgenic Animal Model Core Facility. In brief, Mutant rats were derived by microinjecting fertilized eggs obtained from the mating of Fischer 344(cdf) animals with a CRISPR/Cas9 insertion of 24 base pairs to introduce the nonsynonomous mutation and 8 silent mutations, to remove *DdrI* restriction sites around the *R408* locus (Fig. 1A,B). A total of 10 candidate founder rats on an F344 background harboring either the oligonucleotide donor to introduce the *R408H* mutation or the formation of an in/del via nonhomologous end joining (a de facto* Igf1r* functional knockout) were identified by an inability of the *Drdl1* restriction enzyme to cut a PCR amplified fragment flanking the *R408* locus. As founders were presumed to be mosaic for either of the mutations, animals were subsequently mated with wild type F344 rats (Charles River Strain Code 002) and sequencing was performed on offspring to establish the colony of wild type and heterozygous animals (see Data Availability). Subsequent genotyping for all study rats was then performed and confirmed via sequencing.

To address potential for off-target effects of CRISPR, alternative cutting sites were predicted using genomic analyzing software Benchling (Einstein Gene Targeting Facility). Genomic DNA of one randomly selected WT, KO and *R408H* Knock-in rat were extracted using Qiagen DNeasy Blood and tissue Kits. An approximate 200 bp sequence flanking each of the top 12 off-target cutting sites on the prediction list were amplified and sequenced and compared with the reference genome (*Rattus norvegicus* strain mixed Rnor_6.0 release 106 using NCBI Blastn suite).

### Model validation and phenotyping study

Initial confirmation of reduced IGF1R levels was determined by Western Blot in multiple tissues of *Igf1r* heterozygous and wild type (WT) rats. Basic phenotypic characterization of male and female animals was further determined by assessing body composition via Echo MRI (ECHO MRS; Echo Medical Systems) and monitoring body weight up to 6 mo of age^29^. Plasma IGF-1 levels were measured using the Mouse/Rat IGF-1 Quantikine ELISA Kit (MG100; R&D Systems) Glucose and energy homeostasis was determined via the hyperinsulinemic-euglycemic clamp technique and indirect calorimetry, respectively. All animal experiments were approved by the Institutional Animal Care and Use Committee at the Albert Einstein College of Medicine in accordance with relevant guidelines and regulations with design and reporting adhering to the ARRIVE guidelines.

### *Igf1r* heterozygous and AD transgenic rats

To test the effect of low IGF-1 signaling in a rat model of AD-related pathology, we obtained F344-Tg(Prp-APP,Prp-PS1)19/Rrrc rats (abbreviated TgF344-AD)^30^ from the Rat Resource and Research Center (Univ of Missouri; Columbia MO, USA). This model is suggested to be an improvement over comparable mouse models of amyloidosis in that these rats manifest a full spectrum of age-dependent AD-related pathologies, preceded by robust levels of Aβ, followed by tauopathy, gliosis and substantial neuronal loss in the hippocampus and cortex, that is accompanied by cognitive impairment and brain atrophy. TgF344-AD rats were bred with *Igf1r* Heterozygous (*Igf1r*^+*/−*^; *Igf1r* Het) animals to generate both male and female groups as follows: (1) wild-type control (WT) (n = 23 total per group; 13 male, 10 female), (2) *Igf1r* Het (n = 22 total per group; 11 male, 11 female), (3) WT x TgF344-AD (n = 23 total per group; 11 male, 12 female), and (4) *Igf1r* Het x TgF344-AD (n = 24 total per group; 10 male, 14 female). As the TgF344-AD model has been previously shown to exhibit several forms of AD-related dysfunction by 15–16 mo age, including amyloidosis, gliosis, tauopathy, and cognitive deficits, we assess the cognitive phenotypes at 13–14 mo of age. Upon completion of functional assays, animals were euthanized at 15 mo of age by exsanguination under isoflurane anesthesia for collection of whole blood followed by a brief PBS transcardial perfusion for 3 min to remove residual blood prior to brain tissue collection and processing.

### Hyperinsulinemic-euglycemic clamps

Whole-body insulin sensitivity was determined via performing hyperinsulinemic-euglycemic clamp studies in male and female rats similar to previously described^31,32^. All studies were 240 min in duration and consisted of a 120 min basal period and a 120 min hyperinsulinemic clamp period. At t = 0 min, which is the beginning of the basal period, a primed-continuous infusion of [3-^3^H]-glucose (20uCi bolus, 0.2uCi/min maintenance; NEN Life Science Products, Boston, MA) was given into the jugular vein and maintained throughout the remainder of the study. The hyperinsulinemic-euglycemic clamp was then initiated at t = 120 min by peripheral administration of a primed-continuous infusion of regular insulin (3 mU kg^−1^ min^−1^), and somatostatin (1.5 µg kg^−1^ min^−1^) was also provided to suppress endogenous insulin secretion. A 25% glucose solution was given and periodically adjusted to clamp the plasma glucose concentration at ~ 140–145 mg/dL. Serum samples for determination of [3-^3^H]-glucose and [3-^3^H]-glucose water specific activities (SAs) were obtained at 10-min intervals during the basal and clamp periods. At the completion of the study, rats were euthanized by exsanguination under isoflurane anesthesia and tissues were then rapidly excised, weighed and flash frozen in liquid nitrogen, prior to storage at − 80 °C.

### Indirect calorimetry

Energy expenditure, substrate utilization and spontaneous activity were determined in metabolic cages to perform indirect calorimetry, based upon O_2_ consumption and CO_2_ production, using a Rat Oxymax System (Columbus Instruments, Columbus, OH) as described^31,33^. In brief, rats (*n* = 8 per group) were placed into individual cages at their standard temperature and photoperiod and allowed to acclimate for at least 72 h prior to the experiment and allowed ad libitum access to food and water. Beginning at 1600 h, data were collected over 24 h on the final day for determination of energy expenditure (EE), respiratory exchange ratio (RER) and activity levels.

### Dermal fibroblast isolation and culture

Rat tail tips (~ 0.5 cm) were collected, rinsed in 70% ethanol, minced, and digested in DMEM containing 2.5 mg/mL Liberase (Sigma) at 37 °C for 3–3.5 h. Digested tissue was filtered through a 0.2 µm cell strainer, and centrifuged at 1500 rpm for 15 min at room temperature. The cell pellet was resuspended in DMEM supplemented with 10% FBS and 1% Antibiotics, and plated in 35 mm culture dishes. Medium was changed after 24 h, and then every 2–3 days. Cells were passaged at ~ 70% confluence using trypsin–EDTA. For cryopreservation, cells were resuspended in freezing medium (DMEM + 10% FBS + 10% DMSO) at 5 × 10^5^ cells/mL, frozen at –80 °C overnight, and transferred to liquid nitrogen. Cells (passage 2–3) were challenged with IGF-1 (25 nM) and subsequently collected 10 min later for evaluation of signaling via Western blotting.

### Western blotting

For standard Western Blotting, 30–50 mg of target tissue was lysed in 500ul of RIPA buffer (120 mM NaCl, 50 mM Tris–HCl pH 7.4, 0.25% Deoxycholate, 5 mM EDTA, 1 mM Orthovanadate, 5 mM Na Pyrophosphate, 1% Triton X-100) with PMSF, NaF and a protease inhibitor cocktail (PIC) tablet (Roche). Extracted protein concentrations were read by using a BCA protein assay (Sigma, St. Louis, MO) with BSA as standards. A total of 20 µg of protein was denatured and reduced by lithium dodecyl sulfate (LDS, Invitrogen #2165457) and dithiothreitol (DTT, Invitrogen #2353153) at 70 °C and was separated on precast Criterion TGX stain-free gels (4–20%, Biorad #5678095). Samples were electrophoresed at 120 V constant for 90 min. Prior to transfer, stain-free images were captured on a Biorad Chemidoc MP Imaging System (Biorad, Hercules, CA) to confirm equal protein loading, and then they were wet transferred onto PVDF membranes in ice-cold transfer buffer at 100 V constant for 1 h. Equal transfer was confirmed by Ponceau-S (Sigma, 6626-79-5) staining. In order to allow for more efficient protein detection via Western Blot, membranes were cut at predetermined sizes for targets in which the molecular weight of the protein target is well established by us and others, by manually cutting into horizontal strips using a Biorad Kaleidoscope ladder as reference, with sufficient margins above and below the expected band size to capture the band(s) of interest. Original blots as acquired by the Chemidoc, can be referenced in Supplementary Files. Membranes were blocked in 5% milk or BSA in TBST and then incubated with appropriate primary antibodies from Cell Signaling Technologies (Danvers, MA) against IGF-1R (Cell Signaling; #9750), InsR (Cell Signaling; #3025), p-GSK3β (Cell Signaling; #5558), total GSK3β (Cell Signaling; #12456), p-Tau(Ser202/Thr205) (Thermo Fisher AT8 #MN1020), total Tau (Cell Signaling; #46687), amyloid-beta (Cell Signaling; #8243), pS6 (catalog #5364), total S6 (Cell Signaling; #2217), p-Erk (Cell Signaling; #9101), total Erk (Cell Signaling; #4695), p-MAP2 (Ser136) (Cell Signaling #4541), Total MAP2 (Cell Signaling; #8707) overnight at 4 °C overnight with gentle agitation. Following a 1 h incubation with HRP-linked anti-rabbit secondary antibody (Cell Signaling; #7074P2), Clarity Western ECL (Biorad, #1705062) substrate was applied to membranes to visualize bands using the Biorad Chemidoc MP Imaging System to first pixel saturation and densitometry performed using Image Lab (Biorad, Hercules, CA).

### Immunohistochemistry

Saline-perfused brains were hemisected along the sagittal midline and the right hemisphere was transferred to 4% paraformaldehyde (PFA) for post-fixation followed by cryoprotection via 30% sucrose, embedded in M-1 embedding matrix and stored at − 80 °C. Free-floating 30 µm sagittal sections were subjected to standard immunofluorescence (IF) or diaminobenzidine (DAB) staining techniques. Immunostaining was performed as previously described^34,35^. In brief, perfused brains were excised and one hemisphere was infiltrated with 4% paraformaldehyde (PFA) in 1X PBS overnight at 4 °C followed by immersion in 30% sucrose solution. Brains were preserved in M1 embedding matrix (Epredia, #1310) and stored at − 80 °C. Brains were cryosectioned coronally at 30um thickness with localized sectioning in the hippocampal region. Sections were stored free-floating in anti-freeze media (ethylene glycol, 1X PBS, 0.2 M PB, H2O) at − 20 °C until staining. Sections picked for staining were washed in 1X PBS and incubated in 3% H_2_O_2_ (Sigma; 772-84-1) prior to blocking in 10% NGS for 1 h followed by overnight primary incubation for the appropriate antibodies against amyloid-beta (Cell Signaling; #8243), GFAP (Cell Signaling; 80788), and MAP2 (Cell Signaling; #8707) at 4 °C with gentle agitation. Tissues were then incubated in secondary (Vector Laboratories; Ba-1000) for 1 h at RT in ABC Vectastain (Vector Labs; PK-6100) for 1 h before undergoing DAB incubation (ScyTek, ACK600). Tissues were mounted on slides and left to dry overnight before dehydration in 70% EtOH, 95% EtOH, and 100% EtOH, followed by clearing in 100% Xylene. Finally, slides were coverslipped with permount (Fisher; SP15-100).

Slides were visualized on the P1500 Flash Slide Scanner on bright field setting. Square annotations (2000um perimeter) were taken from posterior parietal association areas and primary somatosensory areas for cortical regions and CA1 and CA3 for hippocampal regions. Amyloid-beta positivity was included in the analysis above 20 µm diameter. To assess distribution, only plaques that were ≥ 20 µm were included and then categorized by size as follows: 20–39.9 µm, 40–59.9 µm, 60–79.9 µm, and 80+  µm bins. Plaque burden (%) was determined as: [(Count × Mean Plaque Size)/250,000]*100. MAP2 was calculated as a dark-brown intensity analysis (QuantCenter; ratio of strong brown pixels to total pixels reported as percentage).

### RNA isolation and qPCR

RNA was isolated using approximately 30 mg of target issue via mechanical homogenization in TRIzol, followed by chloroform extraction as described^34,35^. Pellets were resuspended in RNAase-free H_2_O and stored at − 80 °C. Genomic DNA was removed using DNAse and cDNA was synthesized using an iScript Synthesis Script (Biorad, catalog #). The SsoAdvanced SYBR Green mix (Biorad, catalog #) was used to run qPCR on a qRT-PCR machine (Biorad CFX384) for *gfap*, *Il6*, *trem2*, *iba1* and *map2*.

### Proteomics

Proteomics was performed by the Einstein Proteomics Core, similar as described^36^. In brief, an average of 200 µg of total protein from rat hippocampus (n = 4–6 per group) suspended in 25 µL of RIPA lysis buffer was loaded onto a 96-well plate. An equal volume of 50 mM ammonium bicarbonate containing 5% SDS was added to each well for a starting volume of 50 µL. Protein lysates were reduced and alkylated with DTT and iodoacetamide, respectively, and proteins precipitated by adding 12% phosphoric acid to a final concentration of 1.2% phosphoric acid per well. S-trap binding buffer (90% MetOH/10 mM ammonium bicarbonate) was added to each well at 6× the remaining volume per well and loaded onto an S-trap 96-well plate mounted on a 2 mL Eppendorf 96-well plate. A series of 3 washes with S-trap binding buffer followed and flowthroughs were discarded. Captured proteins (plate columns) were cleaved via overnight trypsin (8 ng/µL, 125 µL per well) digestion at 37 °C, and peptides were eluted with 0.1% trifluoroacetic acid and 60% acetonitrile/0.1% trifluoroacetic acid and stored at − 20 °C. Peptide eluates were then dried via speed vacuum, desalted, and processed via tandem liquid chromatography coupled with mass spectrometry (LC/MS).

### LC–MS/MS acquisition and analysis

Samples were resuspended in 10 µl of 0.1% TFA and loaded onto a Dionex RSLC Ultimate 300 (Thermo Scientific), coupled online with an Orbitrap Fusion Lumos (Thermo Scientific). Chromatographic separation was performed with a two-column system, consisting of a C-18 trap cartridge (300 µm ID, 5 mm length) and a picofrit analytical column (75 µm ID, 25 cm length) packed in-house with reversed-phase Repro-Sil Pur C18-AQ 3 µm resin. Peptides were separated using a 90 min gradient from 4 to 30% buffer B (buffer A: 0.1% formic acid, buffer B: 80% acetonitrile + 0.1% formic acid) at a flow rate of 300 nl/min. The mass spectrometer was set to acquire spectra in a data-dependent acquisition (DDA) mode. Briefly, the full MS scan was set to 300–1200 m/z in the orbitrap with a resolution of 120,000 (at 200 m/z) and an AGC target of 5 × 10e5. MS/MS was performed in the ion trap using the top speed mode (2 secs), an AGC target of 1 × 10e4 and an HCD collision energy of 35.

Proteome raw files were searched using Proteome Discoverer software (v2.5, Thermo Scientific) using SEQUEST search engine and the SwissProt database. The search for total proteome included variable modification of N-terminal acetylation, and fixed modification of carbamidomethyl cysteine. Trypsin was specified as the digestive enzyme with up to 2 missed cleavages allowed. Mass tolerance was set to 10 pm for precursor ions and 0.2 Da for product ions. Peptide and protein false discovery rate was set to 1%. Raw data files are available online.

### Proteomic data processing and analysis

Raw proteomic intensity values were imported into Python (3.9.23) using pandas and values were log₂ transformed to stabilize variance, then proteins detected in ≤ 10% of samples were removed to reduce sparsely detected proteins. Log₂ values were mean normalized per sample to center each sample distribution. Remaining missing values were imputed by drawing random values from a left-shifted Gaussian distribution (mean shifted using − 2.5SD), as implemented via NumPy’s (1.26.4) uniform sampler.

### Quality control

Data quality and sample relationships were assessed by computing pairwise Pearson correlations across all samples, followed by hierarchical clustering using Ward’s linkage on Euclidean distances via fastcluster (1.2.6). A clustered heatmap of the correlation matrix was rendered with Seaborn’s (0.13.2) clustermap function. Principal Component Analysis (PCA) was performed on StandardScaler scaled data via scikit-learn (1.6.1) to visualize sample structure and check for outliers within the dataset.

### Differential expression

For each comparison between conditions, mean intensities were computed per group and fold-changes defined as log₂ difference “experimental/control.” Variance equality was tested by F-test; two-sample t-tests (Welch when unequal variances) were used to derive p-values, which were transformed to –log₁₀ scale for visualization. Both uncorrected (α = 0.05) and Benjamini–Hochberg (BH) thresholds were calculated. Differentially expressed proteins were plotted in a volcano plot using matplotlib (3.9.2), and top 25 hits were annotated by score (-log(p-value) · log_2_ fold change).

### Gene set enrichment analysis (GSEA)

Proteins were ranked by signed test statistic (score, as previously described) and prepared for pre-ranked GSEA using gseapy (0.9.5) against MSigDB’s GO *Molecular Function, Biological Process,* and *Cellular Component* collections’ 2025 release. Enrichment results (normalized enrichment score (nes), p-values, gene ratios) were read into pandas, and the top 20 or 15 positive and negative terms were visualized as a dot plot including nes, gene ratio, and statistical significance. All plots and statistical computations were performed using the following standard Python libraries to ensure reproducibility and statistical correction for multiple comparisons: NumPy, SciPy (1.13.1), scikit-learn, Seaborn, and Matplotlib.

### Behavioral assays

Cognitive and behavioral assessments were conducted between 13 and 14 mo of age by an experimenter who was blinded to the experimental groups. Open field assessment was used to determine voluntary locomotor activity and anxiety-like behavior. In brief, animals were placed in the corner of an evenly lit square arena (80 cm × 80 cm, 41 cm height) without visual cues (Maze Engineers; Skokie, IL), and average velocity, track length, activity level, and time spent in the central zone were recorded for 15 min using an aerial camera and companion software (Bioseb, Inc).

For assessment of hippocampal-dependent spatial learning, a Barnes Maze Platform (TSE Systems; Chesterfield, MO), measuring 1220 mm in diameter and consisting of 20 equally spaced holes around the perimeter. For the assessment, animals were gently placed in the center of the brightly lit platform and evaluated for their ability to find the one true escape leading to a dark and enclosed refuge compartment as the motivating factor. During habituation (Day 1), animals were placed in the dark escape refuge for 2 min before being gently removed and placed in the center of the platform and allowed to explore for 5 min. If the animal was able to locate the escape cage in under 5 min, the latency was recorded, otherwise, the animal was guided to the escape hole and compartment and removed after an additional 15 s. The assessment was then repeated on days 2, 3, 4 (memory) and 7 (retention).

Finally, an Elevated Plus Maze for rats (Maze Engineers) was used for assessment of anxiety-like behavior, exploratory behavior, risk assessment behavior, and locomotion. Animals were placed on an elevated, cross-shaped apparatus with both exposed (or open, brightly lit) arms and enclosed (or closed, dimly lit) arms. Animals were allowed to explore the four arms for 5 min. The amount of time the animal spent in the open arms was then recorded.

### Statistics

Cross-sectional data were analyzed using either independent sample t-tests or two-way ANOVA (genotype x treatment), while longitudinal measures were assessed by two-way ANOVA with repeated measures on time. Male and female data were analyzed separately. Planned contrasts were performed as appropriate with Tukey adjustment. Normality of distribution was routinely assessed via the Shipiro-Wilk procedure and log transformed when necessary to ensure equal distribution. All analyses were performed using Prism software. Values are reported as means ± standard error (SE). A p ≤ 0.05 was considered statistically significant.

## Results

### *Igf1r* haploinsufficient rats have reduced IGF1R protein levels in several tissues

**Fig. 2:**
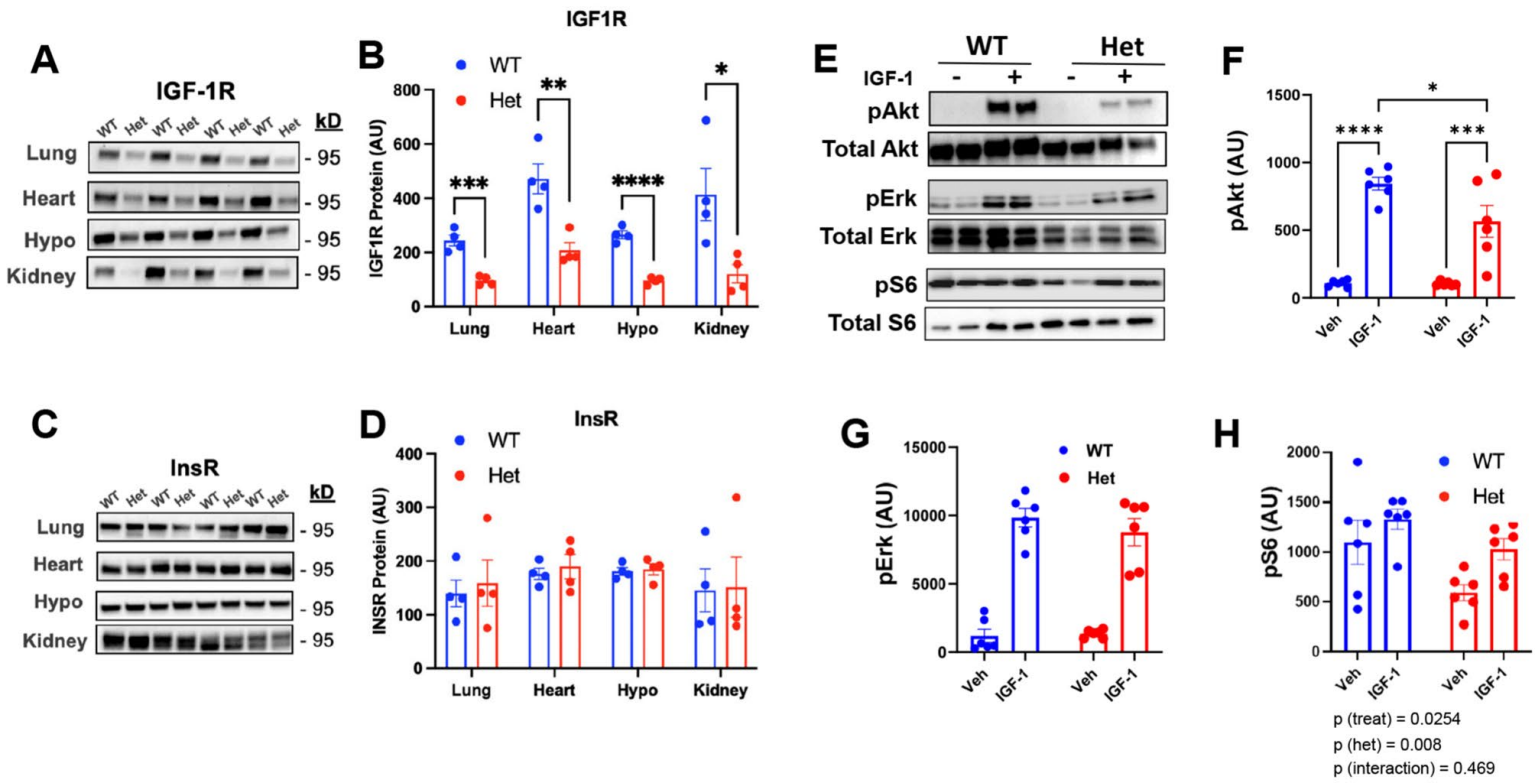
Characterization, and validation of *Igf1r* heterozygous rats. (**A**,**B**) We first confirmed that the 1 bp-C insert resulted in a functional *Igf1r* knockout on one allele by performing western blotting of IGF-1R in several tissues. These studies revealed that IGF-1R expression was reduced by > 50% in lung, heart, hypothalamus and kidney of male rats (n = 4 per group). (**D**) As a positive control, InsR was also measured in the same samples and confirmed to not be altered in animals. (**C**–**F**) To further confirm that *igf1r* heterozygosity results in attenuated signaling, we treated dermal fibroblasts isolated from young control and Het rats and administered either Vehicle or 25 nM IGF-1 for 10 min. As can be seen, *Igf1r* Het fibroblasts demonstrate an attenuation in activation of pAkt, but not pErk and pS6 under IGF-1 stimulated conditions (**C**–**F**; p < 0.05). Statistics were performed either via independent samples t-test or two-way ANOVA, followed by posthoc tests with Tukey adjustment. Bar graphs represent mean ± S.E.M. *p < 0.05, **p < 0.01, ***p < 0.001, ****p < 0.0001.

A CRISPR/Cas9 and guide RNA strategy targeting the *R408H* position with exclusion of a *DrdI* restriction site within the wildtype *Igf1r* gene was devised in order to generate mutant *IGF-1R* rat models, including an *Igf1r* haploinsufficient rat (Fig. 1A,B). In order to confirm *Igf1r* heterozygosity, we first performed Sanger sequencing of recombinant clones from both wild-type and mutant rats. Indeed, it was confirmed that heterozygous knockout animals harbored an insertion of a single nucleotide 'C' at the CRISPR target site creating a base shift mutation in the *Igf1r* gene (Fig. 1C,D; see Data Availability). Furthermore, while our on-target site was confirmed to be modified, assessment of 12 random off-target genomic sites among the genome with similar restriction sites did not detect any irregularities, suggesting no detectable off-target effects of our CRISPR strategy (Table. S1). We next harvested several tissues from WT and mutant animals at 3–4 mo of age and performed western blots for both IGF1R and Insulin receptor (InsR) levels. As shown in Fig. 2A,B, IGF-1R levels were indeed reduced by > 50% in several tissues, including lung, heart, hypothalamus and kidney (p < 0.05), but no differences were observed in InsR (Fig. 2C,D). To further confirm an attenuation in IGF-1R signaling, isolated dermal fibroblasts from WT and *Igf1r* Het animals, respectively, were challenged with 25 nM IGF-1, which revealed an attenuation in activation of pAkt (Fig. 2E,F; p < 0.05), but not pErk and pS6 activation under stimulated conditions in Het animals (Fig. 2E,G,H).

### Metabolic characteristics of *Igf1r* heterozygous rats

**Fig. 3:**
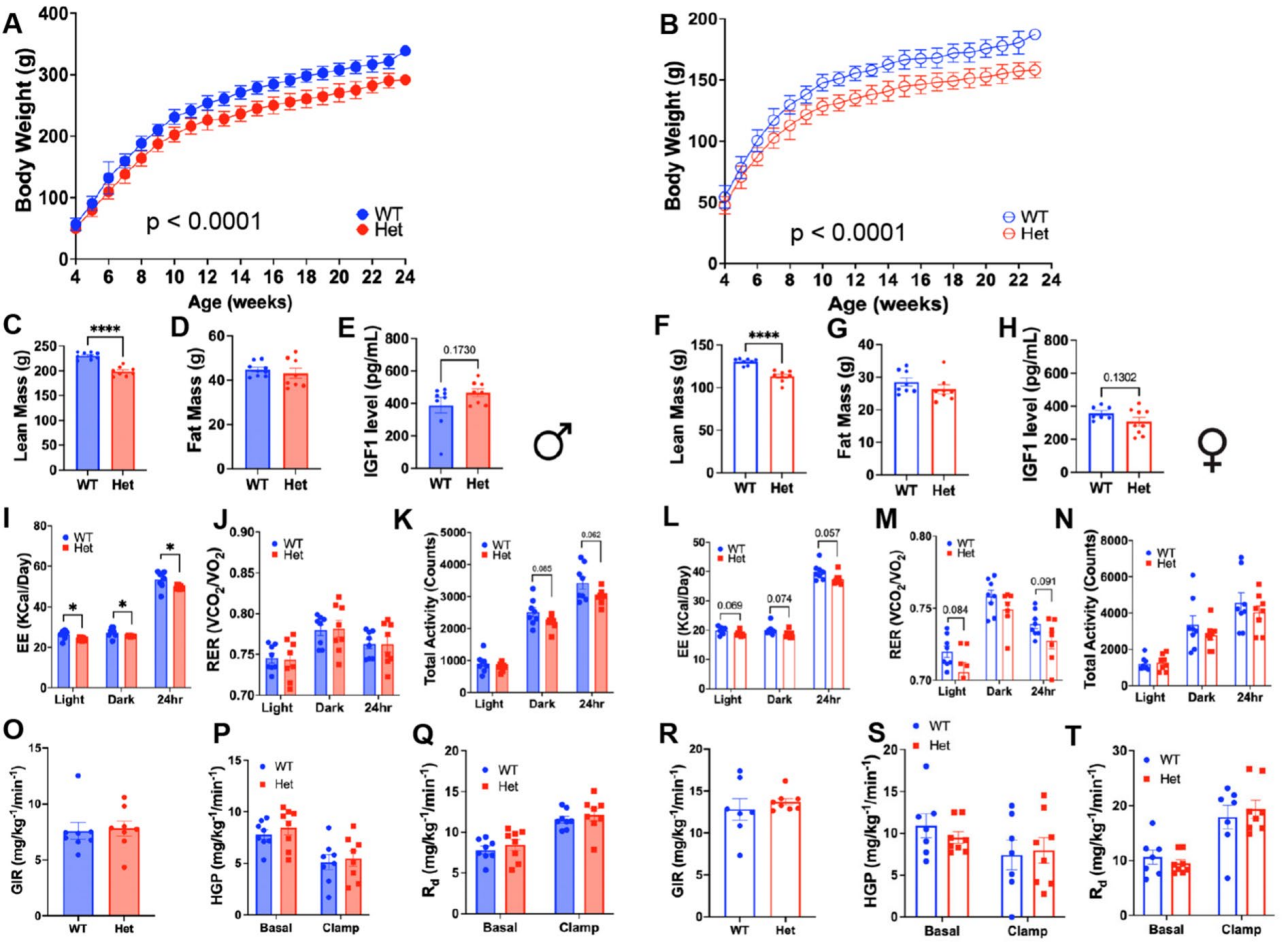
Metabolic characteristics of *IGF-1R* heterozygous male and female rats. (**A**,**B**) *Igf1r* Het animals were similar in size during development (4 wks of age), but displayed a reduction in body weight reaching adulthood by approximately 15% in both males (WT n = 14; *igf1r* Het n = 13) and females (WT n = 11; *igf1r* Het n = 12). (**C**–**E**) In males and (**F**–**H**) females, body composition measures by qMR confirmed that the reduction in body weight was largely attributed to a significant reduction in lean mass, but not fat mass, without significant changes in circulating IGF-1 levels. (**I**–**K**) Metabolic cage assessment was also performed in study animals. In males, (WT n = 8; *igf1r* Het n = 8) consistent with the reduction in body mass in males, energy expenditure (EE) was significantly reduced during the light and dark phase as well as over 24 h in *Igf1r* Hets (p < 0.05), but respiratory exchange ratio (RER) was not different between groups. (**L**–**N**) Similarly, females (WT n = 8; *igf1r* Het n = 8) tended to have lower total EE, while RER was numerically reduced in female HETs (p = 0.091), without difference in activity levels. (**O**–**T**) Insulin action was determined via hyperinsulinemic-euglycemic clamps. *igf1r* Hets had no detectable impairment in whole-body insulin action, including hepatic glucose production or glucose uptake in either males (WT n = 8; *igf1r* Het n = 8) or females (WT n = 7; *igf1r* Het n = 8). Body weight was assessed by Repeated Measures ANOVA while other assessments were performed via independent samples t-test. Lines and bars represent mean ± S.E.M. *p < 0.05, ****p < 0.0001.

We next performed phenotypic characterization of several metabolic traits in *Igf1r* Het male and female rats (Fig. 3). Consistent with comparable mouse models, while Het animals were similar in size during development, they were notably smaller than WT upon reaching adulthood, with body weights reduced by approximately 15% in both male (Fig. 3A) and female (Fig. 3B) mutant animals. Body composition measures by qMR confirmed that the reduction in body weight was largely attributed to a significant reduction in lean mass in males (Fig. 3C; p < 0.001) and females (Fig. 3F; p < 0.001), without changes in adiposity in males (Fig. 3D) or females (Fig. 3G). Interestingly, plasma IGF-1 levels were not significantly different in Het male (Fig. 3E) or female rats (Fig. 3H).

Consistent with the reduction in body mass in males, energy expenditure (EE) was significantly reduced during the light and dark phase as well as over 24 h in male *Igf1r* Hets (Fig. 3I; p < 0.05). There were no differences in substrate utilization in male rats (Fig. 3J), but *Igf1r* Het males tended to have lower activity over 24 h (Fig. 3K; p = 0.062). Similar to males, females tended to have lower total EE (Fig. 3L; p = 0.057) while RER was numerically, albeit non-significantly reduced in female *Igf1r* HETs (Fig. 3M; p = 0.091), and activity levels were not different (Fig. 3O). Moreover, when evaluating insulin action via hyperinsulinemic-euglycemic clamps, *Igf1r* Hets had no detectable impairment in whole-body insulin sensitivity, including hepatic glucose production or glucose uptake in either males (Fig. 3O–Q) or females (Fig. 3R–T).

### Reduced IGF-1 signaling fails to lower amyloid burden, but alters plaque size distribution in female rats

**Fig. 4:**
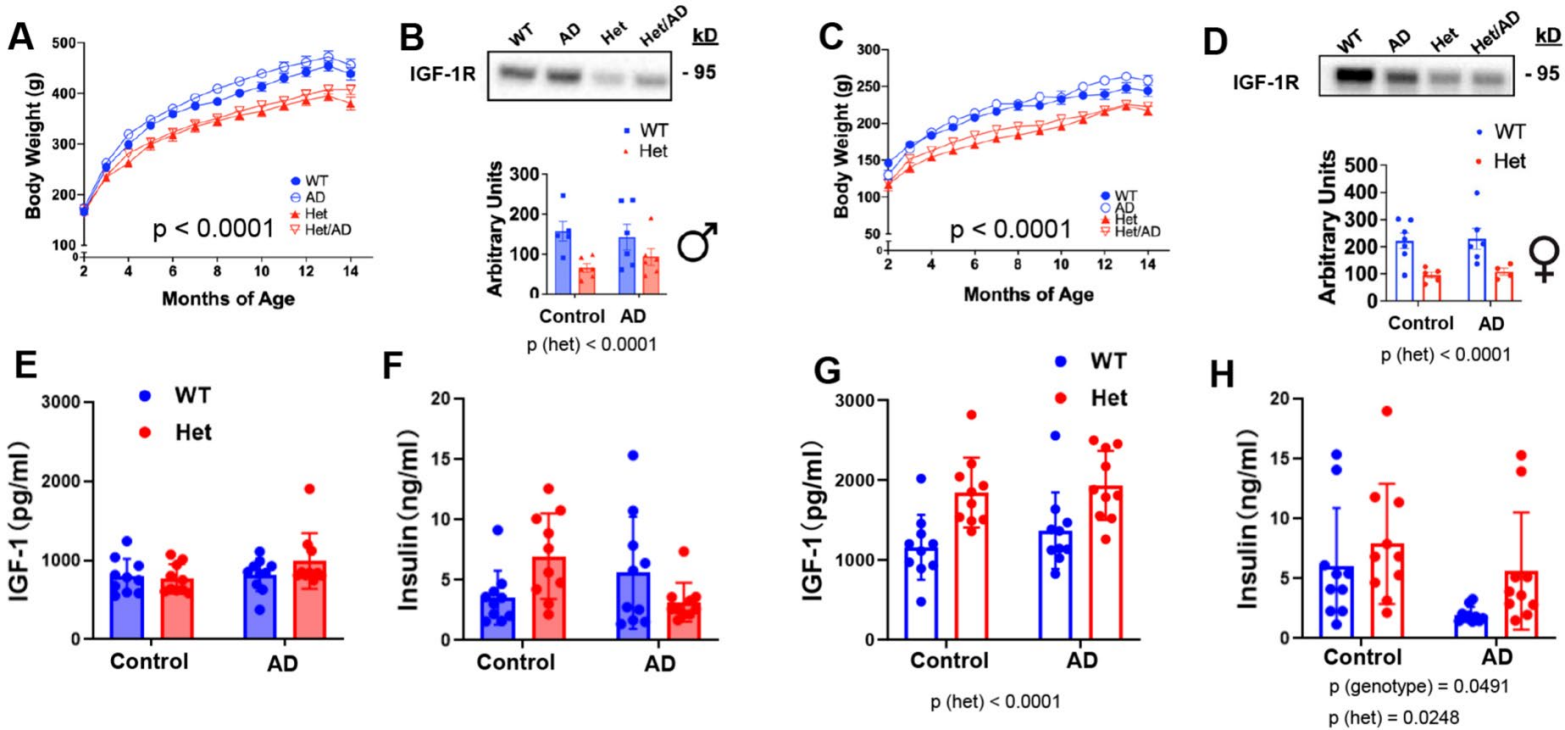
Effect of *Igf1r* haploinsufficiency on metabolic-related parameters in male and female Tg344-AD rats. We crossed *igf1r* Hets with TgF344-AD rats to generate four experimental groups, respectively. (**A**,**C**) In, *igf1r* Hets, there was a reduction in body weight for both males (WT n = 13; *IGF-1R* Het n = 11; AD n = 11; Het-AD n = 10) and females (WT n = 10; *igf1r* Het n = 11; AD n = 12; Het-AD n = 14), relative to non-AD controls. (**B**,**D**) As expected, IGF-1R levels in cortex were reduced in both control and TgF344-AD *Igf1r* Het animals of both sexes (p < 0.001). (**E**,**F**) Neither plasma IGF-1 or Insulin levels were significantly altered in male animals. (**E**,**F**) In Females, IGF-1 (**G**; p < 0.001) and insulin concentrations (**H**; p = 0.0248) were significantly elevated in female Hets, regardless of AD genotype status. Body weight was assessed by Repeated Measures ANOVA while other assessments were performed via two-way ANOVA followed by posthoc tests with Tukey adjustment.

Studies in mouse models have shown that reduced IGF-1 signaling via *Igf1r* haploinsufficiency reduces disease burden in models of amyloidosis. Thus, we next aimed to determine if lower IGF-1R signaling could phenocopy these effects in a comparable rat model of AD. To this end, we crossed *Igf1r* Hets with TgF344-AD rats to generate four male and female experimental groups, respectively. Consistent with our initial observations, *Igf1r* Heterozygosity reduced body weight in WT control and TgF344-AD rats of both male (Fig. 4A; p < 0.001) and female (Fig. 4C; p < 0.001) rats, along with lower IGF-1R levels in cortex in both sexes (Fig. 4B,D; p < 0.001), regardless of control or TgF344-AD genotype. In males, this corresponded to a lower GSK3β phosphorylation in cortex in Het rats (Fig. S1A) but no differences were observed in pS6 or pErk (Fig. S1B,C), nor were there any differences among females (Fig. S1D–F). However, while neither plasma IGF-1 or Insulin levels were significantly altered in male animals (Fig. 4E,F), IGF-1 (Fig. 4G; p < 0.001) and insulin concentrations (Fig. 4H; p = 0.0248) were significantly elevated in female Hets, regardless of AD genotype status.

**Fig. 5:**
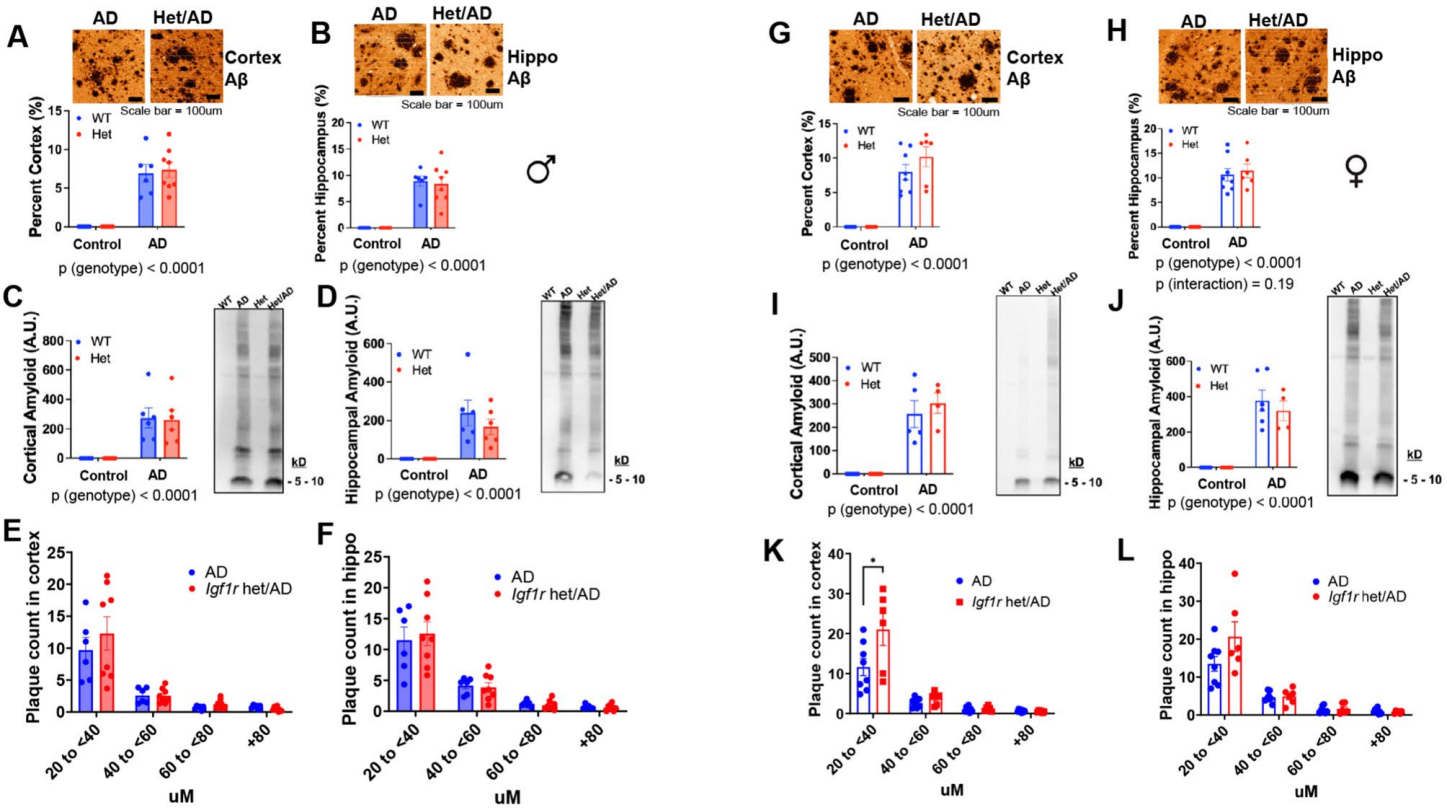
Effect of IGF-1 signaling on amyloid burden and remodeling in Tg344-AD rats. (**A**–**D**) We characterized effects on AD-related pathology in male rats using IHC and western blotting for low molecular weight oligomers (5-10kD), but no difference was observed in amyloid burden in either hippocampus or cortex. (**E**,**F**) We further assessed amyloid plaque distribution by size in cortex and hippocampus, but there were no differences observed between control AD and Het AD males. (**G**–**J**) Similar to males, no difference was observed in amyloid burden between control and *Igf1r* Het AD females. (**K**,**L**) However, when plaque distribution was assessed, small plaque counts (20 to < 40uM) were increased in *Igf1r* het/AD female cortex (**K**; p < 0.05), but not in hippocampus (**L**). Statistics were performed either via independent samples t-test or two-way ANOVA, followed by posthoc tests with Tukey adjustment. Bar graphs represent mean ± S.E.M. *p < 0.05.

We next characterized effects on AD-related pathology in rats using orthogonal assays. No difference was observed in total amyloid burden in hippocampus or cortex in males by either IHC (Fig. 5A,B) or Western blot (Fig. 5C,D) in male or female *Igf1r* Het AD (Fig. 5G–J). Likewise Total and pTau in male (Fig. S2A,B) and female (Fig. S2C,D) cortex and hippocampus, respectively, were not significantly different among groups. We further assessed Amyloid plaque distribution by size in cortex and hippocampus. While there were no differences observed between control AD and Het AD males (Fig. 5E,F), small plaque counts (20 to < 40uM) were increased in *Igf1r* Het/AD female cortex (Fig. 5K; p < 0.05), but not in hippocampus (Fig. 5L).

**Fig. 6.**
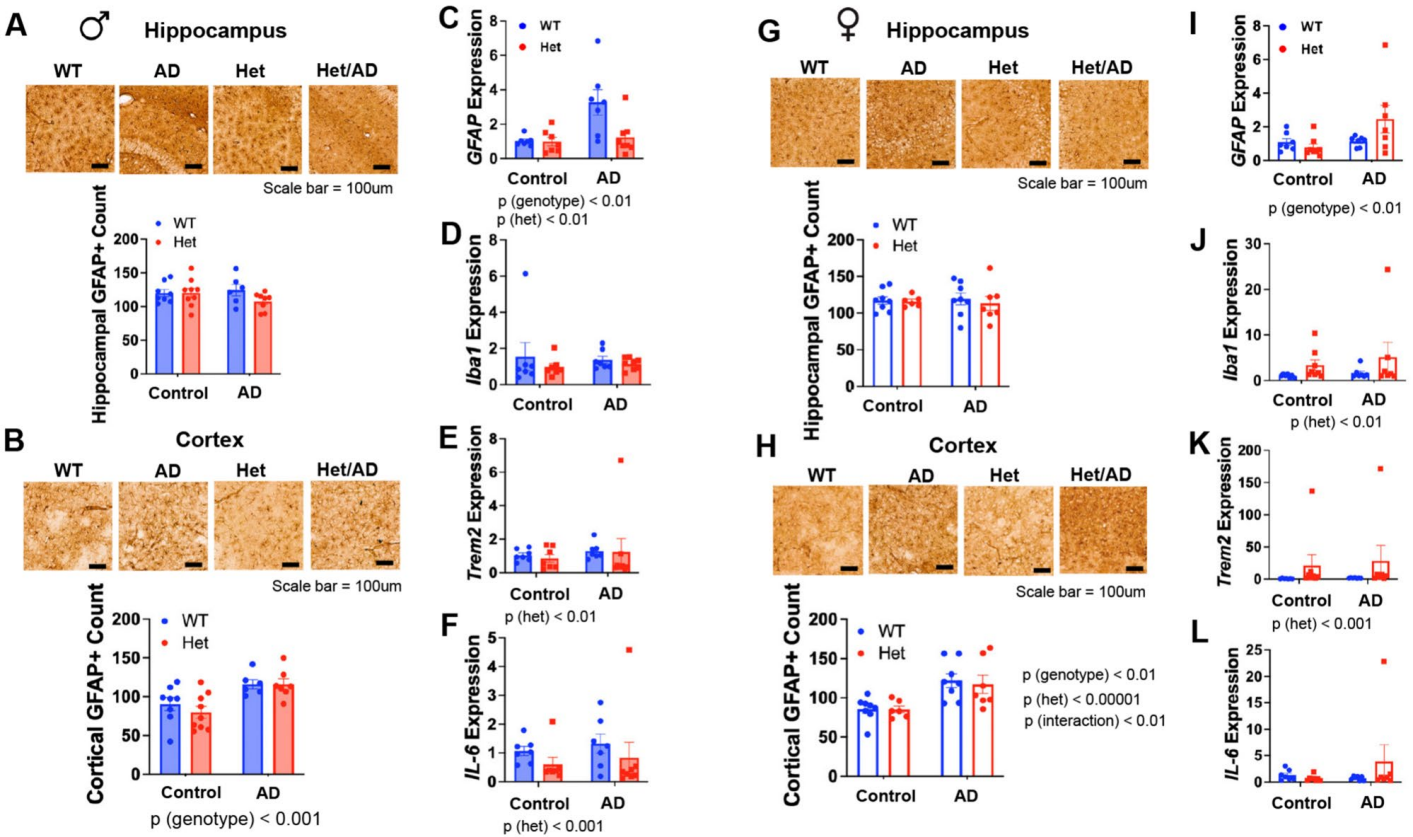
Reduced IGF-1 signaling fails to alter gliosis or inflammatory markers in male or female Tg344-AD rats. GFAP IHC staining was performed as a marker of gliosis in hippocampus and cortex as well as qPCR for other neuroinflammatory-related markers. (**A**,**B**) In males, while there was no difference among groups for GFAP positive counts in hippocampus, GFAP levels in cortex were increased in male TgF344-AD cortex versus WT (AD genotype p < 0.001). (**C**–**E**) Expression of neuroinflammatory-related markers detected an effect of AD genotype (p < 0.01) and *igf1r* genotype (p < 0.01) *for gfap*, an *igf1r* genotype effect for *trem2* (p < 0.01) and *il6* (p < 0.01), but no differences in *iba1.* (**G**,**H**) In females, GFAP counts were not different among groups in hippocampus, but a significant main effect of AD genotype (p < 0.01), *igf1r* genotype (p < 0.0001) and interaction term (p < 0.01) was observed for GFAP in cortex. (**I**–**L**) Gene expression via qPCR in cortex reveals significant main effects for *gfap* (AD genotype p < 0.01), *iba1* (*igf1r* genotype p < 0.01), and *trem2* (*igf1r* genotype p < 0.01) but not for *il6*. Data were analyzed by two-way ANOVA. Bar graphs represent mean ± S.E.M.

We further evaluated effects on supporting cells via astrogliosis. While there was no difference among groups for GFAP positive counts (Fig. 6A) GFAP levels in cortex were increased in male TgF344-AD cortex versus WT (Fig. 6B; p < 0.001), but levels were not affected by *IGF-1R* genotype per se. Moreover, when we assessed inflammatory-related markers in cortex by qPCR, there was an effect of AD genotype (p < 0.01) and *igf1r* genotype (p < 0.01) *for gfap* (Fig. 6C), no differences in *iba1* (Fig. 6D), but an *igf1r* genotype effect for *trem2* (Fig. 6E; p < 0.01) and il6 (Fig. 6F; p < 0.01) where levels were reduced in *igf1r* Hets. Similarly, while no differences were observed in hippocampal GFAP counts among groups (Fig. 6G; p < 0.001), a significant main effect of AD genotype (p < 0.01), *igf1r* genotype (p < 0.0001) and interaction term (p < 0.01) was observed for GFAP in cortex (Fig. 6H;, This corresponded with several interesting trends via qPCR in cortex, including for *gfap* (Fig. 6I; AD genotype p < 0.01), *iba1* (Fig. 6J; *igf1r* genotype p < 0.01), *trem2* (Fig. 6K; *igf1r* genotype p < 0.01) but not for *il6* (Fig. 6L). Moreover, MAP2 quantification for neuronal integrity via qPCR, Western blot or IHC was not notably impacted in either male or female cortex or hippocampus (Fig. S3).

### Sexually-dimorphic effects of *Igf1r* heterozygosity on the normal and AD brain proteome

**Fig. 7:**
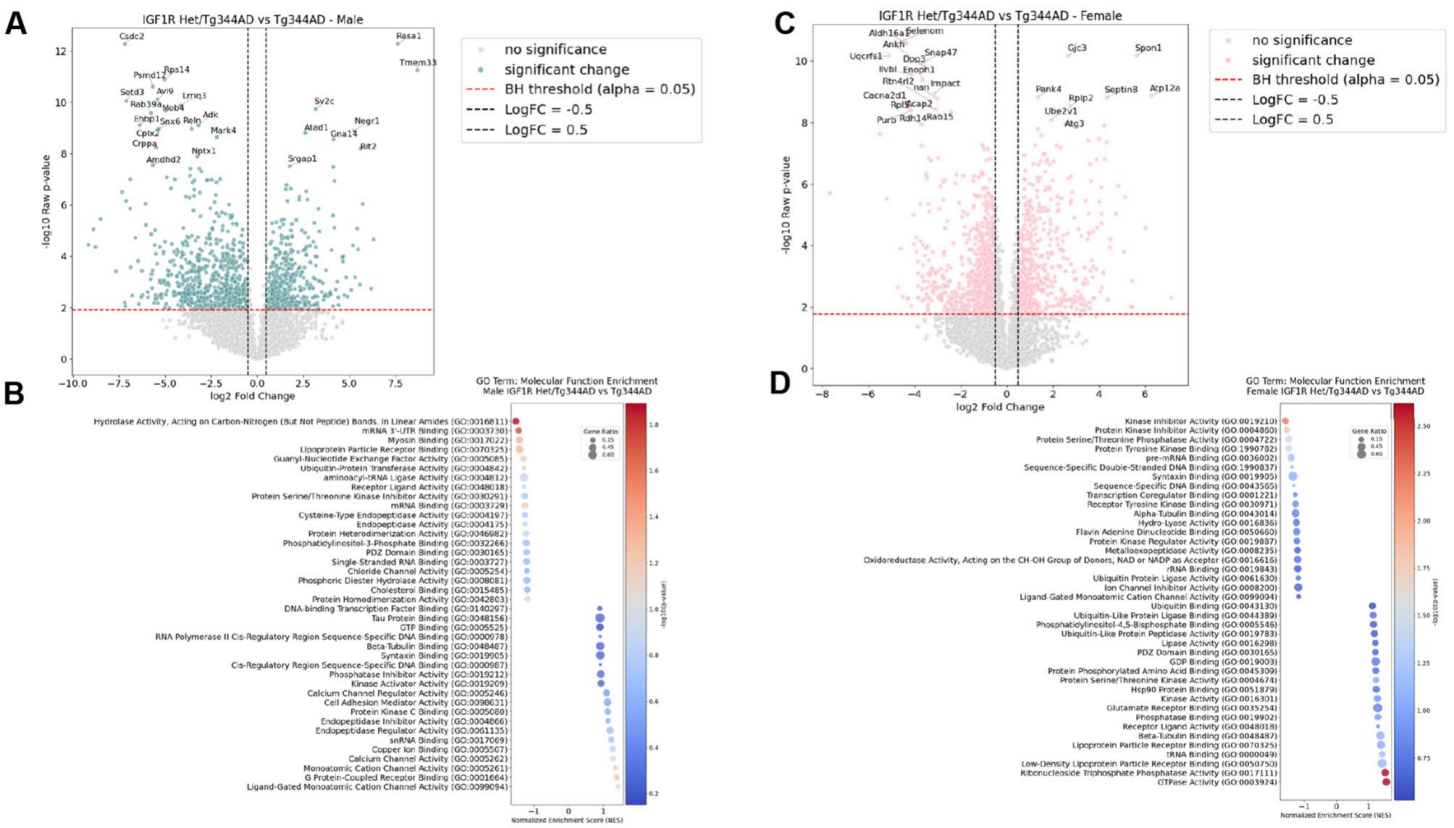
Effect of *Igf1r* heterozygosity on the hippocampal proteome in male and female Tg344 rats. In order to further determine the extent, if any, that *Igf1r* heterozygosity modulates the normal and/or AD brain, we performed proteomics analysis in hippocampus. (**A**) When comparing male AD groups, *Igf1r* Het AD rats, demonstrated a number of significantly down-regulated proteins, as compared toTgF344-AD rats, including a reduction in Cold Shock Domain Containing C2 (Csdc2), Reelin, Psmd12 and Rab32a activity. In contrast, Rasa1, a negative regulator of Ras-MAPK signaling, Negr1, Tmem33, Gna14 and Rit2, among others, were all significantly upregulated in AD Hets. (**B**) GO Pathway Analyses implicated a number molecular processes in *Igf1r* Hets, including an upregulation in Tau, GTP, and syntaxin binding, and a downregulation in lipoprotein particle receptor and cholesterol binding, respectively. (**C**) In females, *Igf1r* Hets demonstrated an array of differentially regulated proteins in AD, which were unique from male Het animals, including increased Spondin1 and Septin8, and downregulation of several redox-related proteins, including selenoprotein M, DPP3, Aldh16a1 as well as Uqcrfs1. (**D**) GO pathway analysis revealed enrichment in Het females for a number of pathways involved in ubiquitin metabolism, Hsp90 binding, and lipoprotein particle receptor binding.

In order to further determine the extent, if any, that *Igf1r* heterozygosity modulates the normal and/or AD brain, we performed proteomics analysis in hippocampus. PCA confirmed that samples were strongly stratified by sex, while *Igf1r* Het males tended to cluster from Control males (Fig. S4A) which can be further visualized via heatmap (Fig. S4B,C). When comparing male AD groups, *Igf1r* Het AD rats, demonstrated a number of significantly down-regulated proteins, as compared toTgF344-AD rats (Fig. 7A). Of note, a striking downregulation was observed for Cold Shock Domain Containing C2 (Csdc2), an RNA-binding protein, Reelin, an extracellular matrix glycoprotein linked to synaptic integrity and cognitive resilience to AD, as well as proteins involved in proteosomal (Psmd12) and lysosomal (Rab32a) activity. In contrast, proteins such as RAS p21 protein activator 1 (Rasa1), a negative regulator of Ras-MAPK signaling, Neuronal Growth Regulator 1 (Negr1), as well as proteins involved in ER stress, (Tmem33), and G Protein signaling (Gna14; Rit2) were all significantly upregulated in AD Hets. GO Pathway Analyses implicated a number molecular processes in igf1r Hets, including an upregulation in Tau, GTP, and syntaxin binding, and a downregulation in lipoprotein particle receptor and cholesterol binding, respectively (Fig. 7B).

As opposed to male animals, *Igf1r* Het females demonstrated a unique array of differentially regulated proteins in AD. These included an upregulation in Spondin1 and Septin8, both involved in extracellular matrix integrity, and linked to AD. In contrast, selenoprotein M, DPP3 (Dipeptidyl Peptidase 3) and Aldh16a1 (Aldehyde Dehydrogenase 16 Family Member A1), all factors involved in redox balance, as well as Ubiquinol-Cytochrome c Reductase, Rieske Iron-Sulfur Polypeptide (Uqcrfs1), a critical subunit of mitochondrial complex III, IMPACT RWD domain-containing protein, which is involved in the integrated stress response, Cacna2d1, a modulator of calcium handling, and Enolase-phosphatase 1, which is involved in methionine metabolism, were all significantly downregulated (Fig. 7C; p < 0.05). This corresponded with enrichment for a number of pathways involved in ubiquitin metabolism, Hsp90 binding, and lipoprotein particle receptor binding, among others (Fig. 7D). Interestingly, while *Igf1r* heterozygosity resulted in a number of significantly up- and down-regulated proteins in WT (non-AD) males (Fig. S4D), only one protein, Rpl29, was significantly regulated in female Het hippocampus (Fig. S4E), and these results led to disparate effects via pathway analysis in hippocampus of non-AD male and female animals, respectively (Fig. S4F,G).

### Effect of AD and *Igf1r* heterozygosity on behavior and cognition

**Fig. 8:**
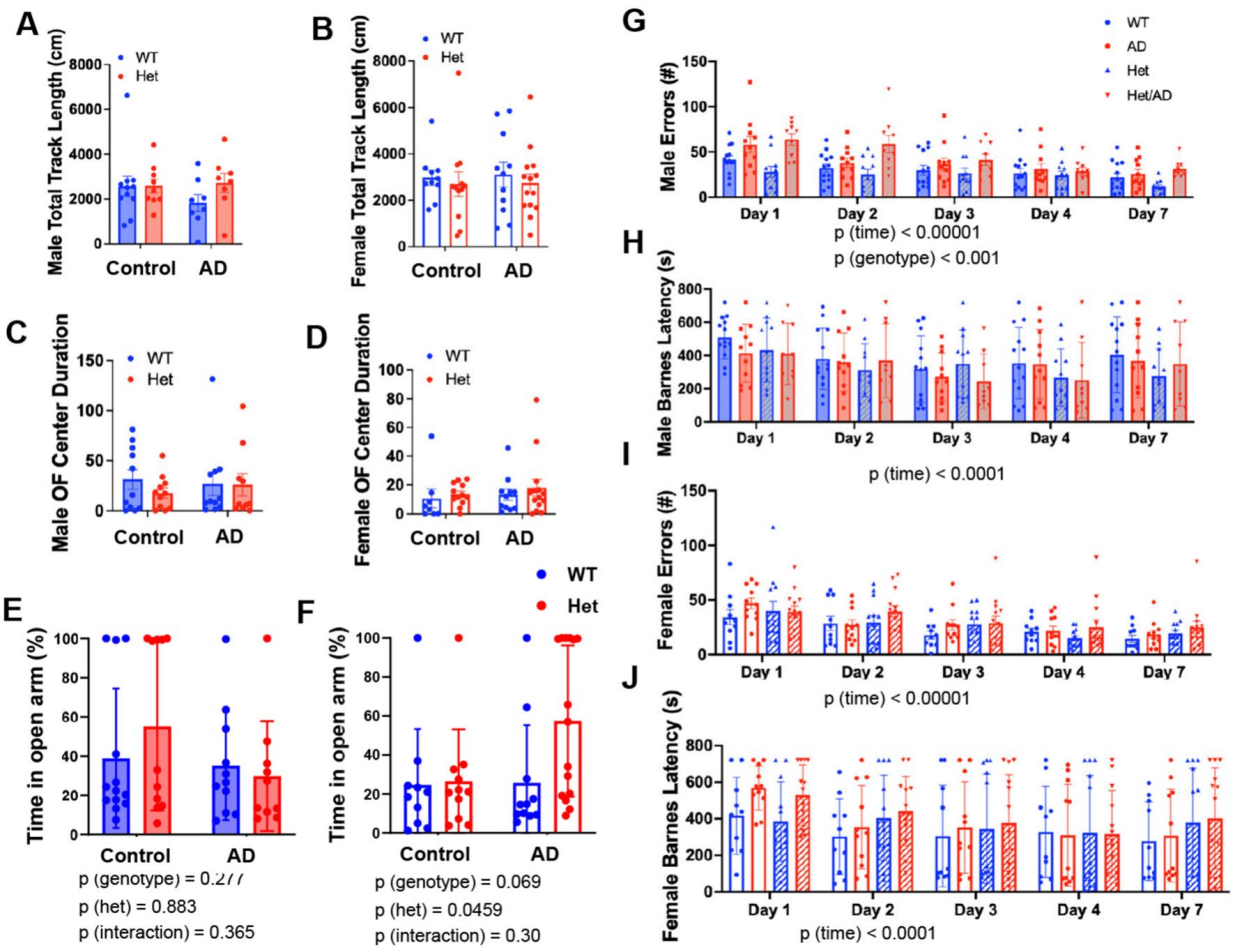
Effect of *Igf1r* Heterozygosity on cognitive and behavioral function in male and female AD rats. The effect of the TgF344-AD genotype and *igf1r* status on behavior and cognitive function in male (WT n = 13; *igf1r* Het n = 11; AD n = 11; Het-AD n = 10) and female groups (WT n = 10; *igf1r* Het n = 11; AD n = 12; Het-AD n = 14) were assessed in a blinded fashion. (**A**–**D**) Initially, animals were evaluated in the Open Field for 9 min, where no differences were detected among groups for total track length or time spent in the center of the arena in either sex. (**E**,**F**) The elevated plus maze was conducted to assess anxiety-like behavior. In males, there were no significant differences among groups for percent of time spent in the open arm. Interestingly, nearly half of Het/AD rats spent the entire duration of testing in the open arm, but this was not significant among groups. (**E**,**F**) Barnes maze was conducted to assess learning and memory, In males, TgF344-AD animals appeared to make greater errors at Day 1, irrespective of *igf1r* genotype However, there was no evidence of significantly impaired learning (days 2–4) or retention (day 7) among groups via number of errors or latency to escape. In females, while the number of errors committed during the studies did not differ among groups, TgF344-AD females did seem to have a delayed latency to escape at day 1, though no differences were observed among groups at subsequent time points. Open field and Elevated Plus Maze data were assessed by two-way ANOVA. Barnes Maze was assessed by two-way ANOVA with repeated measures on time. Bar graphs represent mean ± S.E.M.

Finally, we assessed the effect of the TgF344-AD genotype and IGF-1R status on behavior and cognitive function (Fig. 8). Initially, animals were evaluated in the Open Field for 9 min. There were no differences among groups for total track length or time spent in center of the arena in either male or female animals (Fig. 8.A–D). We further assessed anxiety-like behavior via the elevated plus maze. In males, there were no significant differences among groups for percent of time spent in the open arm (Fig. 8E). In females, nearly half of Het/AD rats spent the entire duration of testing in the open bright arm, but this was not significant among groups (Fig. 8F). In order to assess learning and memory, Barnes maze was also performed. In males, TgF344-AD animals appeared to make greater errors, particularly at Day 1, and this was significant when averaged across trials (p (genotype) < 0.0001), irrespective of *Igf1r* genotype (Fig. 8G). However, there was no evidence of significantly impaired learning (days 2–4) or retention (day 7) among groups via number of errors or latency to escape (Fig. 8G–I). In females, while the number of errors committed during the studies did not differ among groups, TgF344-AD females did tend to have a delayed latency to escape at day 1, though no differences were observed among groups at subsequent time points (Fig. 8J).

## Discussion

While studies in mice establish the potential importance of the GH/IGF-1 signaling pathway to human aging^7–9,11,37^, the extent to which this pathway can be causally and mechanistically interrogated has not been well explored in higher species. To address this gap, we generated an *Igf1r* haploinsufficient rat model to study effects of this pathway on physiology and disease. As such, this study reveals that *Igf1r* Het rats phenocopy some metabolic traits previously observed in their mouse *Igf1r*^+*/−*^ counterparts, namely a comparable reduction in body size and energy expenditure, without notable impairments to glucose homeostasis. However, when we evaluated the functional consequence of *Igf1r* deficiency in a rat model of AD, we unexpectedly observed that *Igf1r* Het rats failed to recapitulate the reduction in amyloid pathology often reported in comparable AD mouse models with *Igf1r* haploinsufficiency.

Interestingly, work by our lab and others have suggested that as opposed to lowering brain IGF-1, a localized increase in central nervous system (CNS) IGF-1 levels, either via transgenic, intranasal delivery or direct injections of peptide, confers benefits to the aging brain, though these effects may be more favorable in male animals^38^. More recently, we found that intranasal delivery of a potent IGF-1 analogue improved some facets of pathology in male 5XFAD mice, including promotion of plaque compaction, which corresponded with a reduction in filamentous plaques, an increase in inert plaques, and a reduction in low molecular weight Aβ oligomers^39^. In vitro, uptake of Aβ (1–42) peptide by BV2 cells was also enhanced by IGF-1 analogue, supporting the notion that these effects may be mediated in part via boosting microglia function. Given these prior findings, it is somewhat intriguing in the present study that *Igf1r* het females, irrespective of AD genotype status, demonstrated higher expression of both *iba1* and *trem2* levels in cortex, which may indicate altered microglial function, a possibility further supported by the accretion of small plaques when crossed with an amyloidosis model, a finding which is appears to be at odds with a prior report of an attenuation in small plaques in neuronal-specific *IGF-1R* deletion in *APP/PS1* mice. Moreover, genetic activation of mTOR, which is in part downstream of IGF-1 signaling, is reported to trigger Trem2 activity and enhanced amyloid clearance^40^, whereas Trem2 loss-of-function mutations, such as *R47H*, are commonly associated with AD. In contrast, isolated IGF-1 deficiency leads to impairments in neurovascular coupling^41,42^, adaptive cerebral arterial remodeling^43^, mitochondrial function in astrocytes, and visuospatial memory^44^. Thus, these studies highlight that IGF-1 signaling in the brain—and its effects on diverse cell types, physiological processes, and disease states, including AD—is inherently complex, with outcomes that can be influenced by sex, species, and other nuances, making it unlikely that IGF-1 action in the brain simply conforms to a “one-size-fits-all” description.

Consistent with numerous studies in mice^7,8^, while *Igf1r* heterozygous animals had similar weights as controls during early development and adolescence, body weight was ~ 15% reduced by adulthood in both male and female rats. However, the > 50% reduction in IGF-1Rs in these animals did not result in a significant elevation in circulating IGF-1 levels in either sex, at least at younger ages. However, IGF-1 and insulin levels were relatively elevated in *Het* females at 15 mo, regardless of AD status. When rats were clamped at 6 mo of age, there was no detectable impairment in whole-body insulin action or glucose fluxes in either sex. This is consistent with studies in *Igf1r*^+*/−*^ mice utilizing proxy assays of glucose and insulin tolerance^8^, where relative impairment is not observed in younger animals, but worsened glucose tolerance and insulin resistance emerges in older *Igf1r*^+*/−*^ mice^8,12^. To this end, one limitation of our study is that euglycemic clamps were not performed at older ages. It is worth noting that mid-aged females appear to be slightly hyperinsulinemic, though the extent to which a reduction in IGF-1Rs is consequential for maintaining glucose homeostasis in aged rats is not yet clear.

Another observation from this study was several noted sex-dependent effects in the biological response to attenuated IGF-1R signaling, which adds to a growing list of reported sex differences in this axis. This was highlighted by female-specific effects of *Igf1r* haploinsufficiency on pathology, hormonal levels, microglial markers, along with a male-specific downregulation in GSK3β activation in cortex. A proteomic analysis in hippocampal tissue further revealed a large number of proteins found to be uniquely altered in male versus female *Het*/AD tissue, including markedly lower levels of several proteins in *Het*/AD females involved in redox metabolism. Meanwhile, there was an astonishingly near absence of significantly impacted proteins in non-AD/*Het* female hippocampus, as compared to a much more robust number of differentially-expressed proteins in males Hets.

While the reasons for these differences are unclear, a culmination of research over the past several decades has determined that sex differences across biologic processes are likely attributable to a multitude of factors, including hormonal activation effects, organizational effects, and sex chromosome contributions. Nevertheless, an important limitation here and elsewhere with female rodent studies is that the relatively young age of disease onset coupled with stark menopausal differences between rodents and humans, likely obscures the full extent of aging and menopausal state to the etiology of AD. Indeed, even though female rodents often become reproductively senescent by ~ 9 months of age, estrogen levels can remain quite high until very advanced ages in many strains (> 20 mo of age)^45,46^, which does not accurately recapitulate the physiology or aging context of human menopause. Greater incorporation of age-appropriate ovariectomized (OVX) models or chemically-induced menopause (VCD) strategies, in conjunction with more aging-relevant models of late-onset AD, could help to better address these limitations.

In summary, using a novel rat model of *Igf1r* heterozygosity, we find that some aspects of constitutively reduced IGF-1R levels, appear to be conserved, including a reduction in body weight by adulthood, without notably impairing insulin sensitivity, at least at younger ages. In contrast, when crossed with a rat model of AD, our data also show that lowering IGF-1 signaling per se fails to confer protection against AD-related pathology, and may even exacerbate some facets of disease in females. Moreover, a number of other sex differences were also noted with *Igf1r* heterozygosity, further emphasizing the GH/IGF-1 axis as prototypical example of sex differences in aging and disease. It is unclear why rats harboring the same *APP/PSEN* mutations respond differently to low IGF-1R signaling than mice, but the TgF344 rat model has proven amendable to other interventions, including exercise^47^, low-dose radiation^48^ and drugs^49^, while disease is accelerated by chronic stress^50^ or obesity^51^. Thus, the implications of this study support a more nuanced relationship of IGF-1 tone and AD than has been previously appreciated in other models and species^34,35,52^. Indeed, while the longstanding sentiment has been that excess GH/IGF-1 accelerates aging, recent human observation studies have emerged that illuminate that this link is not simply a linear one, but rather can exist as a U-shape relationship that can vary by sex and age^52^. Given the importance of the GH/IGF-1 axis as a potential therapeutic target, further interrogation of this pathway, and its temporal and spatial role in metabolic health and AD pathogenesis, particularly with newly-developed AD strains, is warranted. Such efforts should also continue to account for sex differences, hormonal status, and the unique contribution of specific CNS cell types to collectively aid in better defining a more precise role of growth factor signaling in CNS health and disease throughout the life course.

## Supplementary Information

Supplementary Information.

## Data Availability

Data are freely available from the corresponding author upon reasonable request. Sequencing data are available in Figshare repository under Digital object identifier (DOI) 10.6084/m9.figshare.27853899 The proteomics raw and result files are available in ProteomeXchange (PRIDE) [https://www.ebi.ac.uk/pride/archive/projects/PXD068498] under the accession PXD068498.
