# Supplementary material for: Reduced IGF-1 signaling fails to limit Alzheimer’s disease progression in a novel rat model of IGF-1R haploinsufficiency
Source: Sci Rep. 2025 Dec 16;16:1856. doi: 10.1038/s41598-025-31601-1 (PMC12804757; doi:10.1038/s41598-025-31601-1)

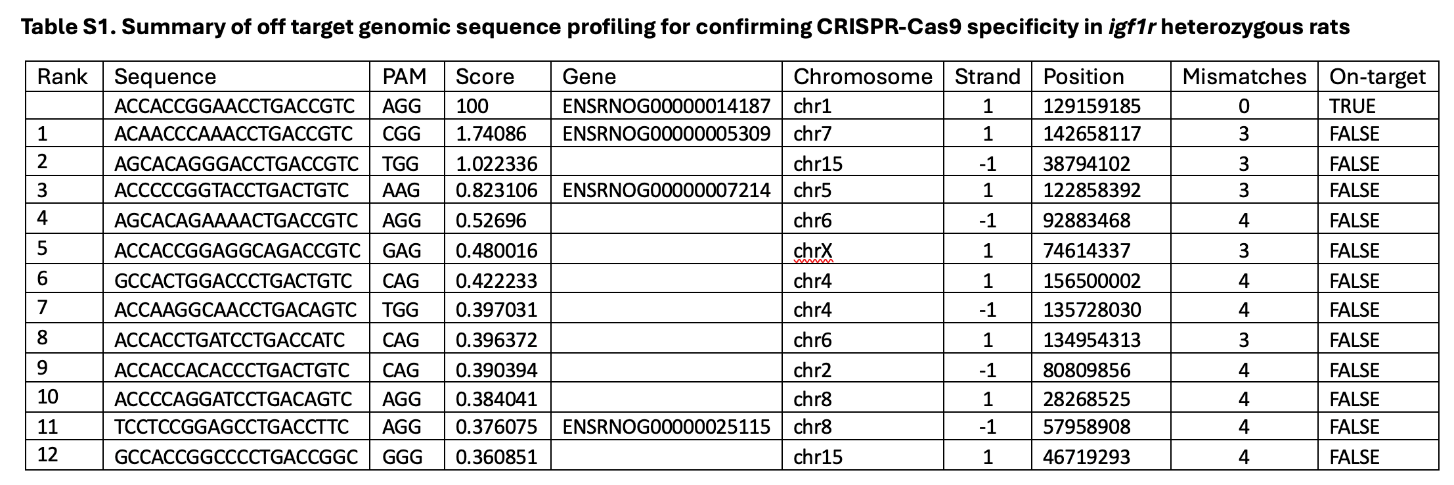
**Table S1.** Summary of off target genomic sequence profiling for confirming CRISPR-Cas9 specificity in igf1r heterozygous rats


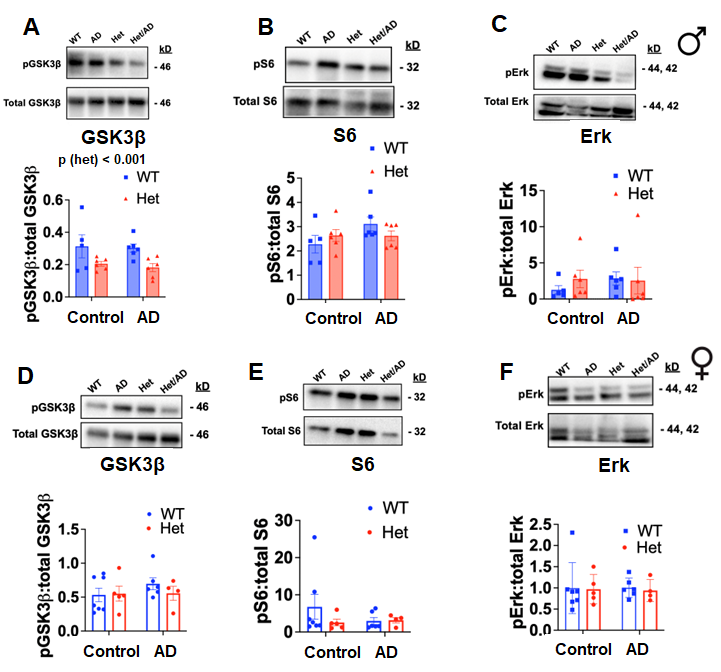


**Figure. S1. Metabolic signaling characteristics in cerebral cortex of wild type control and TgF344 rats (A-C)** In males, *igf1r* haploinsufficiency was confirmed to lower GSK3β activation, regardless of AD status, but no differences were observed in phospho-S6 to Total S6 or phospho-Erk Total Erk (WT n=6; *IGF-1R* Het n=6; AD n=6; Het-AD n=6). (**D-F**) In females, there were no differences detected in GSK3β, S6 or Erk activation (WT n=6; *igf1r* Het n=6; AD n=6; Het-AD n=6). Data analysis was performed via two-way ANOVA. Bars represent mean ± S.E.M.


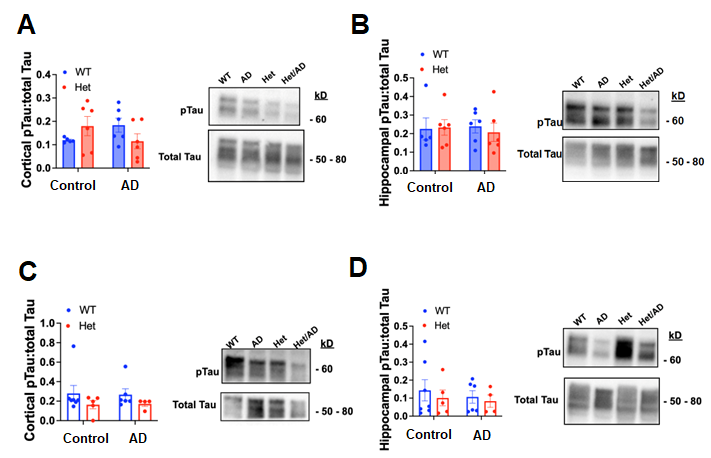


**Figure. S2. Effect of *Igf1r* heterozygosity on Tau phosphorylation in brain tissue. (A-B**) In males, no effect was observed on tau phosphorylation in either cortex or hippocampus among groups**.** (WT n=6; *IGF-1R* Het n=6; AD n=6; Het-AD n=6). (WT n=6; *igf1r* Het n=6; AD n=6; Het-AD n=6**). (C-D)** In females, no effect was observed on tau phosphorylation in either cortex or hippocampus among groups. Data analysis was performed via two-way ANOVA. Bars represent mean ± S.E.M.


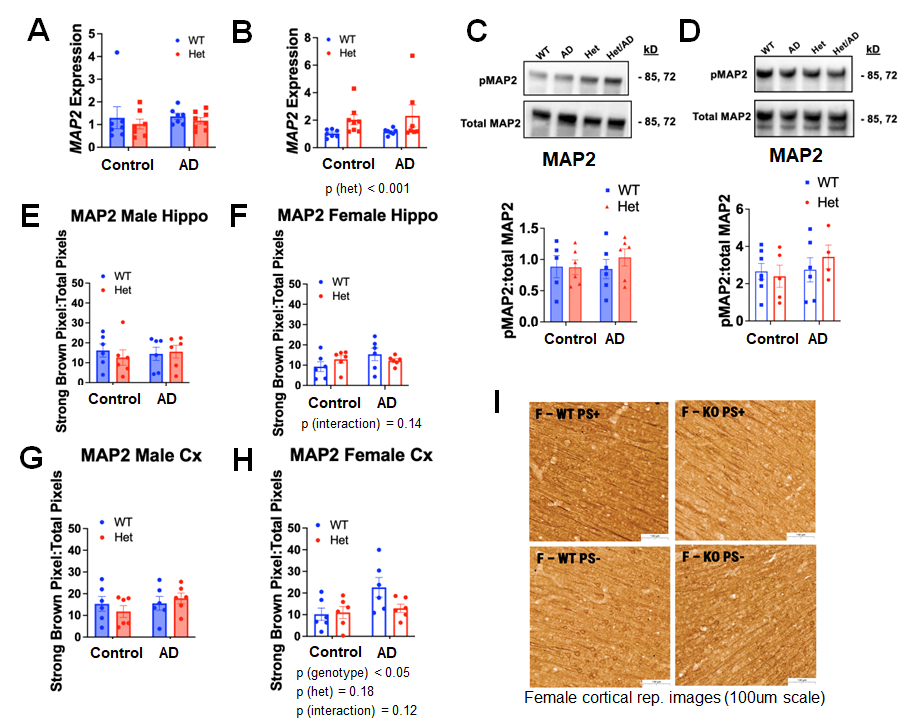


**Figure. S3. Quantification of MAP2 levels in the cortex and hippocampus of wild type control and TgF344 rats harboring one or two copies of the *igf1r* gene.** In males, MAP2 quantification for neuronal integrity was evaluated via orthogonal assays. (**A-B**) In cortex, while no differences were observed in males, a significant *igfr1* genotype effects was observed in females (p<0.001), (**C-D**) Western blot for MAP2 levels in males and females, however, did not uncover any significant main effects or interaction effect. (**G-I**) In males, IHC staining in hippocampus and cortex did not detect differences, but MAP2 levels in females detected a significant main effect in cortex for AD genotype (p<0.05), but no significant effect of *igf1r* status or interaction. Data analysis was performed via two-way ANOVA. Bars represent mean ± S.E.M.


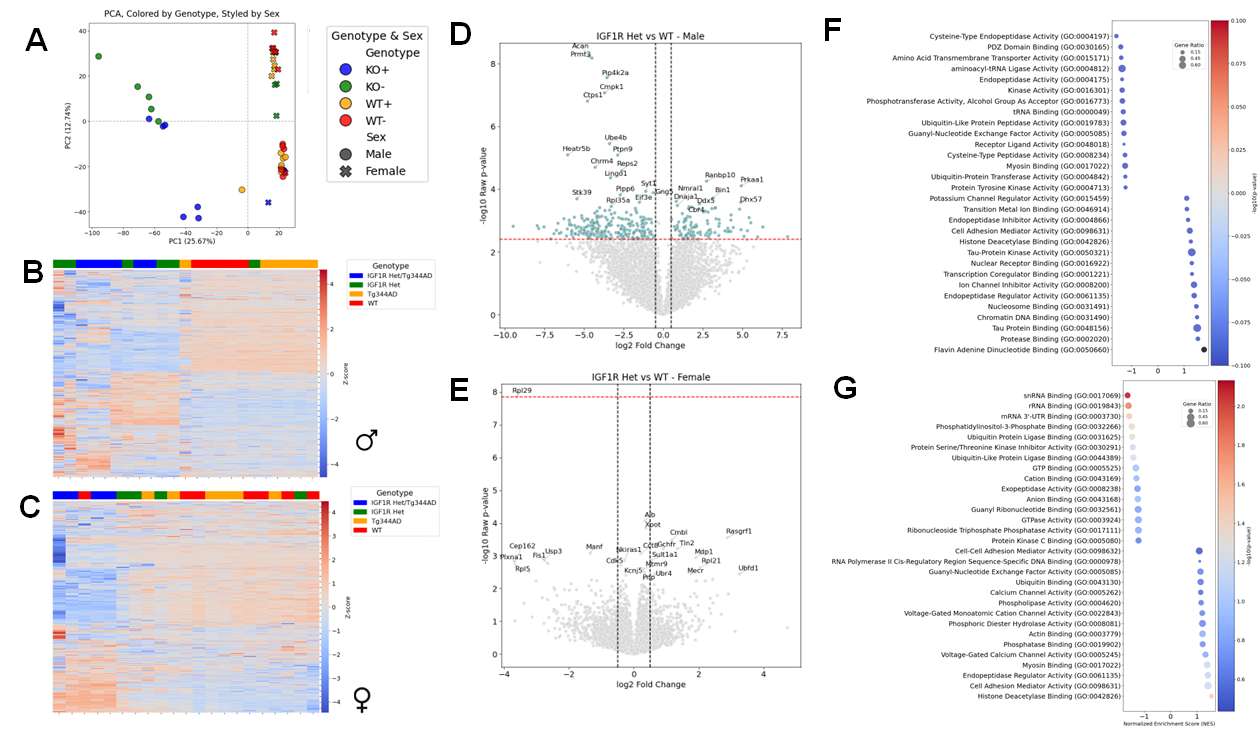


**Figure. S4. Effect of *Igf1r* heterozygosity on the hippocampal proteome in male and female Tg344 rats.** In order to evaluate the effect of *Igf1r* heterozygosity to modulate the normal and/or AD brain, we performed proteomics analysis in hippocampus. (**A**) PCA confirmed that samples were strongly stratified by sex, while *Igf1r* Het males tended to cluster from Control males along PC1. (**B**) Heat maps further confirm that male samples largely cluster together, with *Het* animals further divergent from WT. (**C**) In females, *Igf1r* Het/AD animals tended to cluster together, separate from other groups. (**D-E**) Volcano plots confirm that >100 proteins were significantly different between male WT and Het animals. In contrast, only Rpl29 reach significance between female groups.(F-G) GO pathway terms for molecular processes either up- or down regulated in Het males and females, respectively, as compared to Controls.

**Narayan et al uncropped blots**


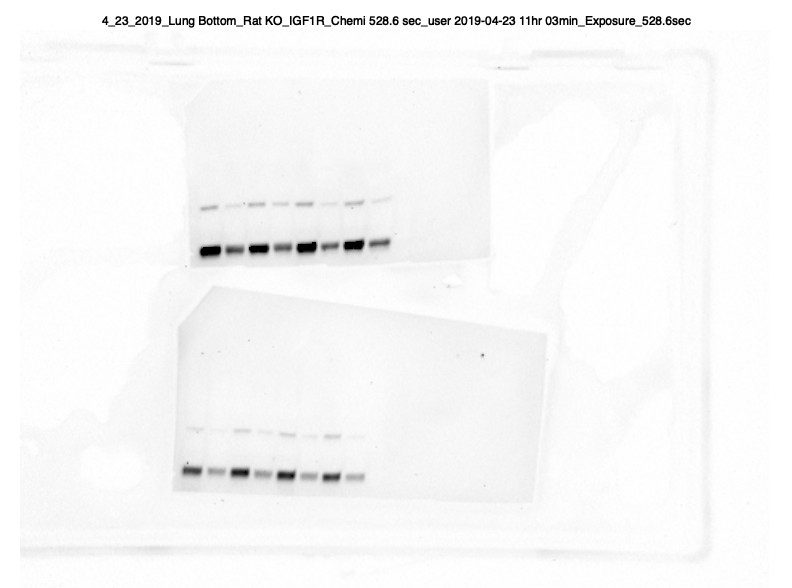


Fig. 2A Lung IGF-1R


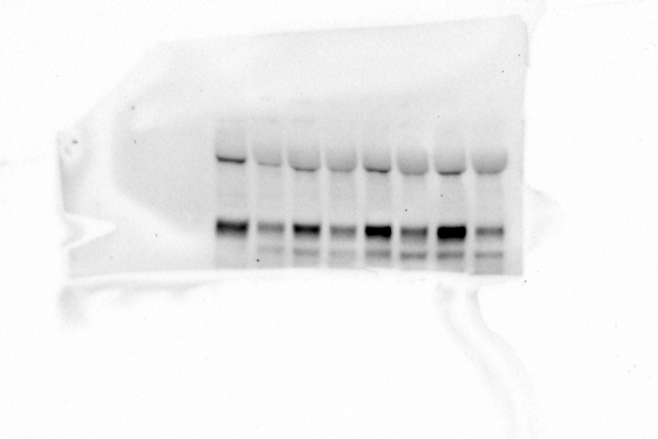


Fig. 2A Heart IGF-1R


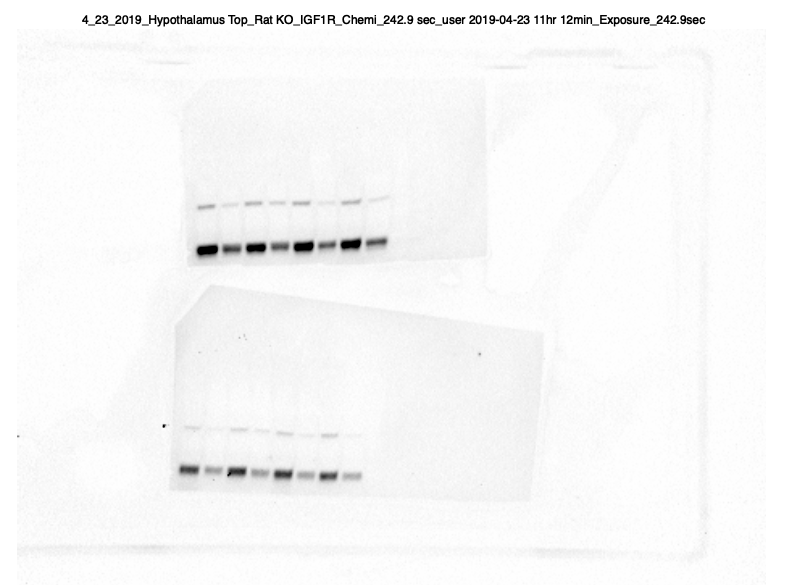


Hypothalamus IGF-1R


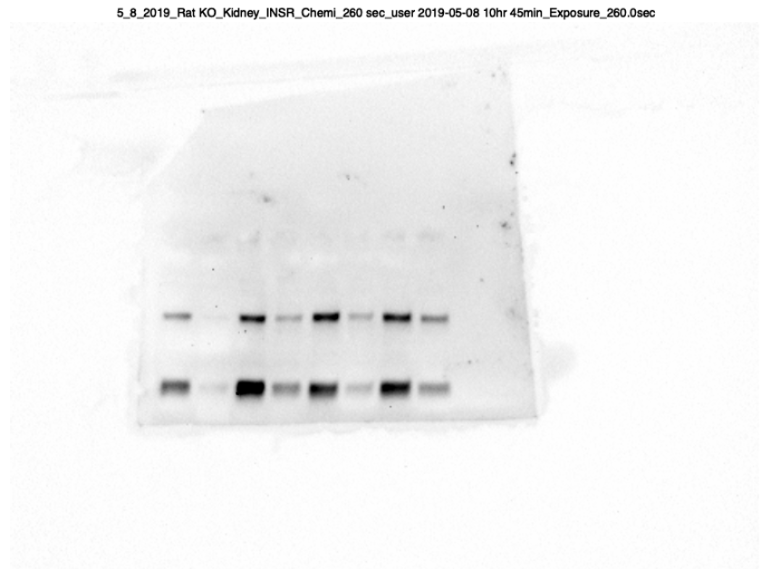


Kidney IGF-1R


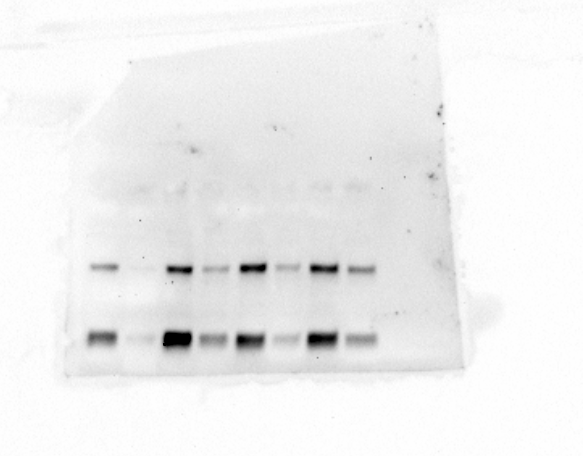


Fig. 2A Kidney IGF-1R


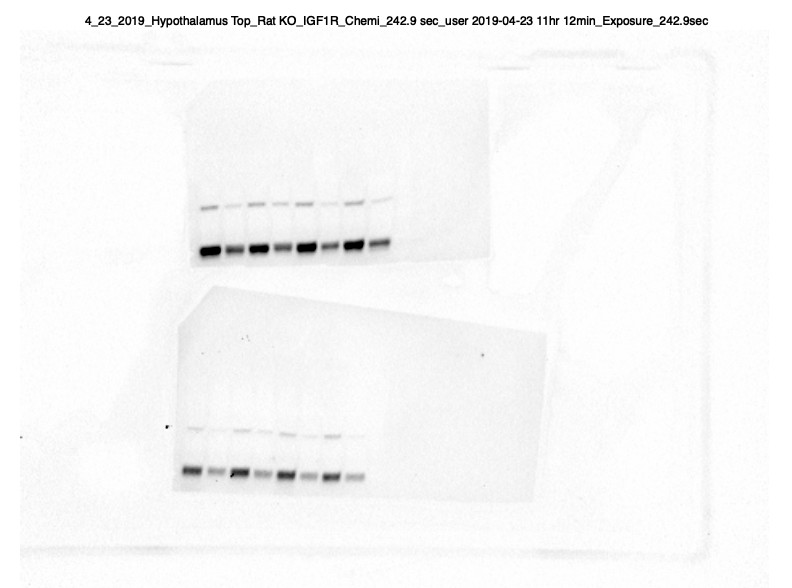


Fig. 2A Hypothalamus IGF-1R


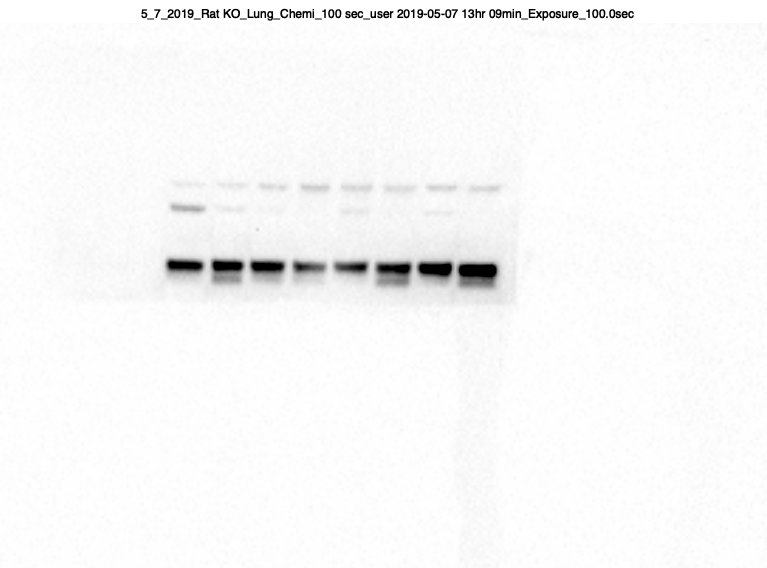


Fig. 2C Lung InsR


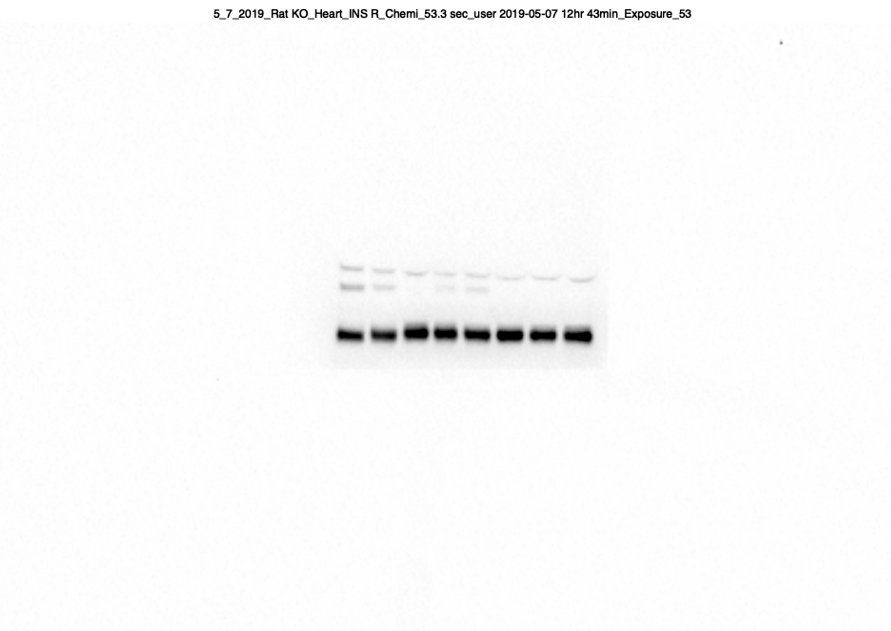


Heart InsR


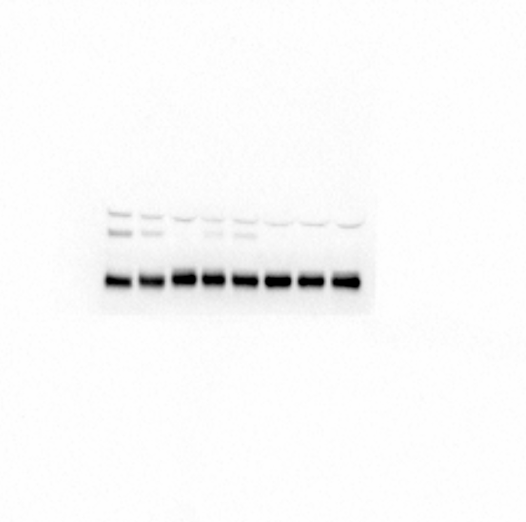


Fig. 2C Heart InsR


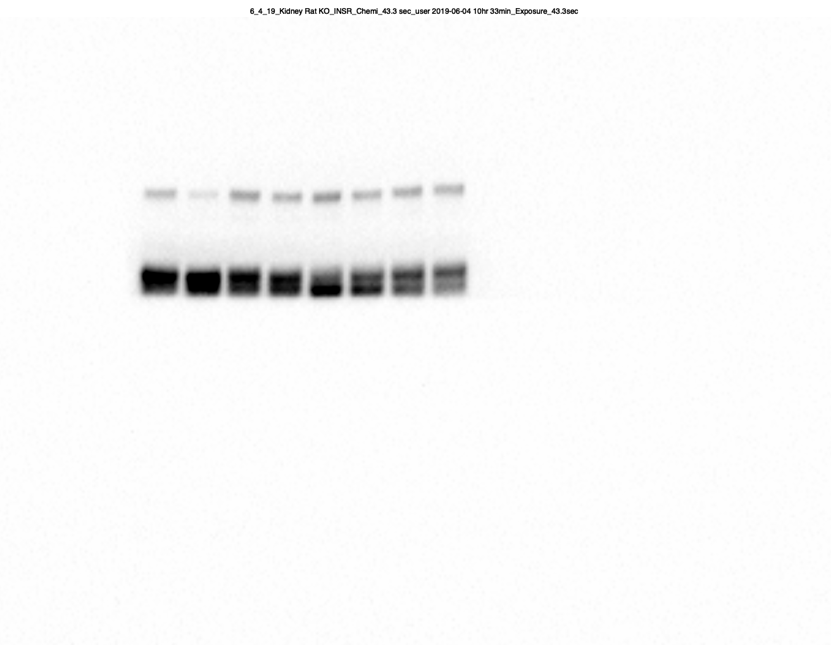


Fig. 2C Kidney InsR


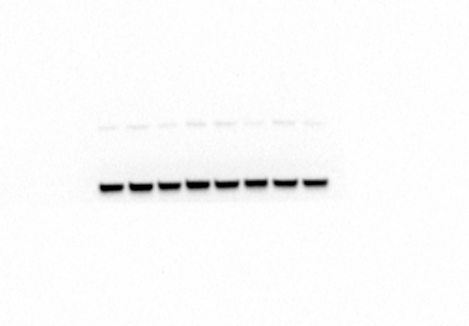


Fig. 2C Hypothalamus InsR


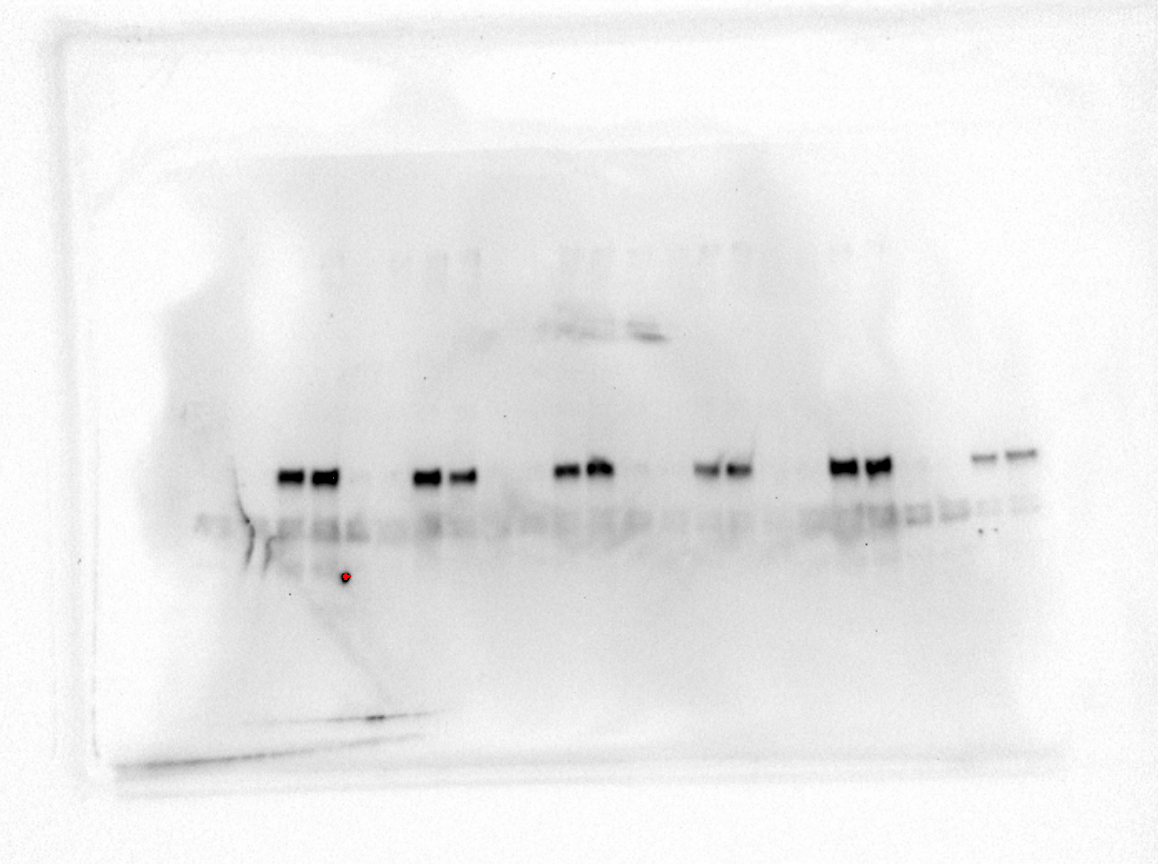


**Fig 2E pAkt**

**Fig 2E pAkt**


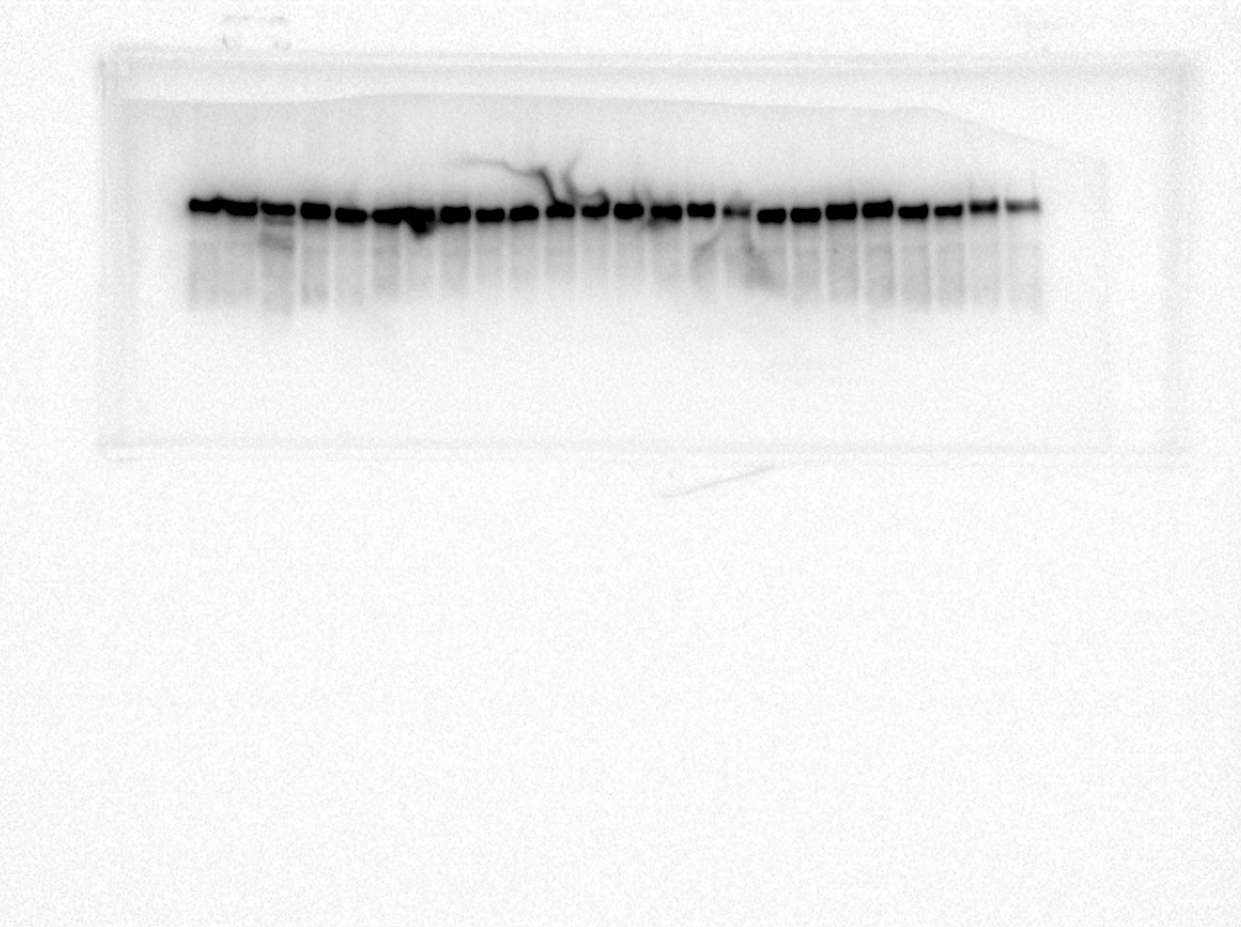


**Fig 2E Akt**


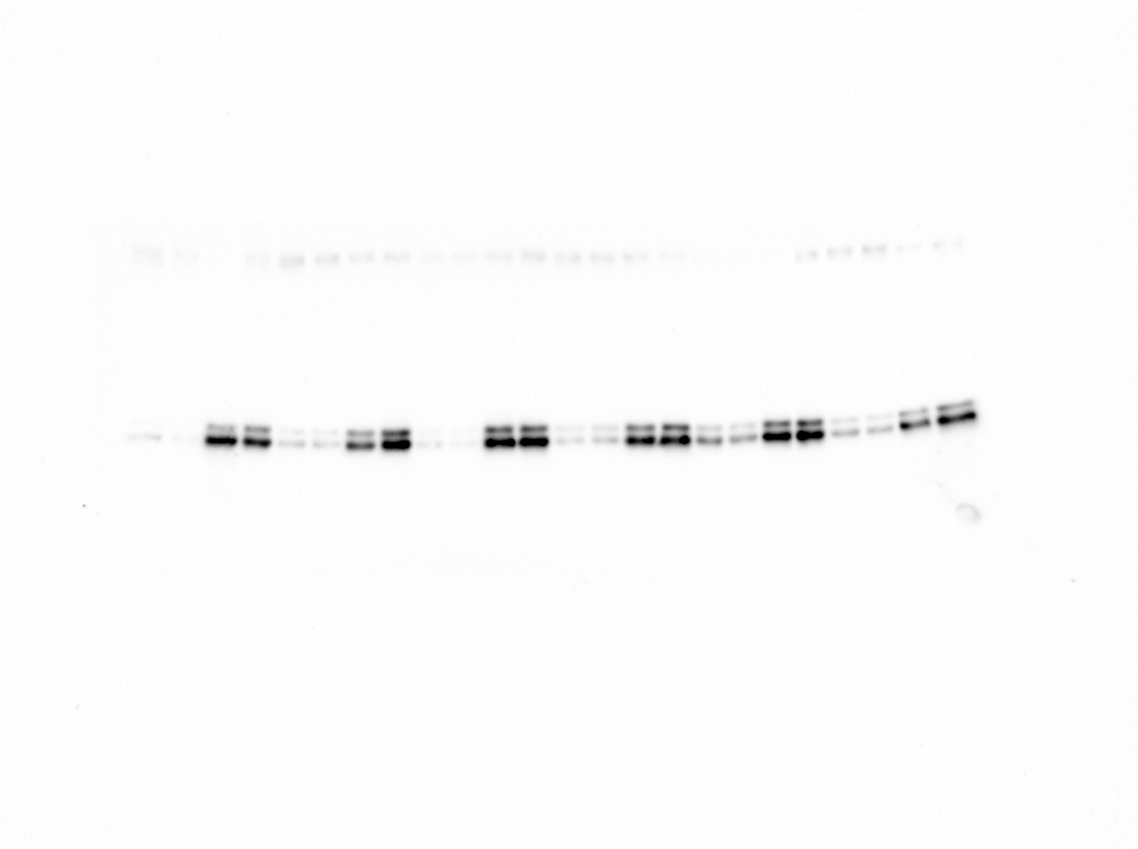


**Fig 2E pErk**


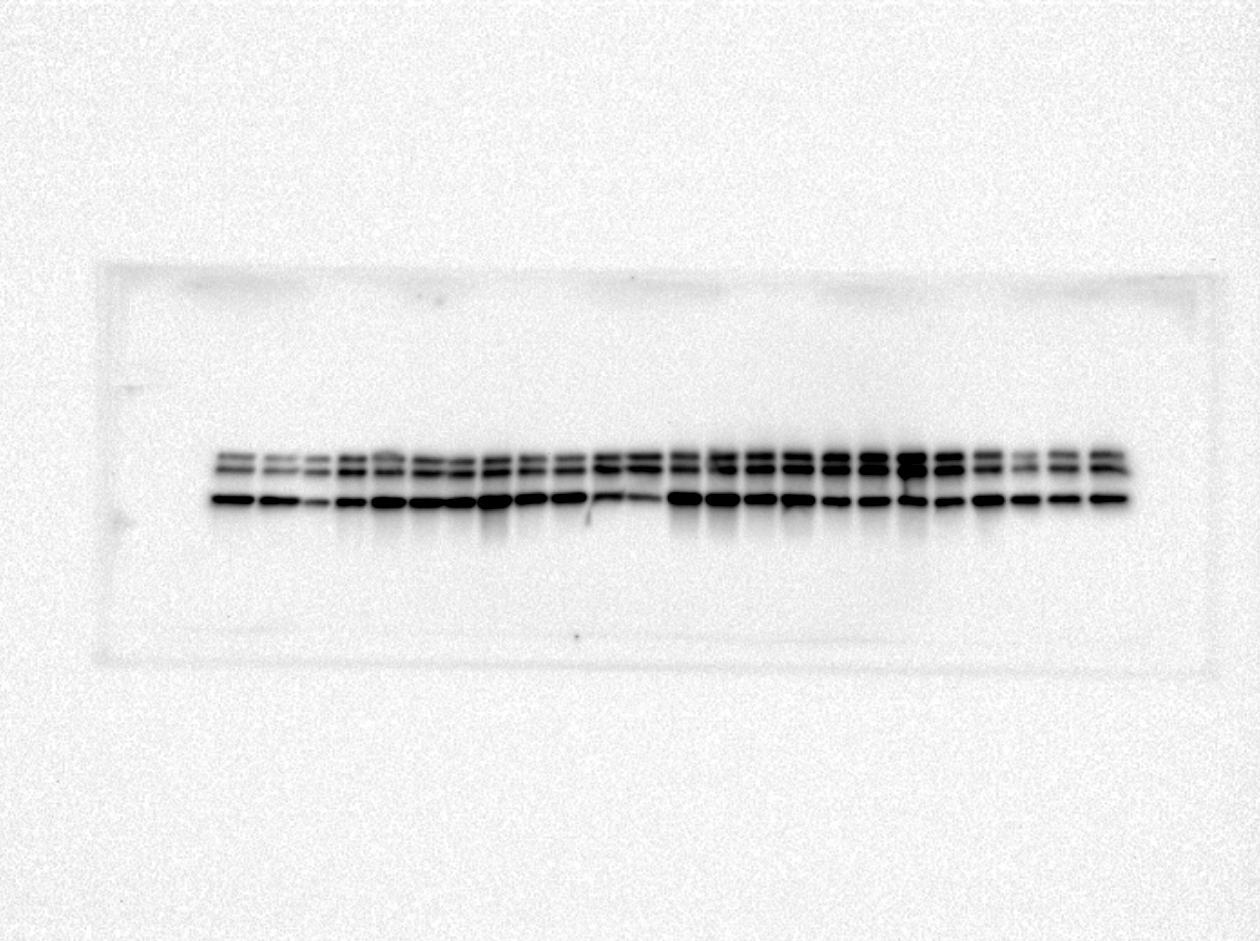


**Fig 2E Erk**


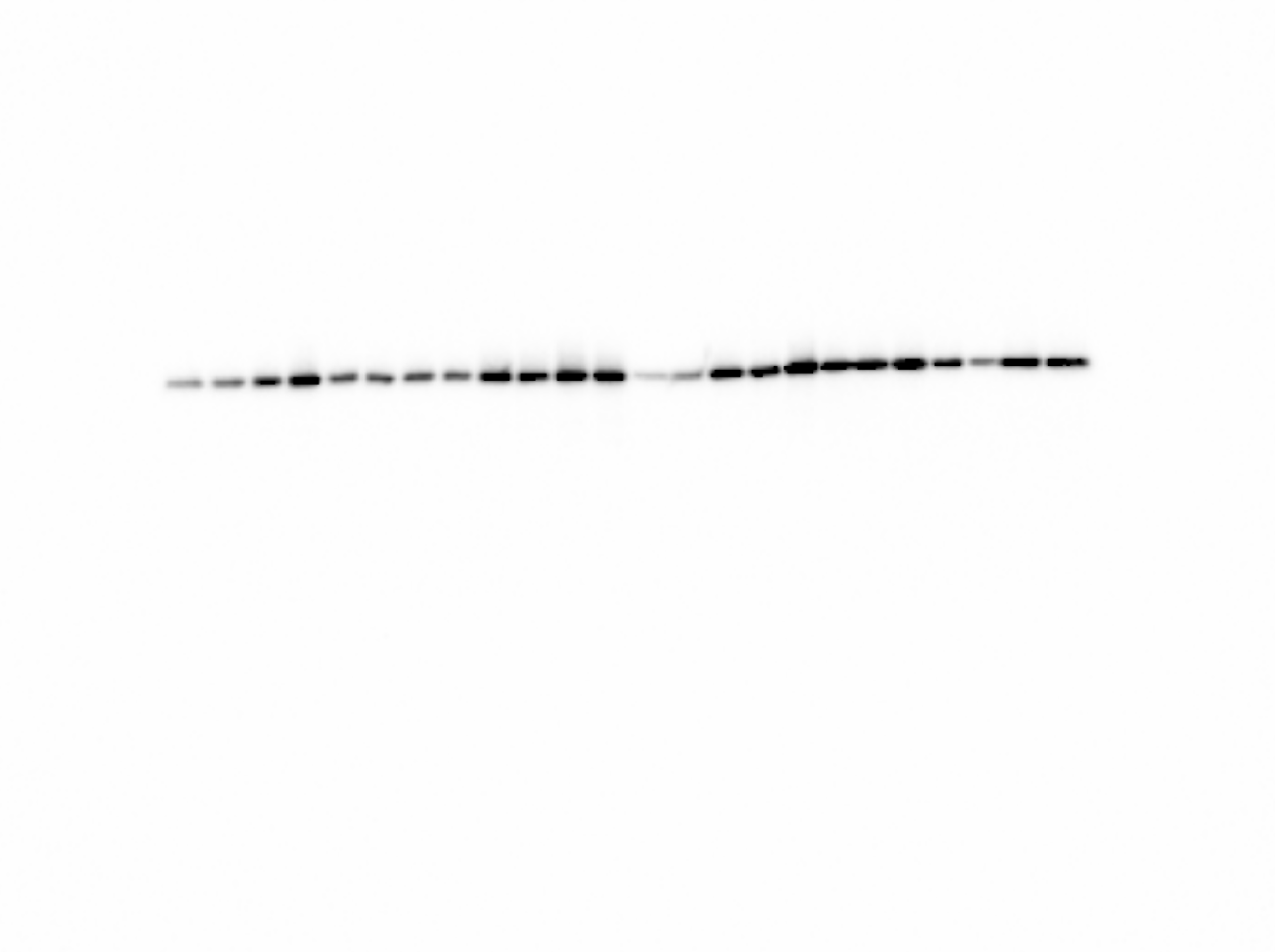


**Fig 2E pS6**

*Note pS6 image reached first pixel saturation at 4 seconds


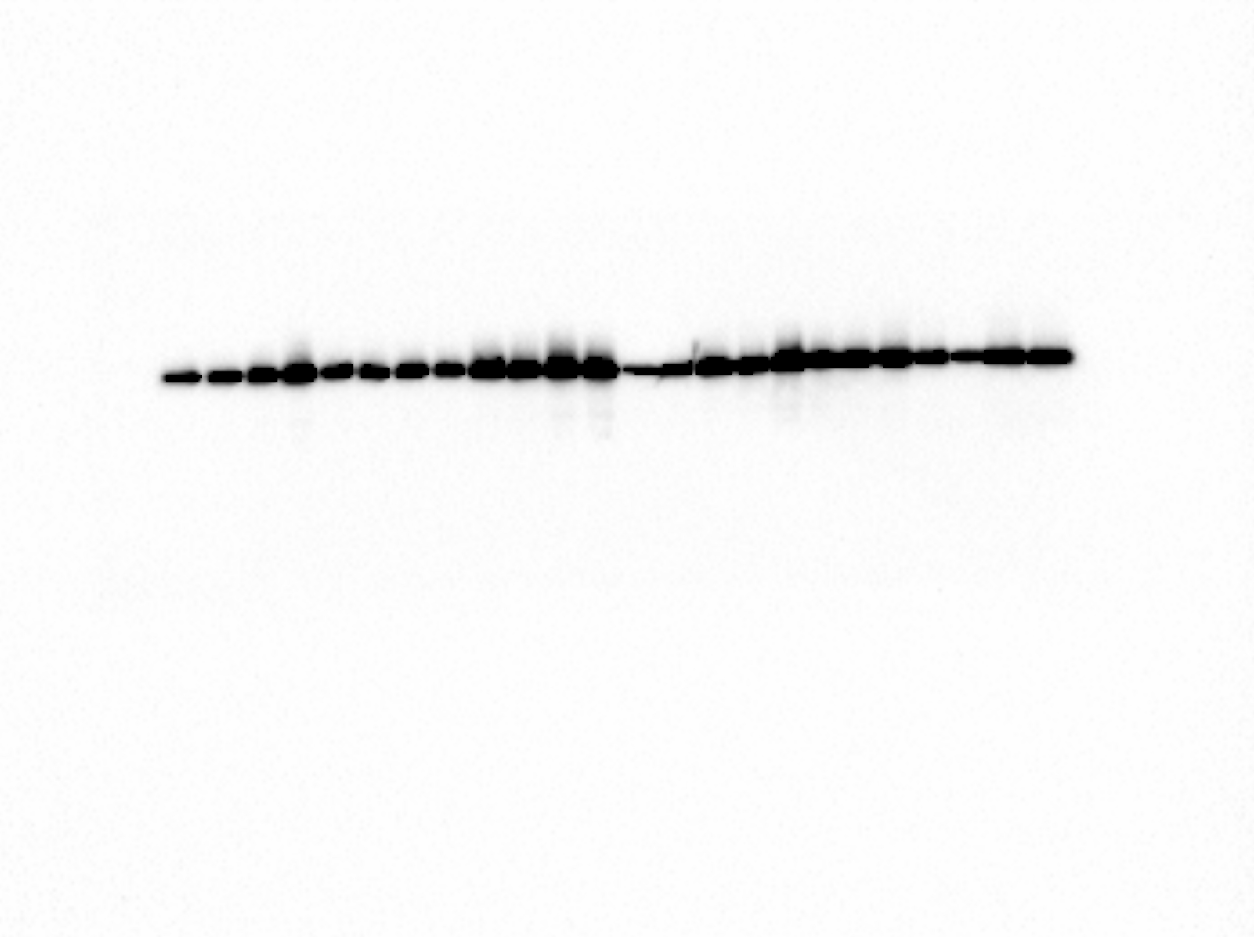


pS6 (shown above at 4 sec) shown here in the same series oversaturated at 15 sec exposure


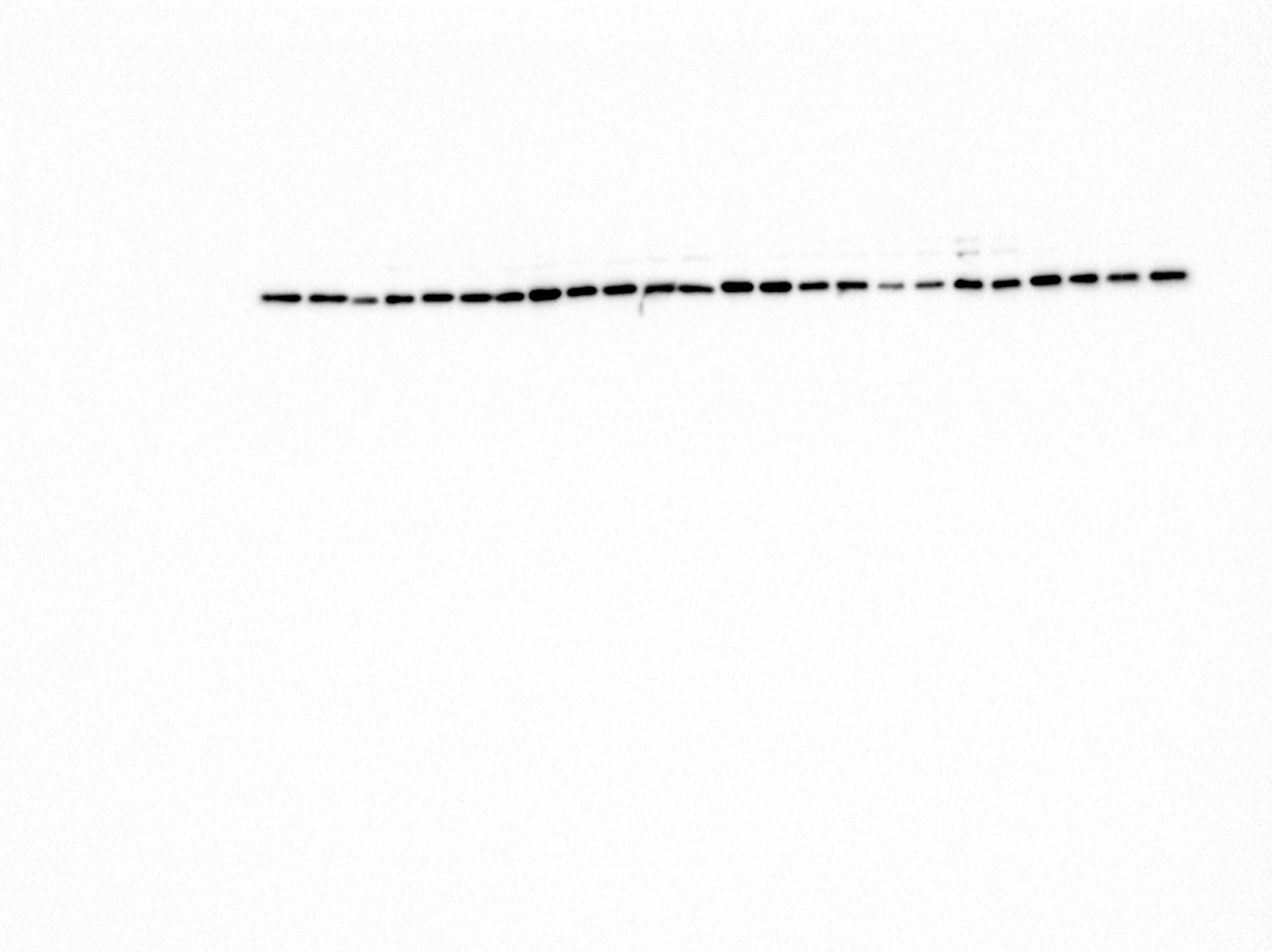


**Fig 2E S6**


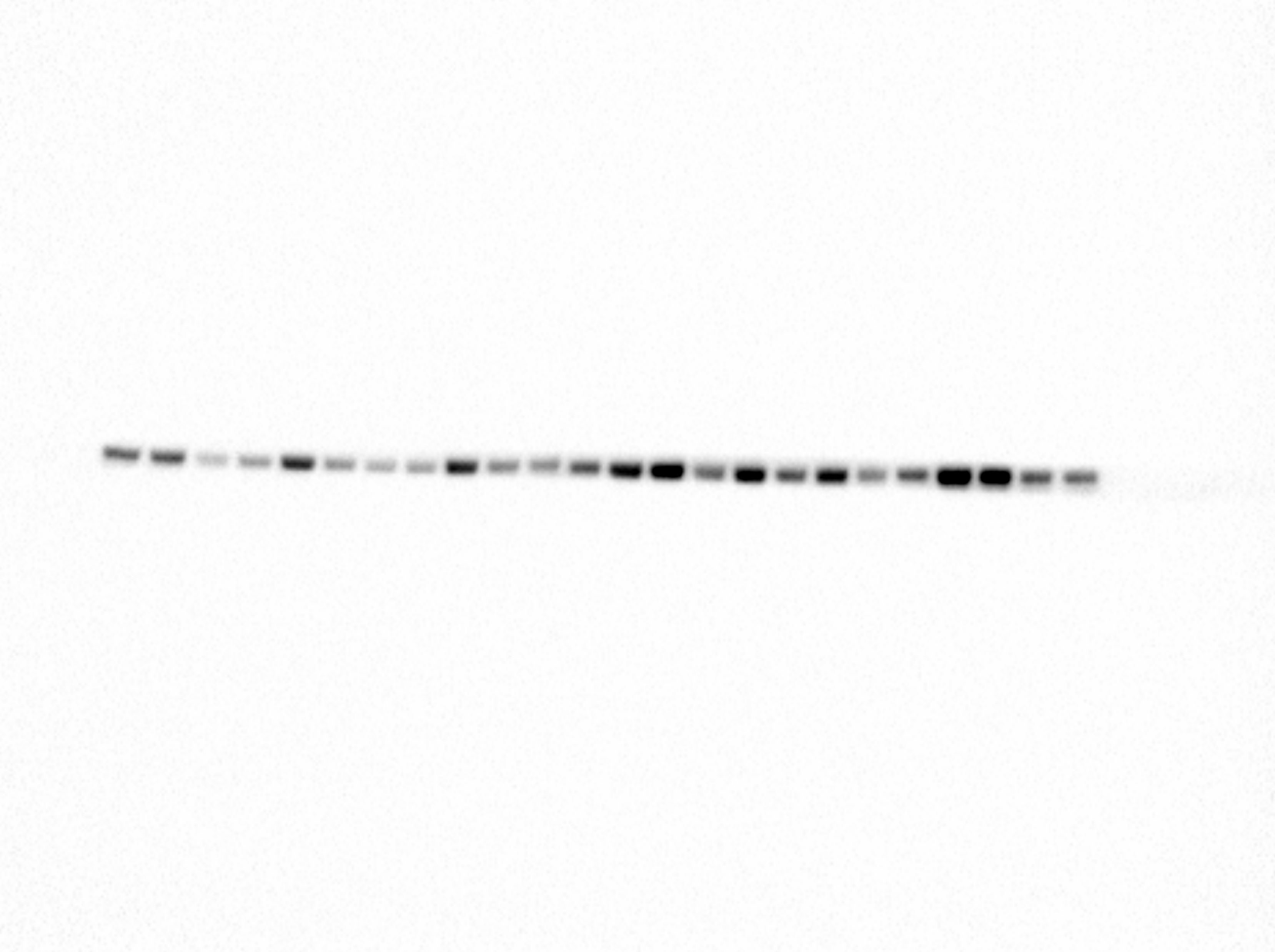


**IGF1R**

**Fig 4B**

**Fig 4D**

**IGF1R**


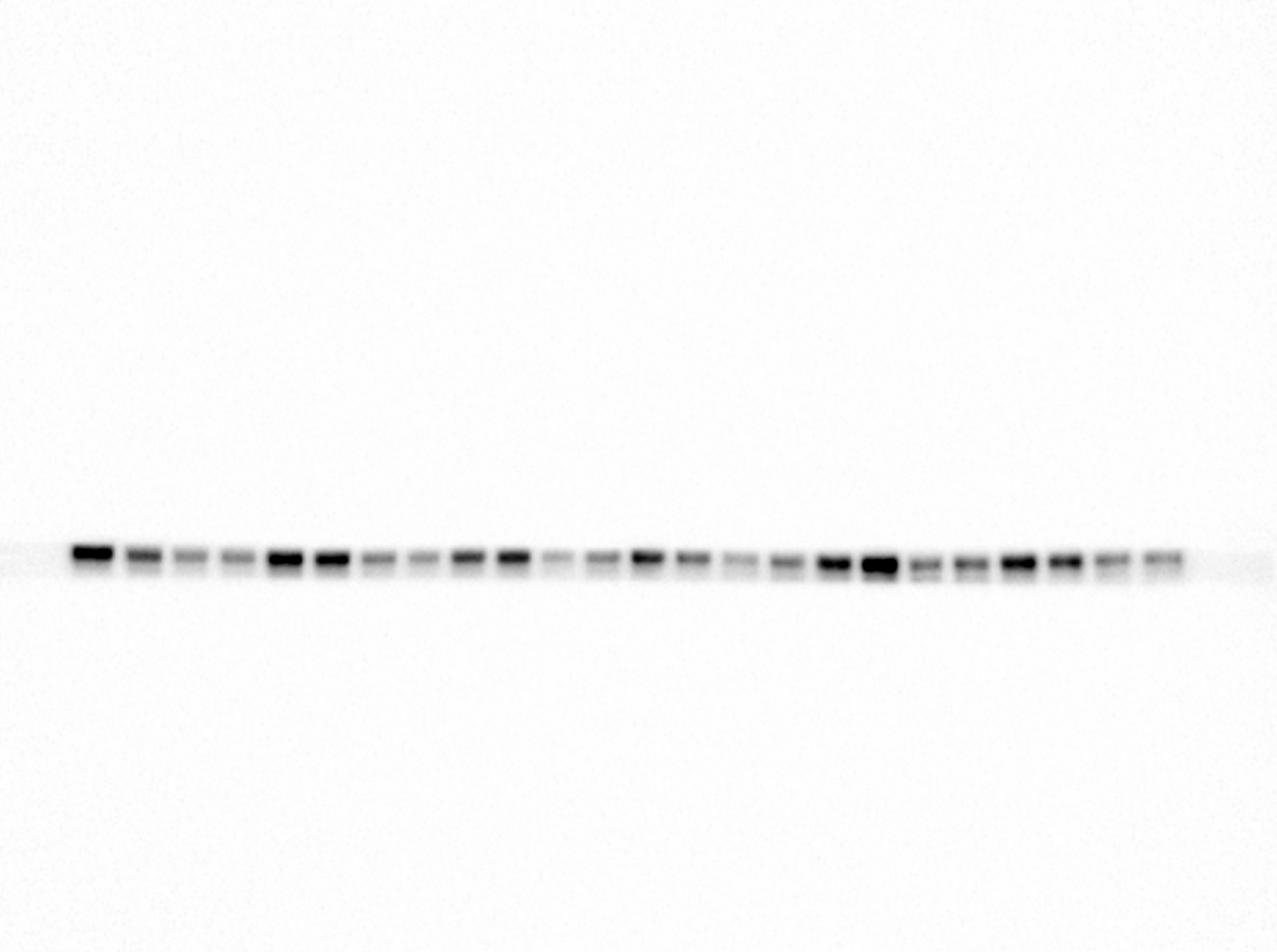


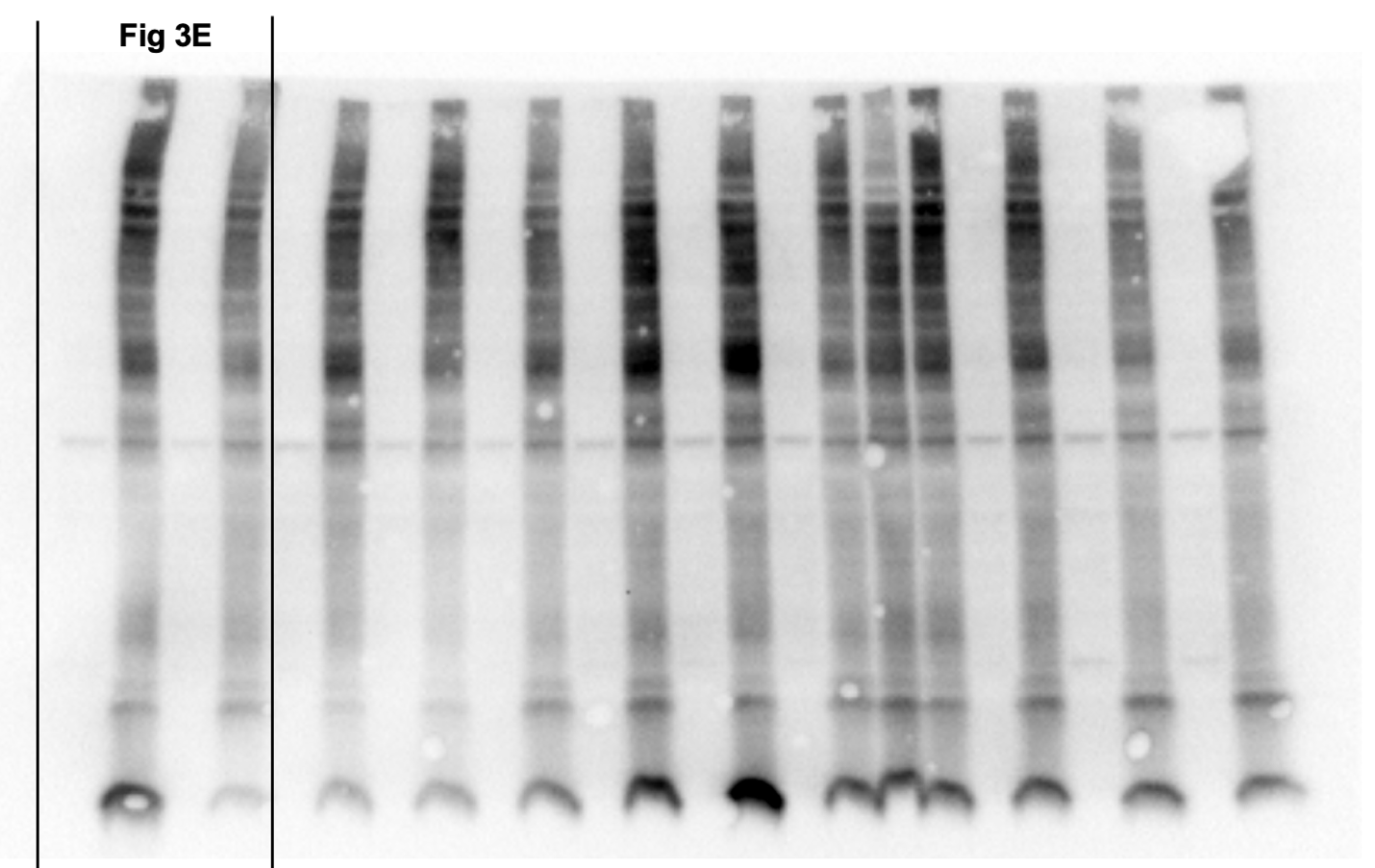

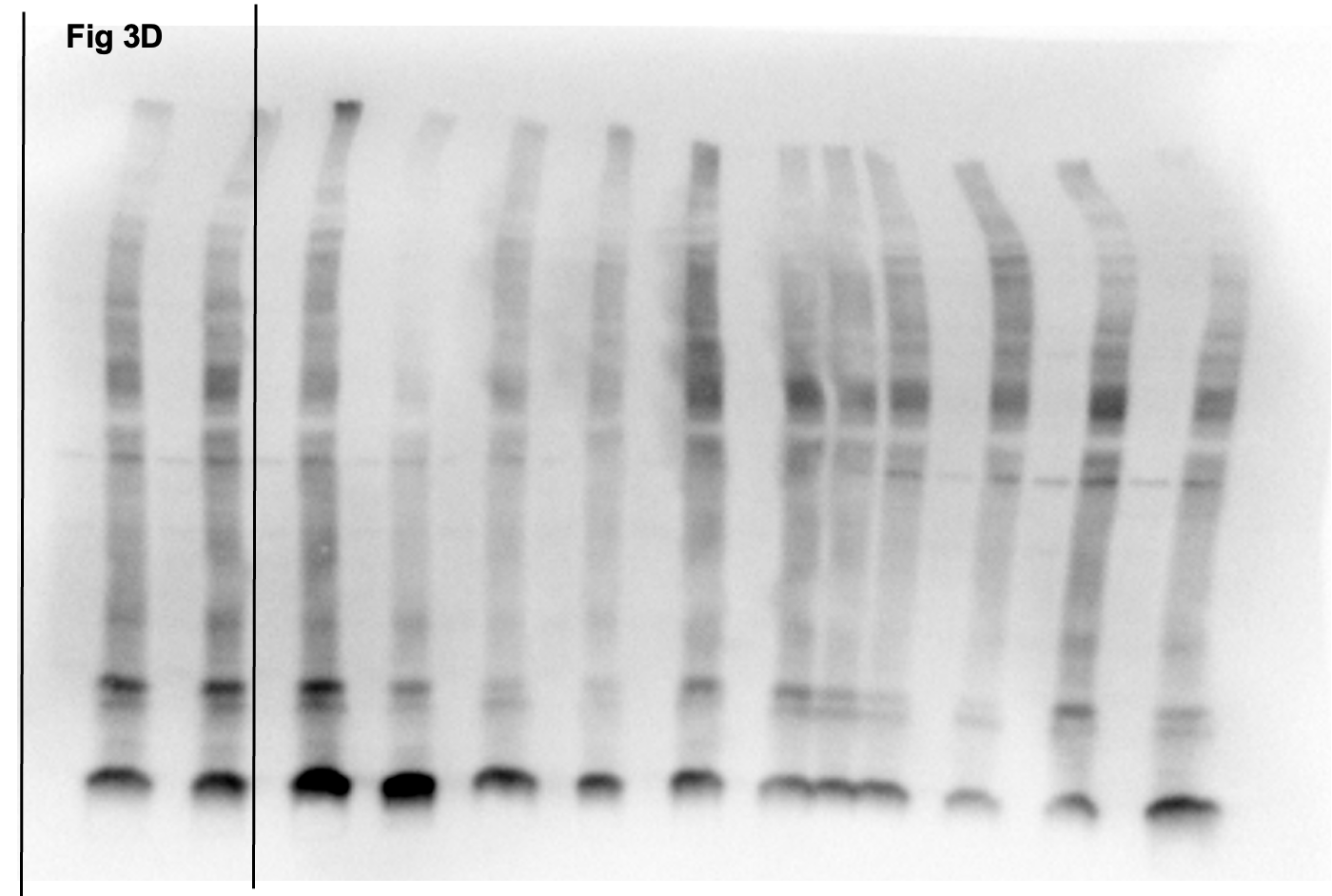


**Fig 5D**

**Fig 5C**


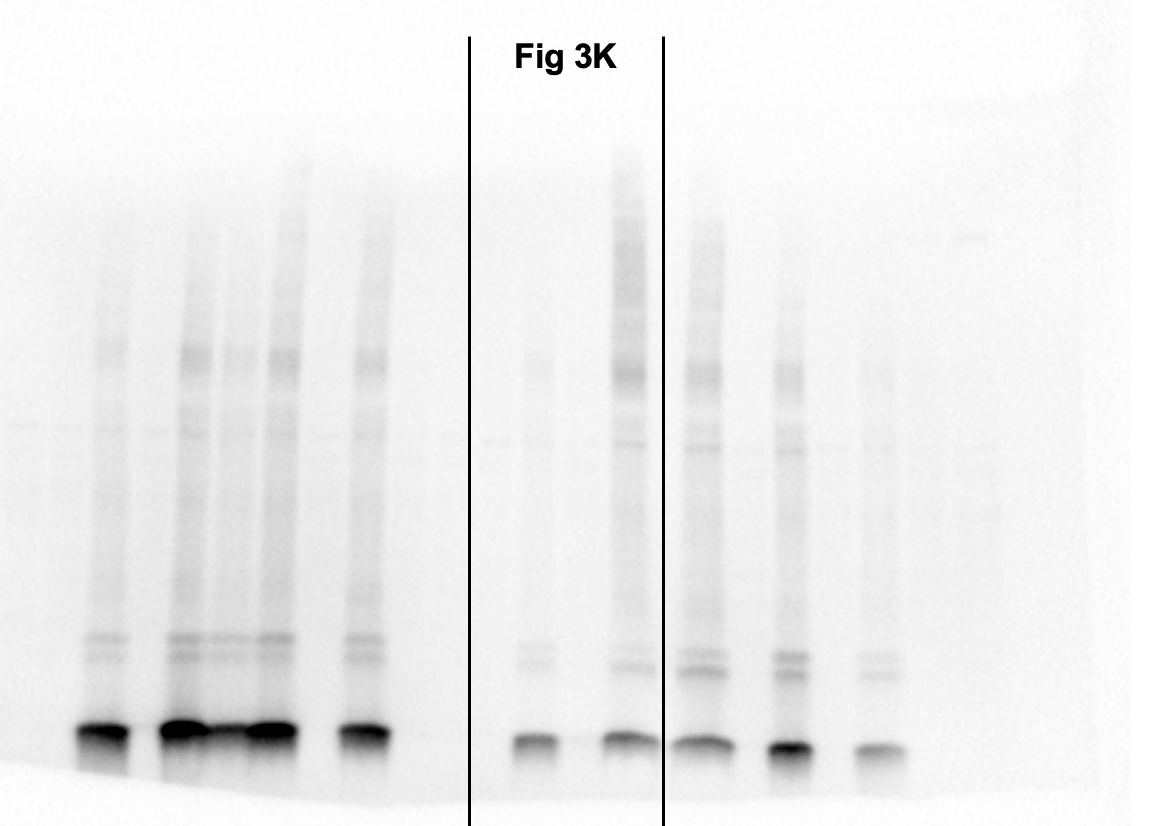


**Fig 5I**


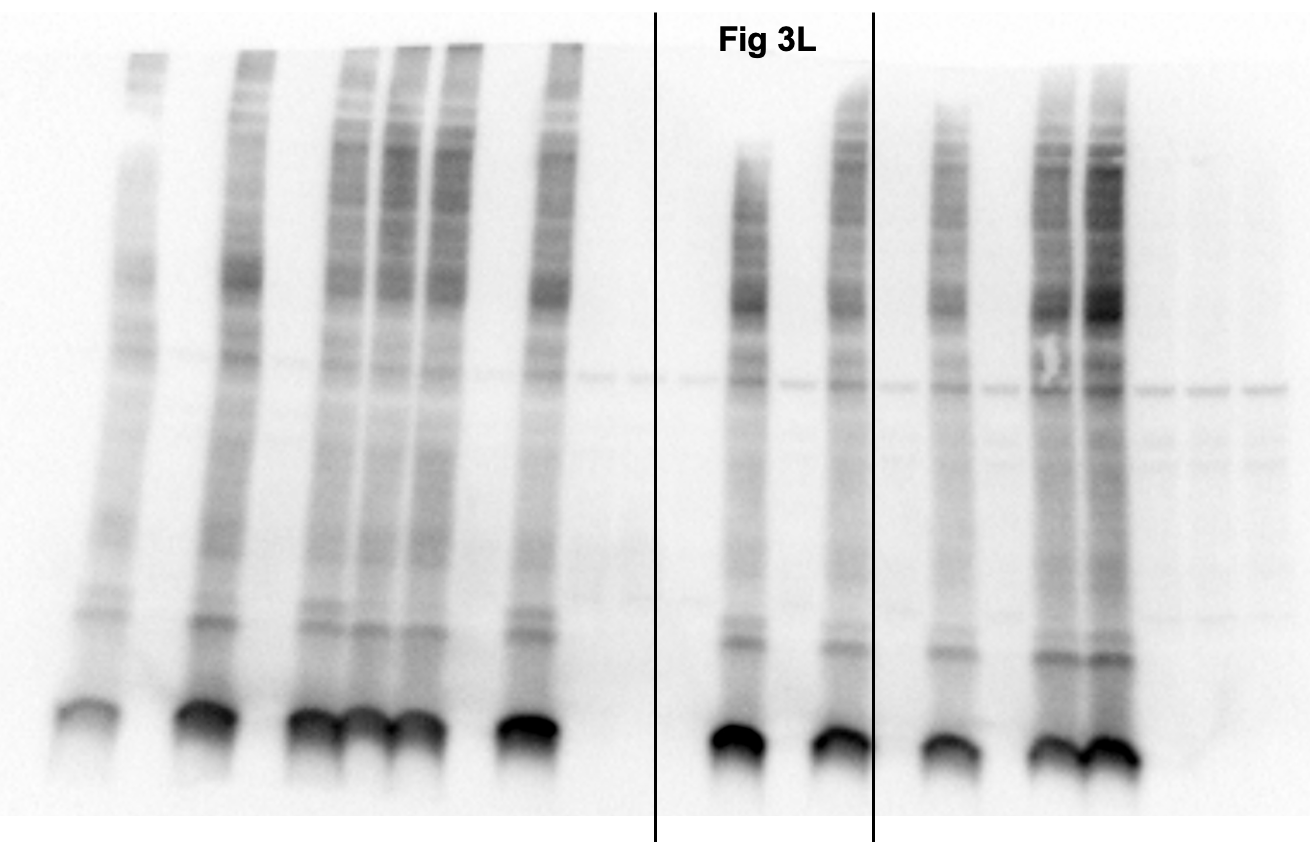


**Fig 5J**


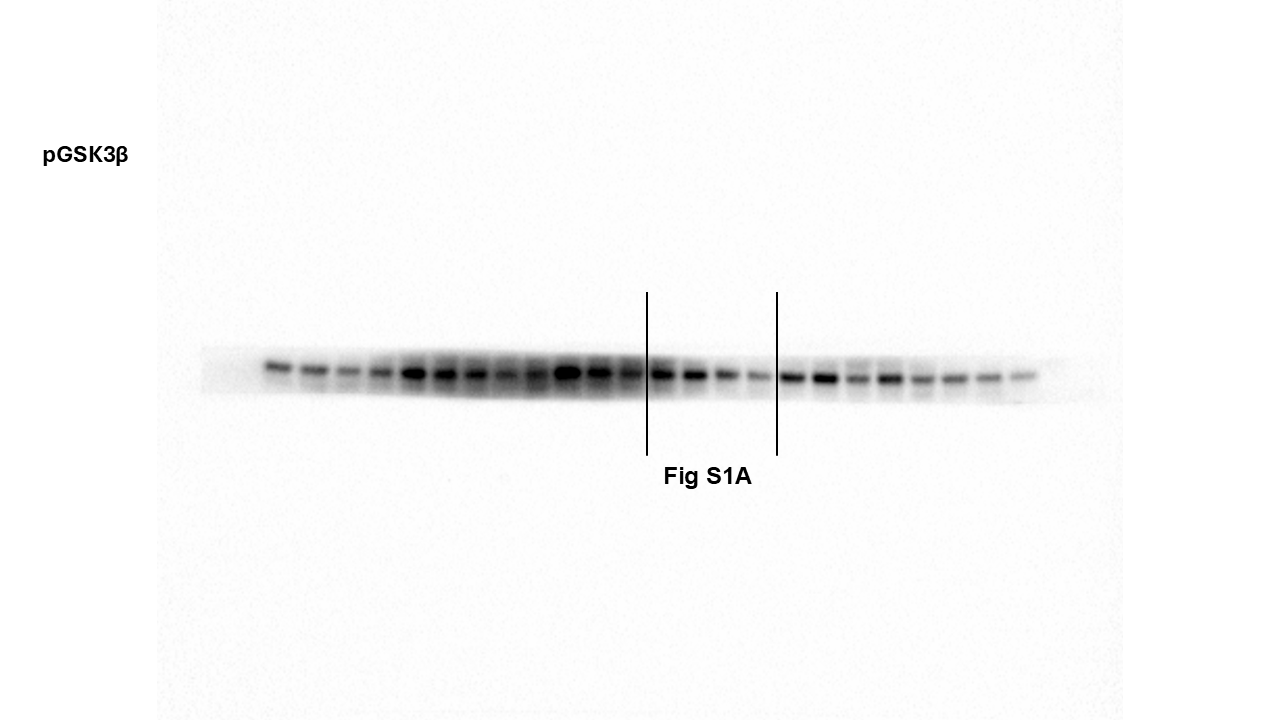
Figure S1 Blots:

**Fig S1A**


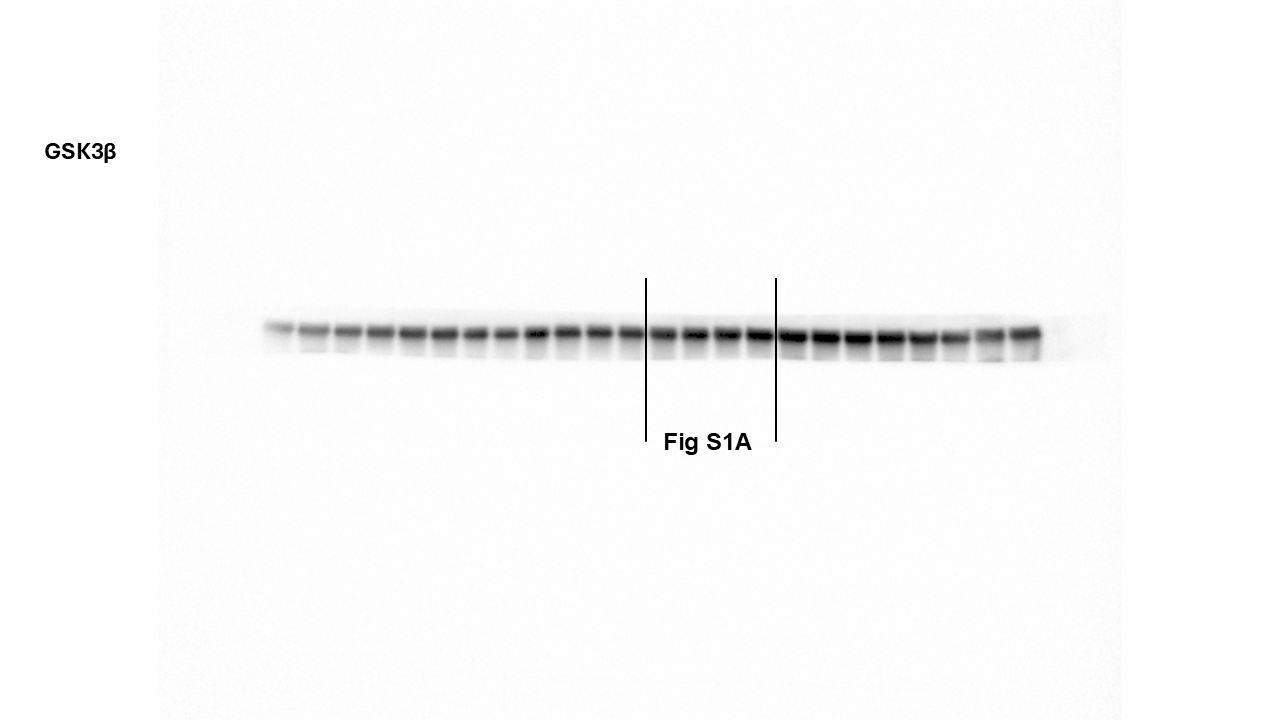


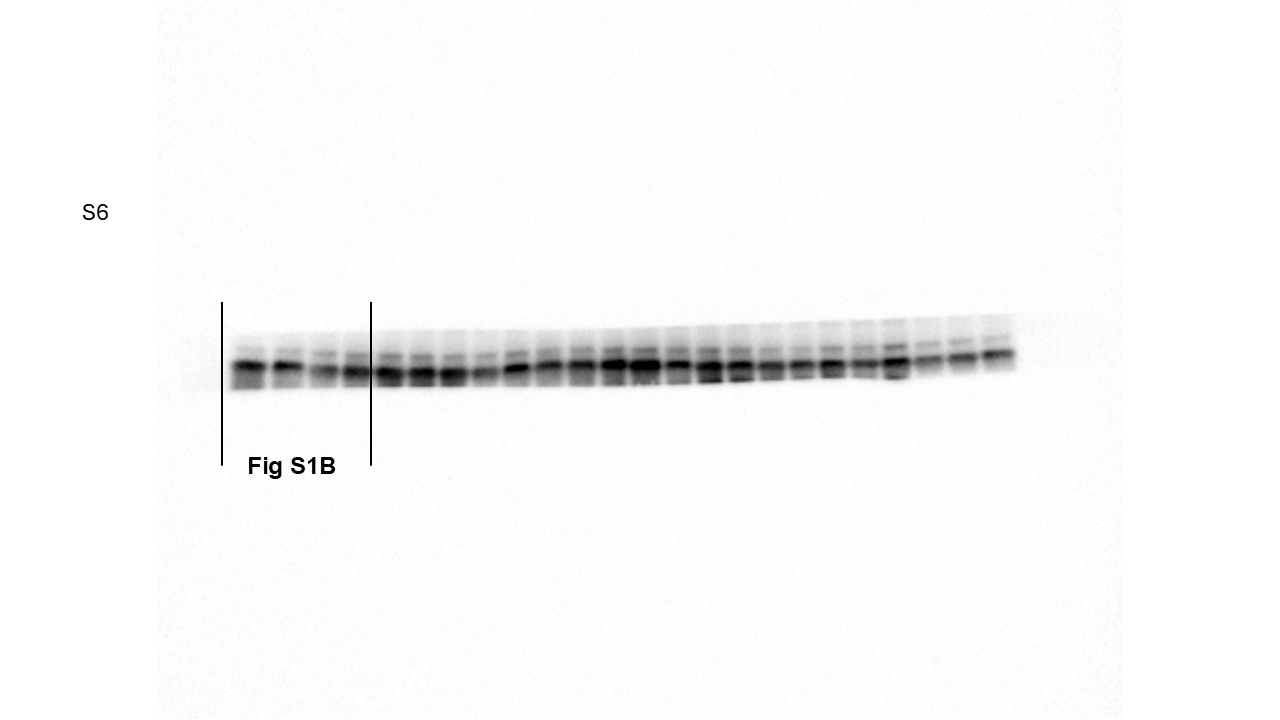


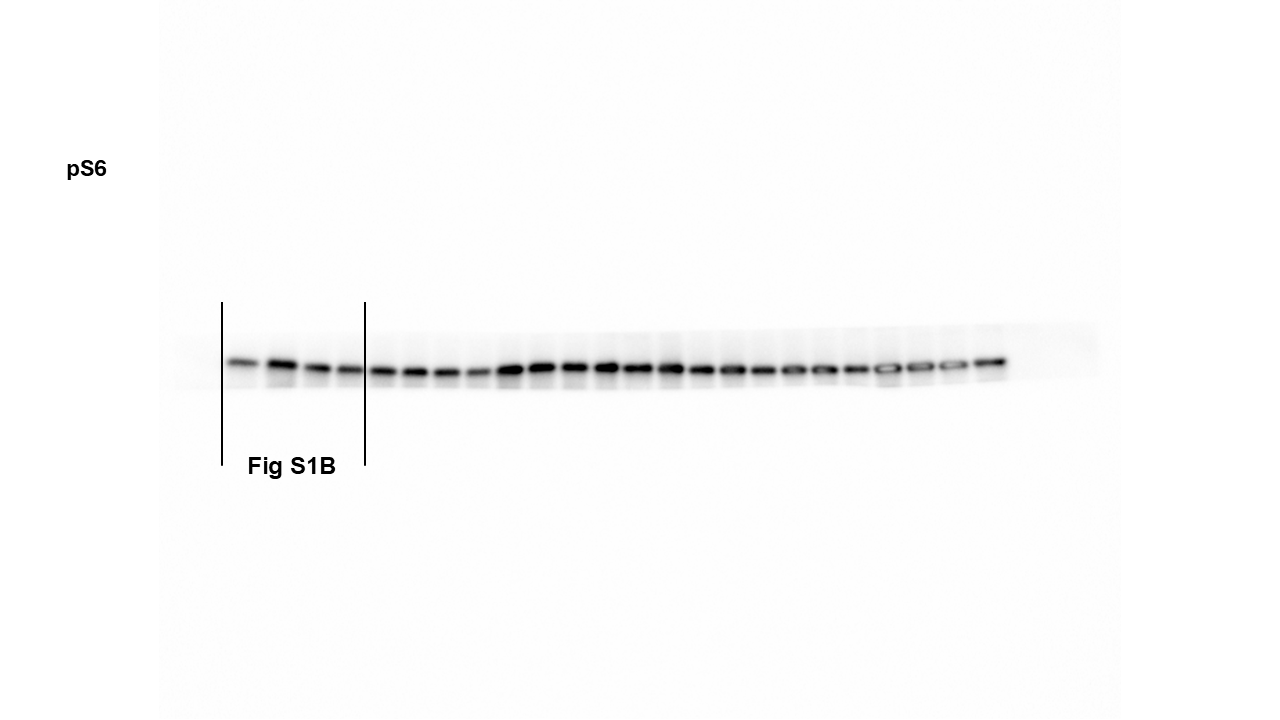


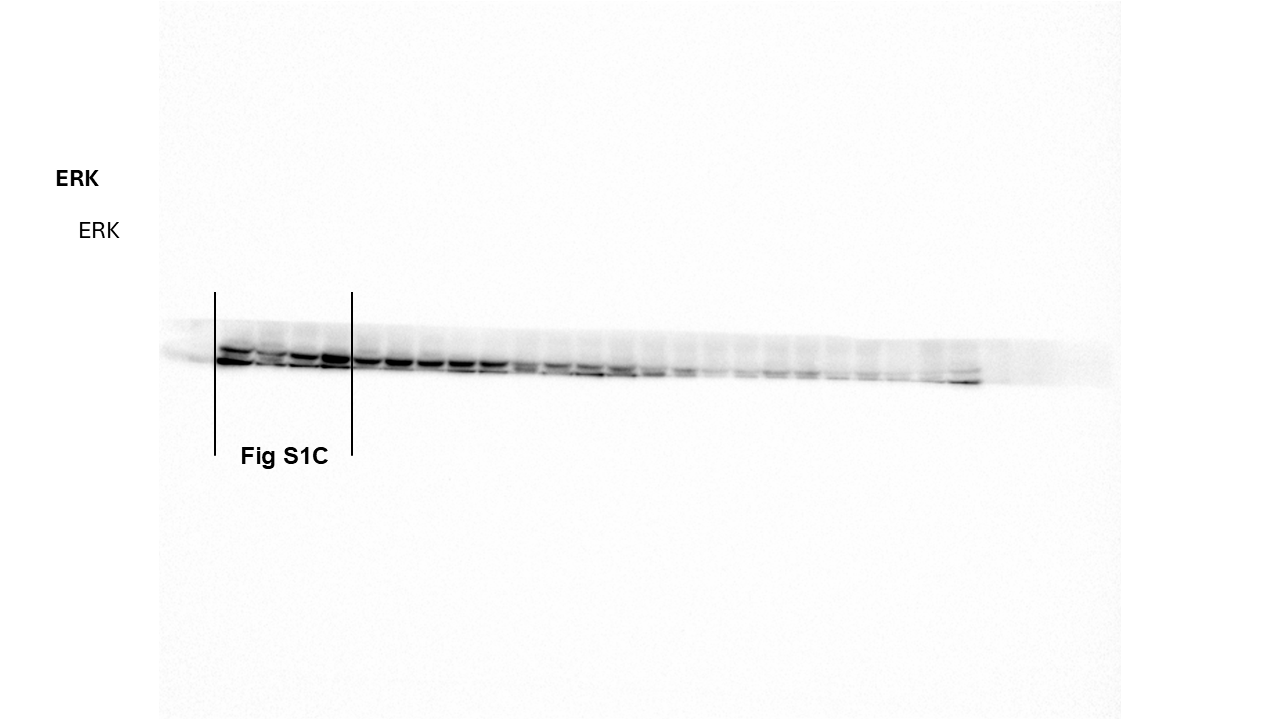

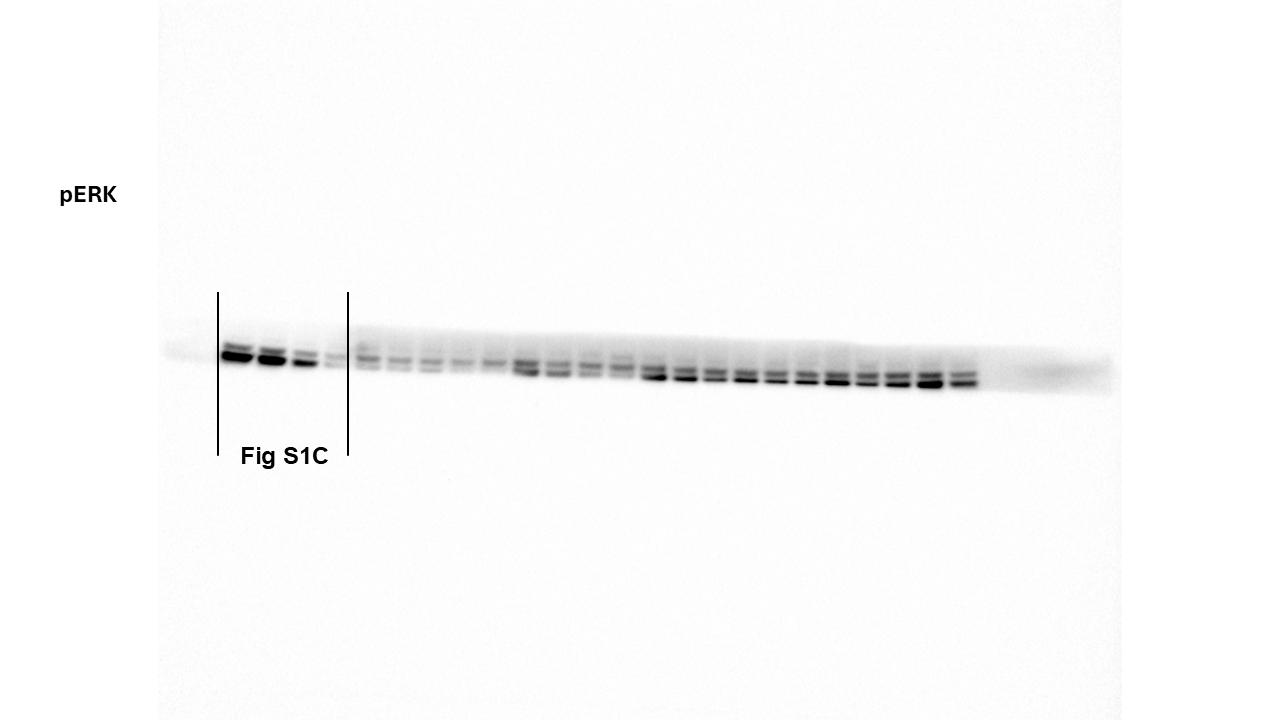


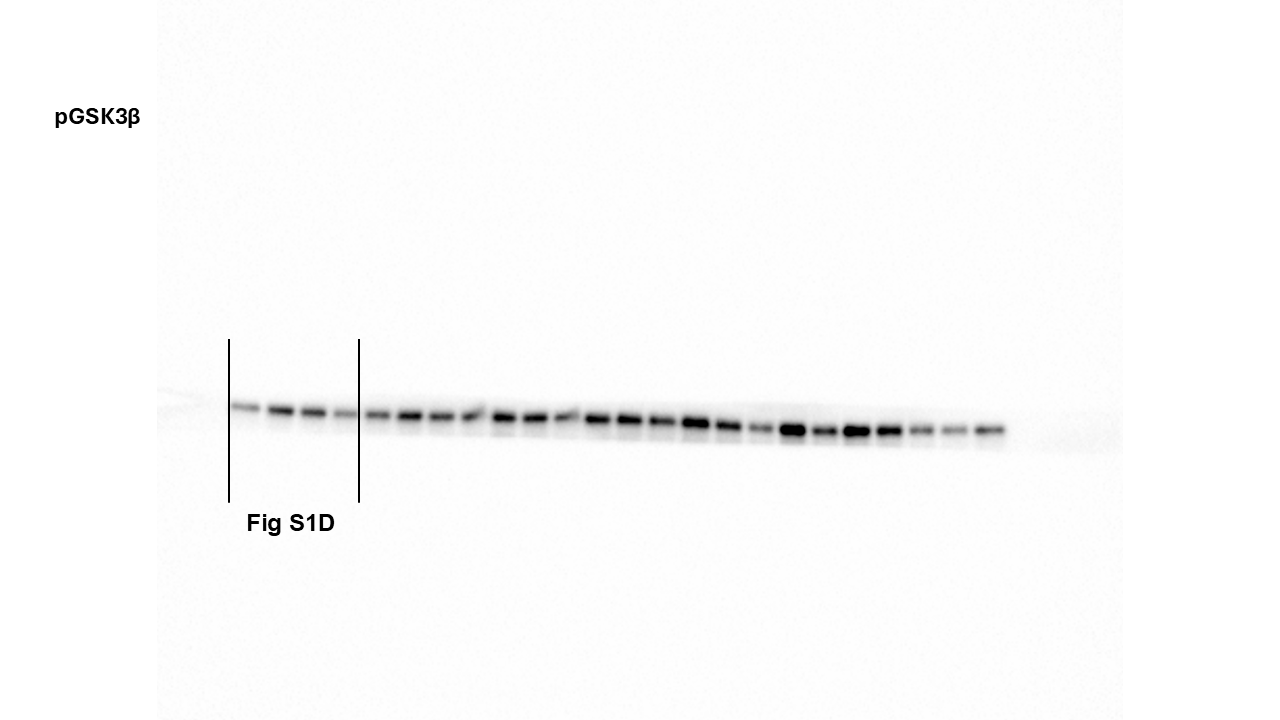

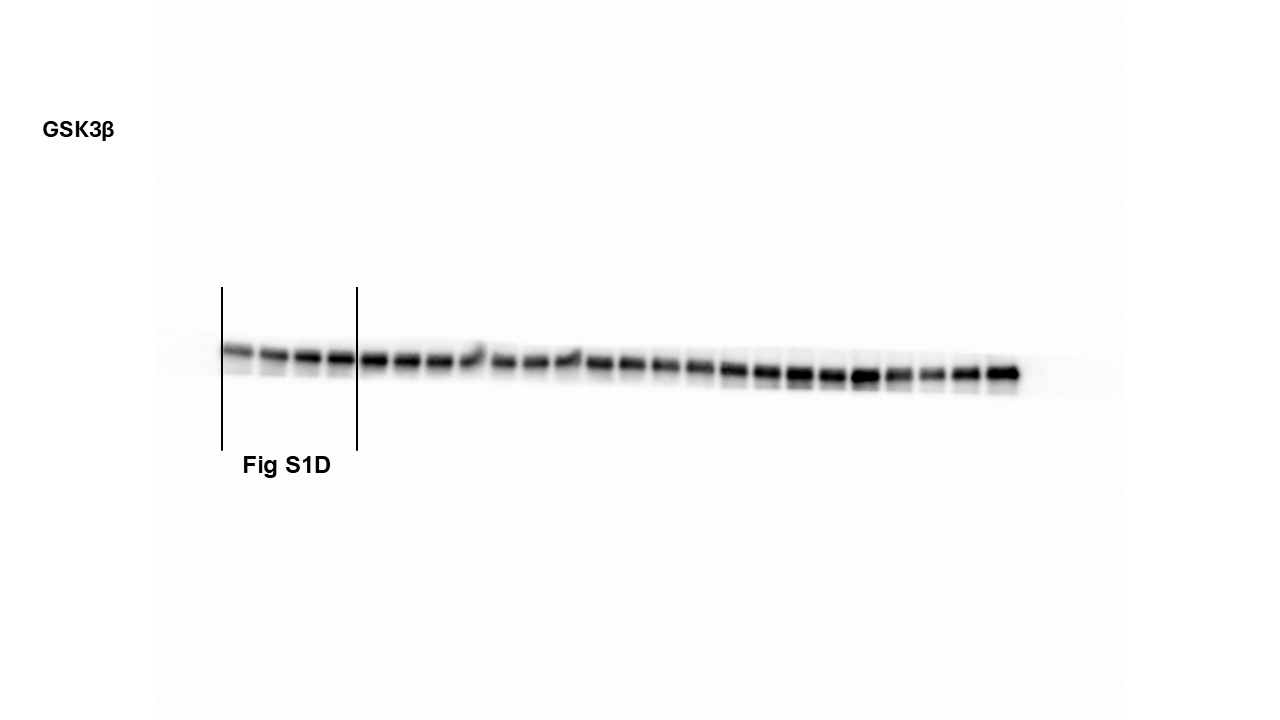

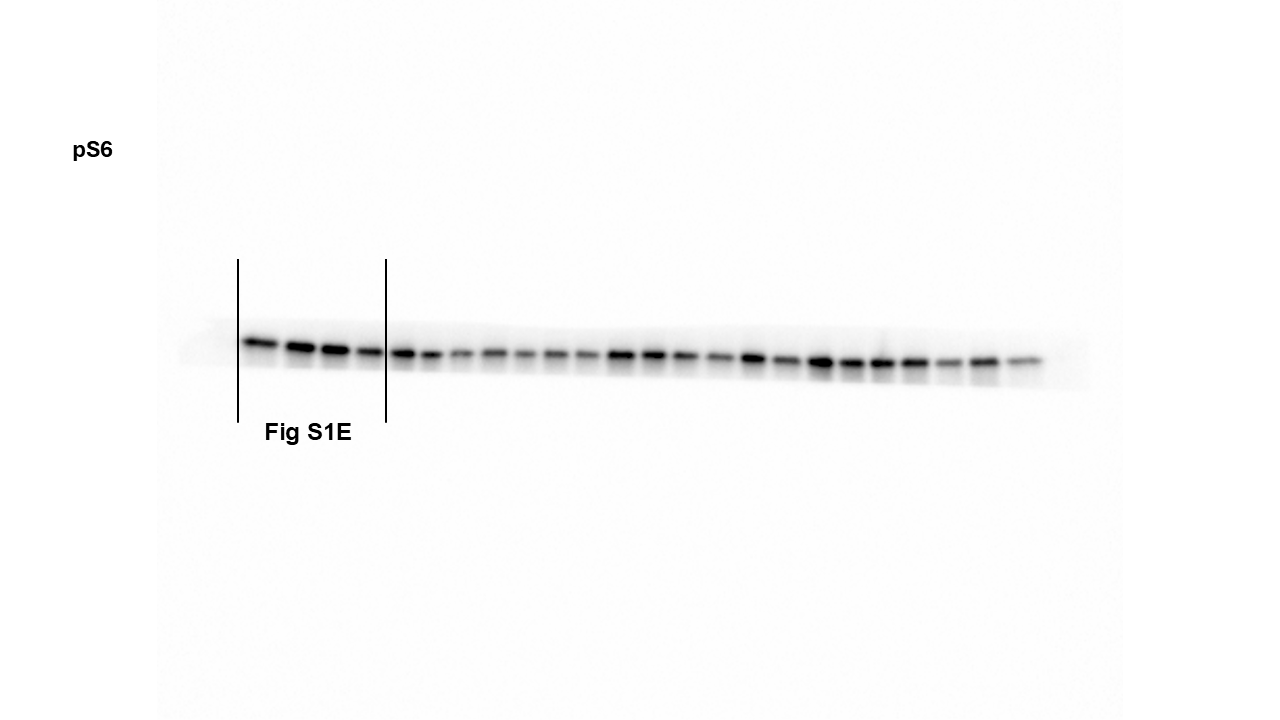

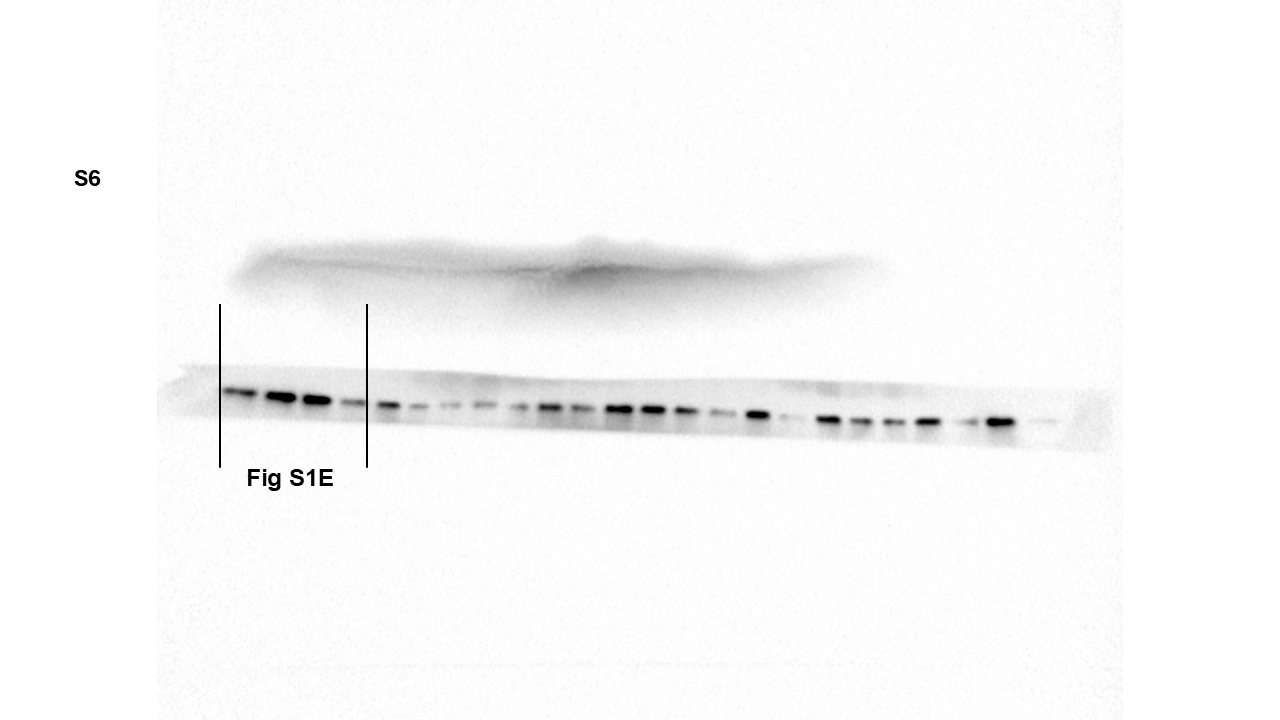

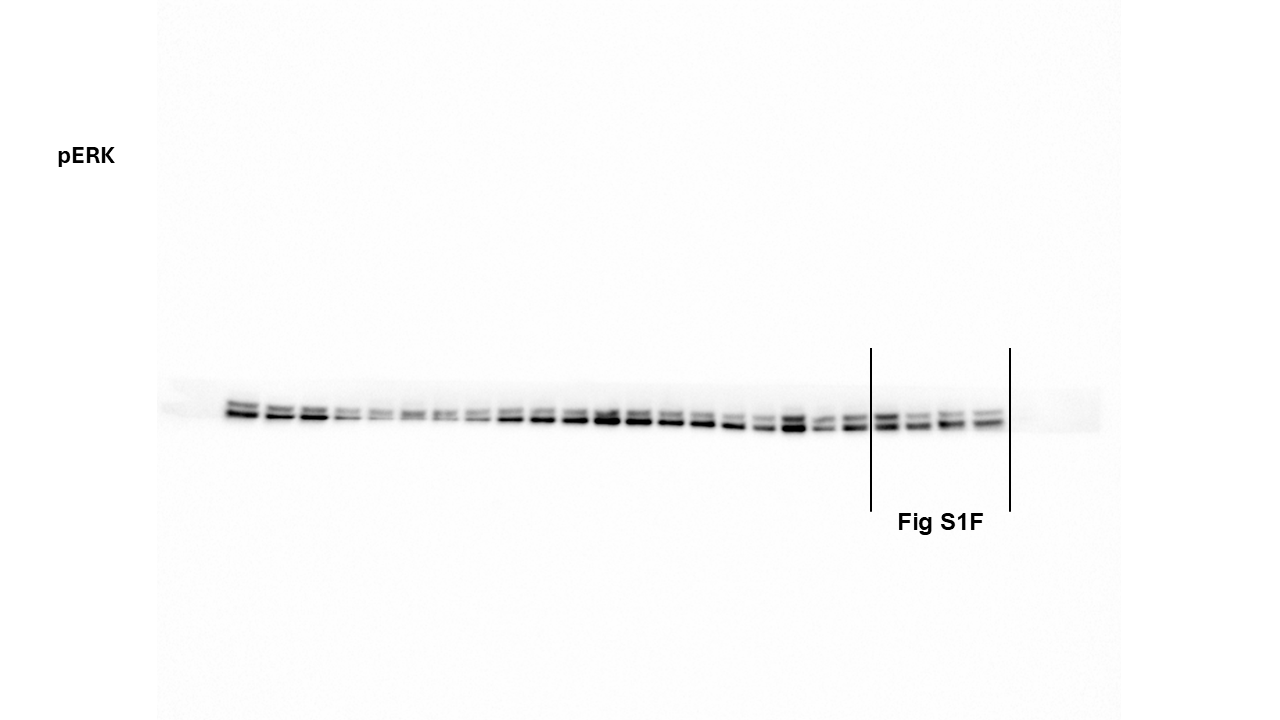

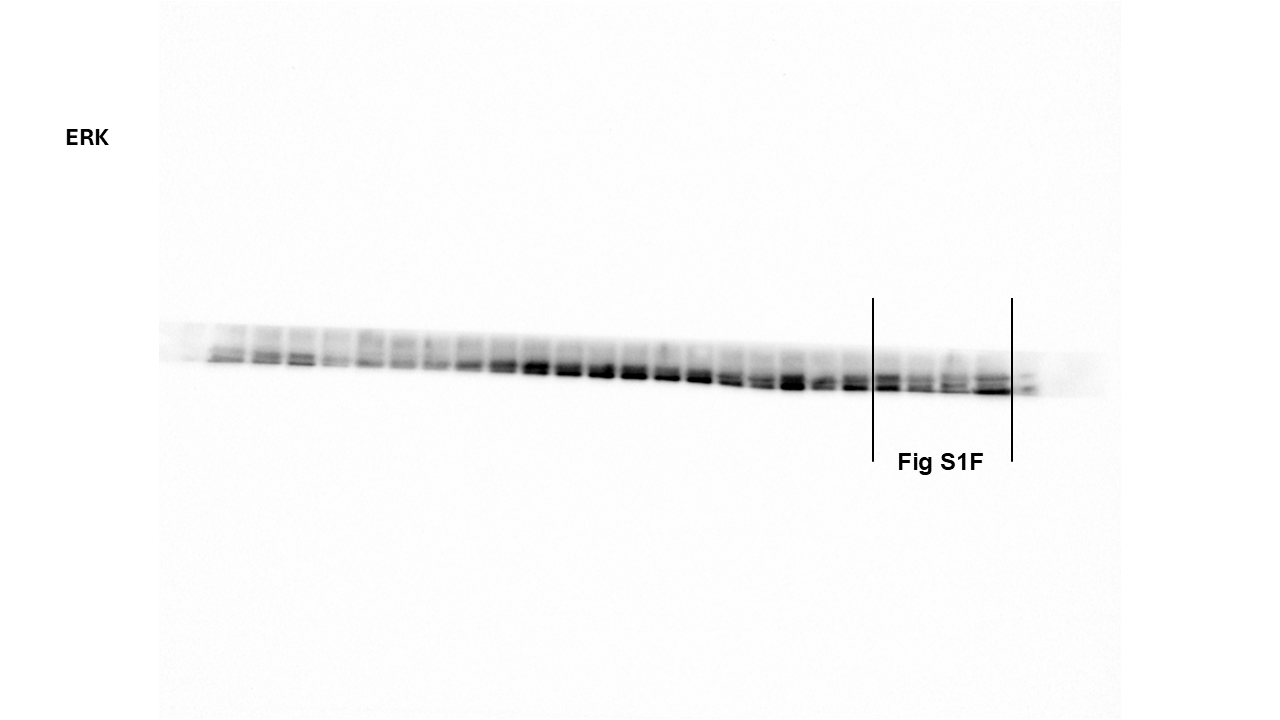


Figure S2 Blots:


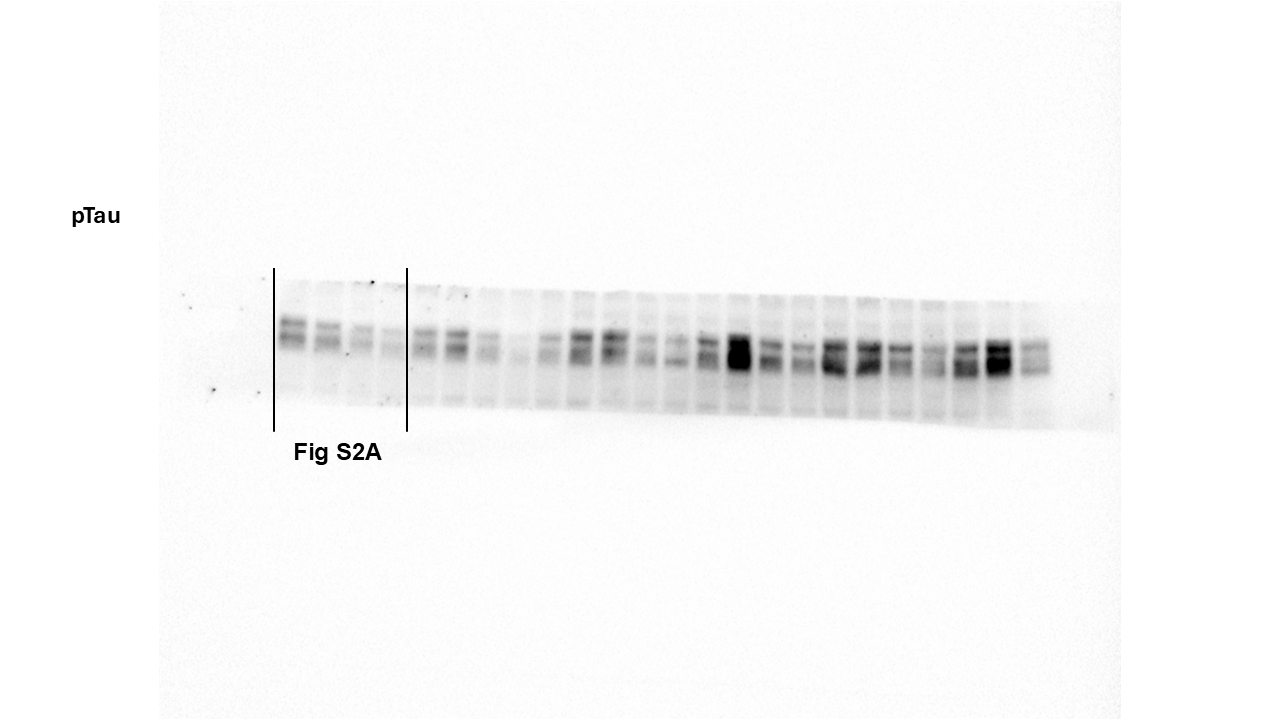

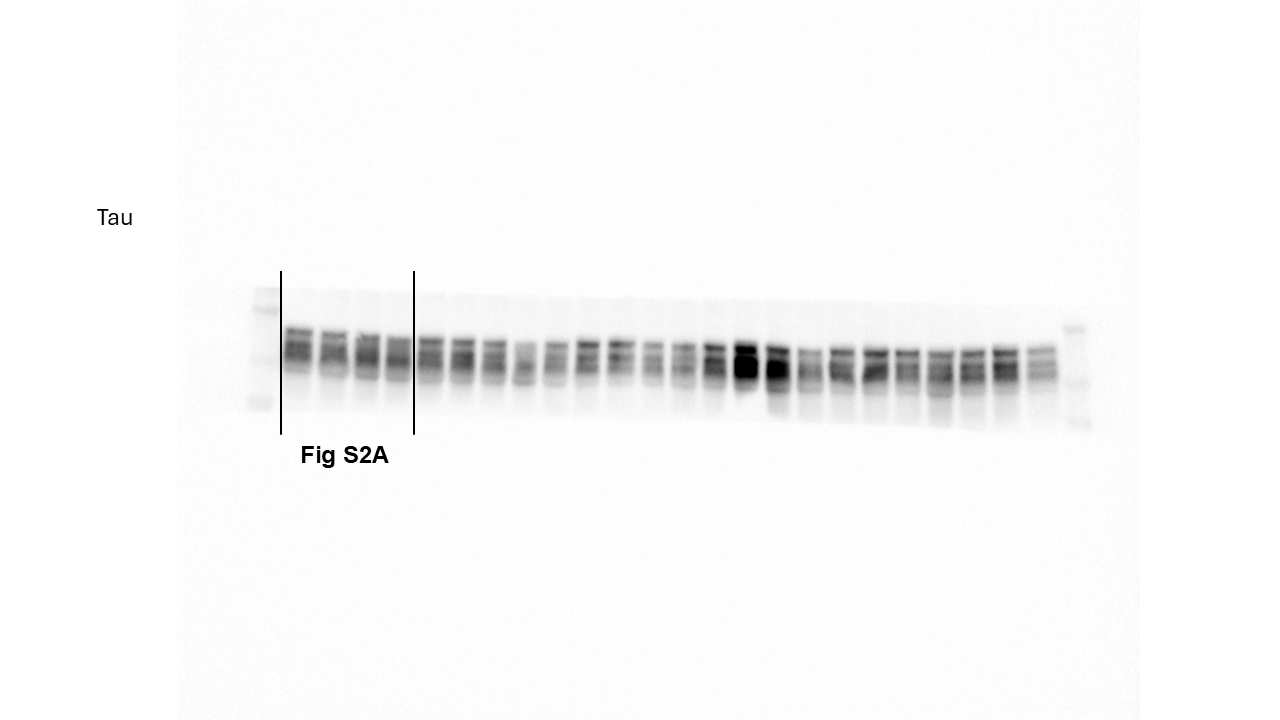


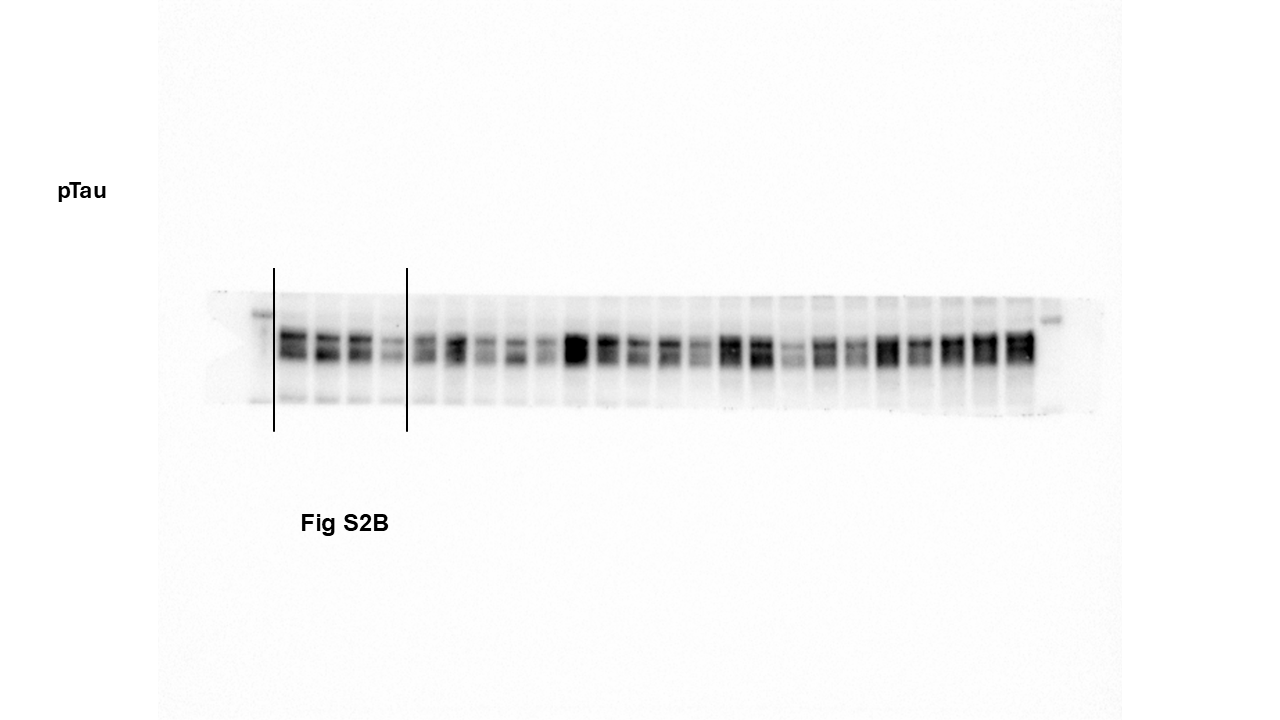

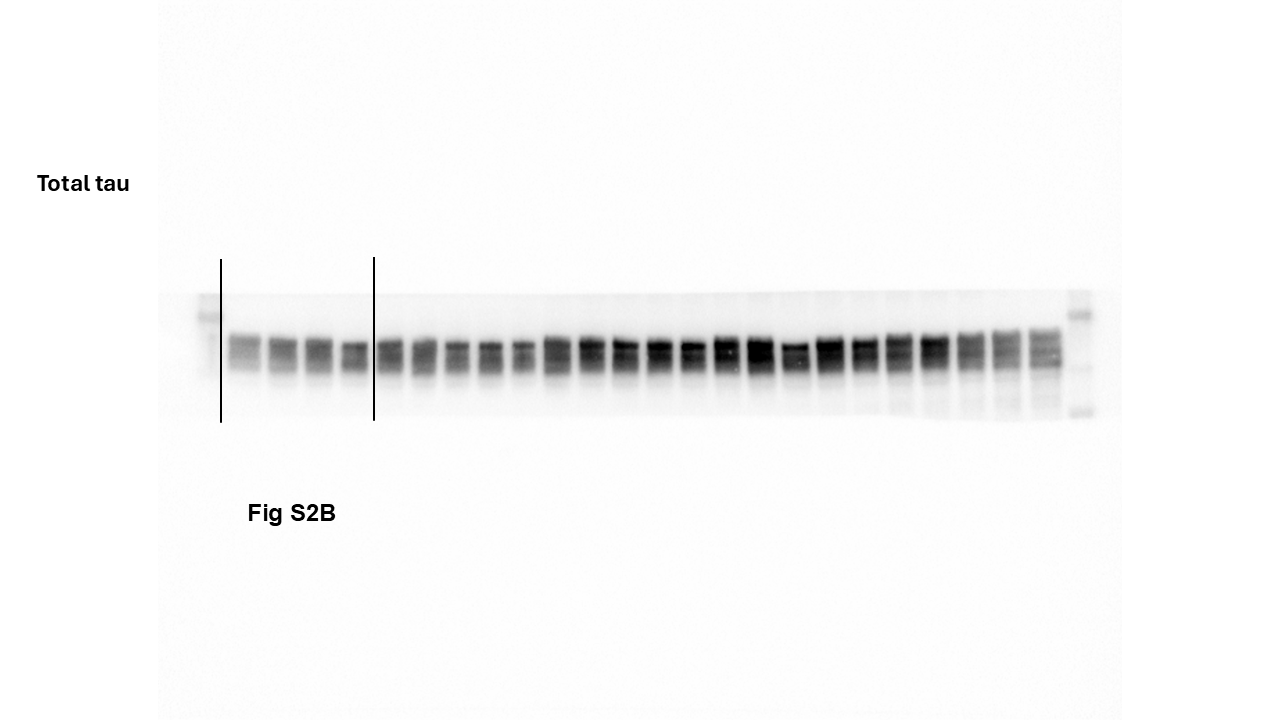


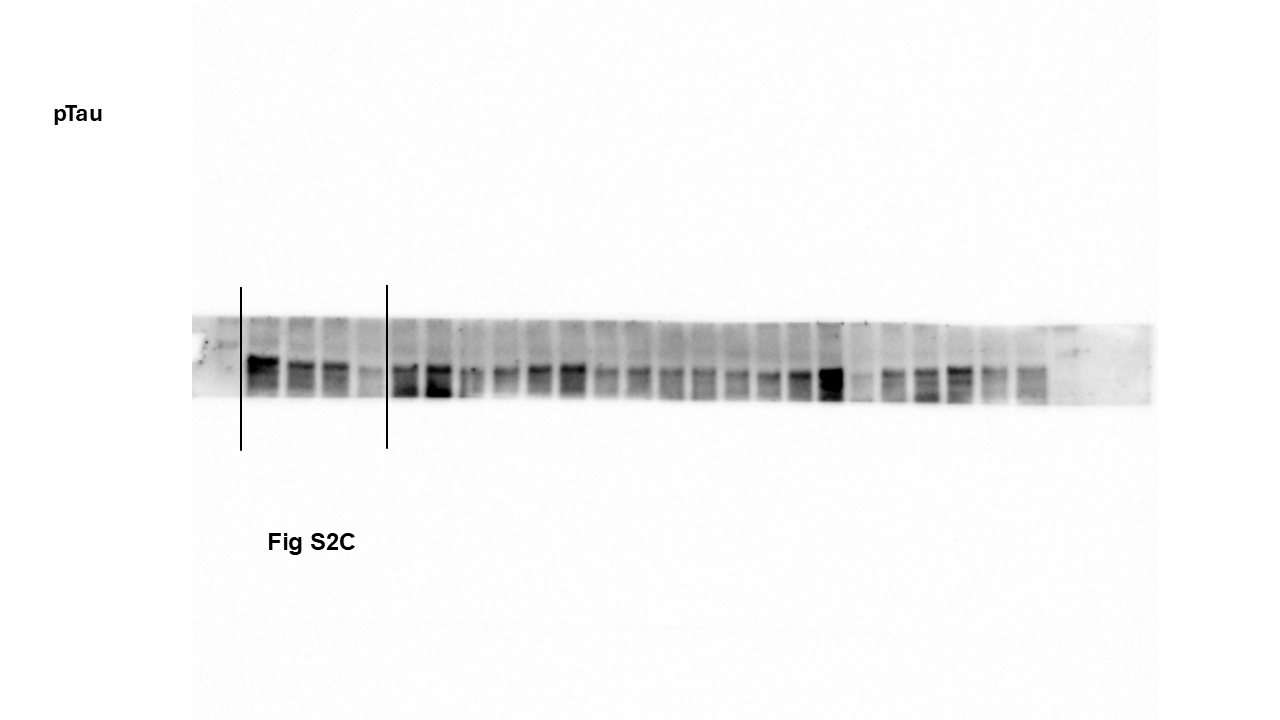

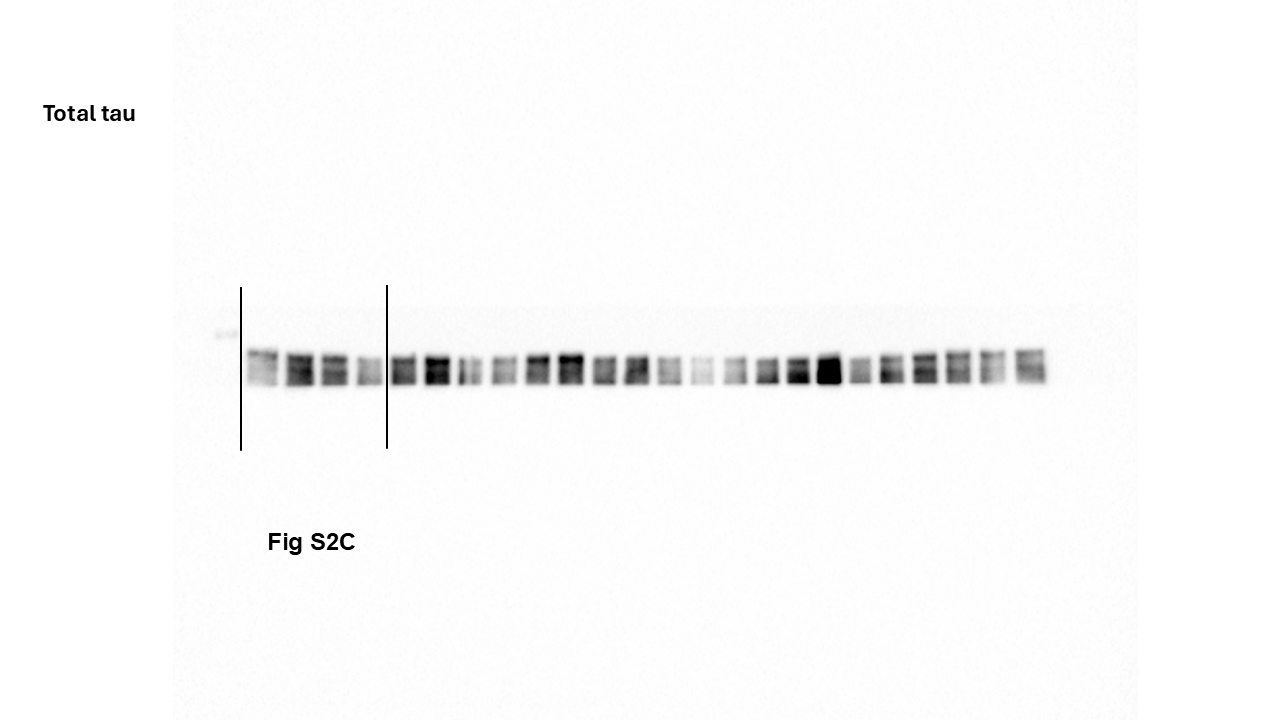


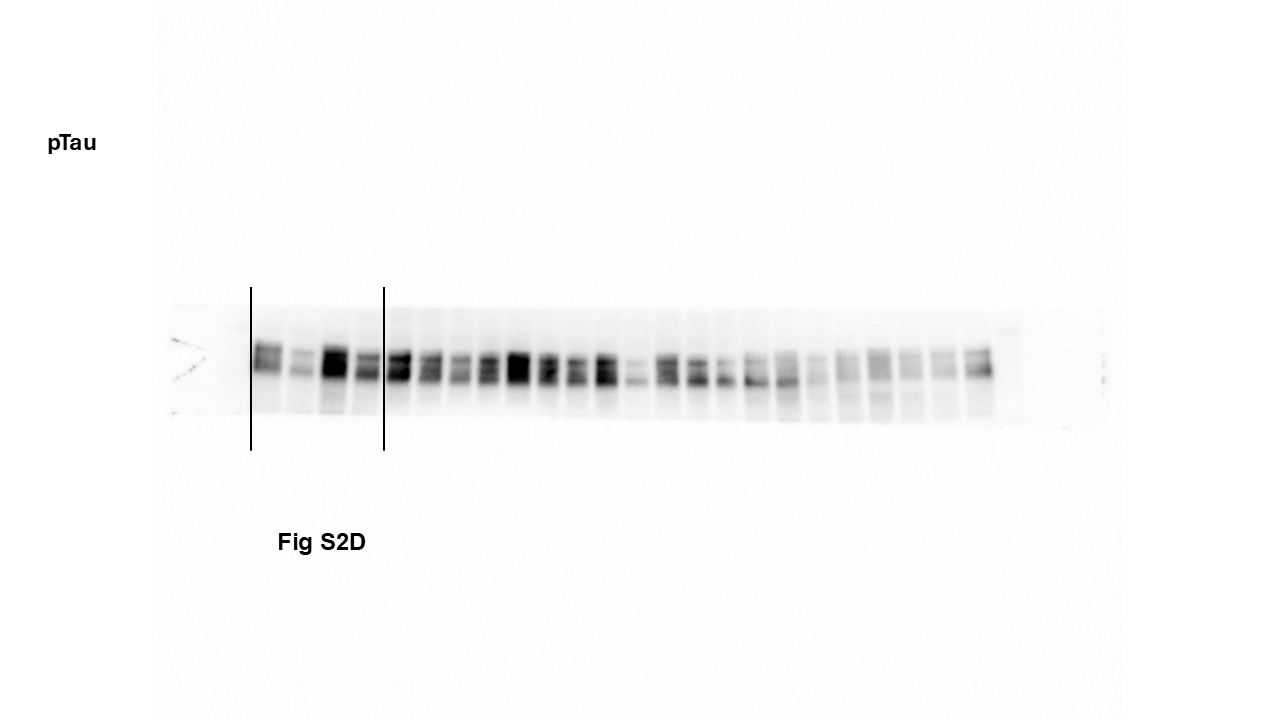

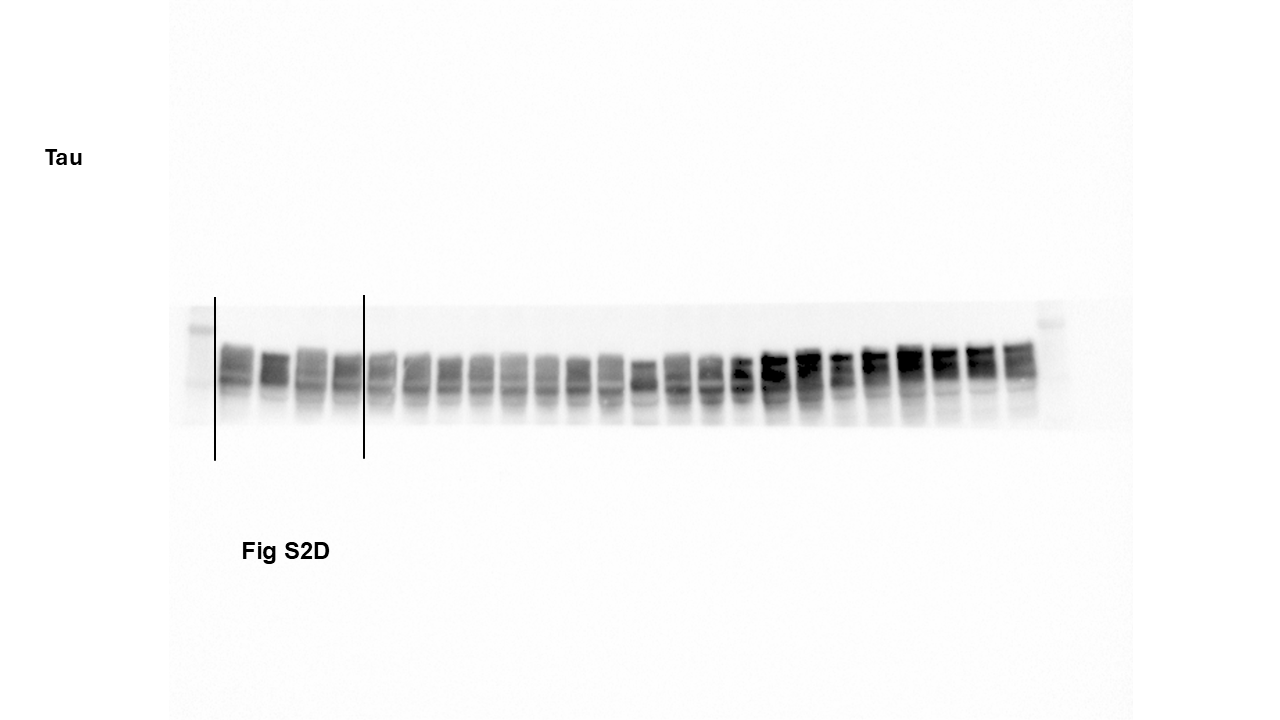


Figure S3 Blots:


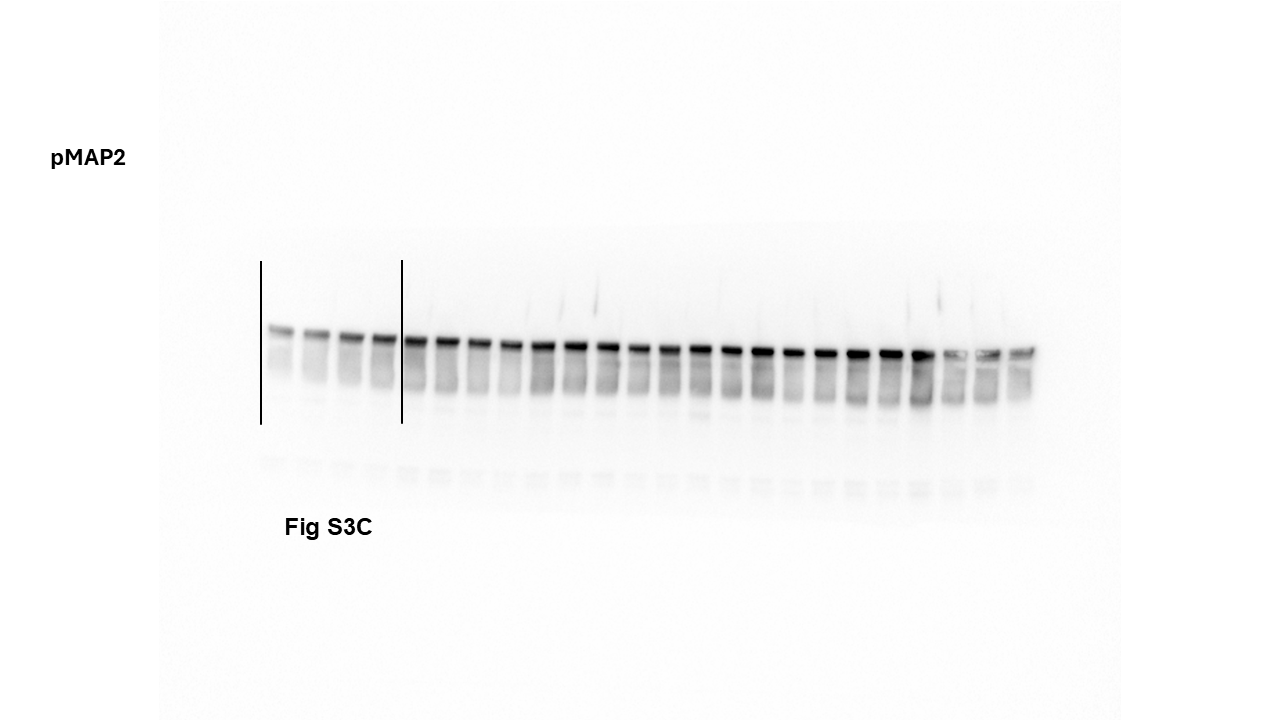

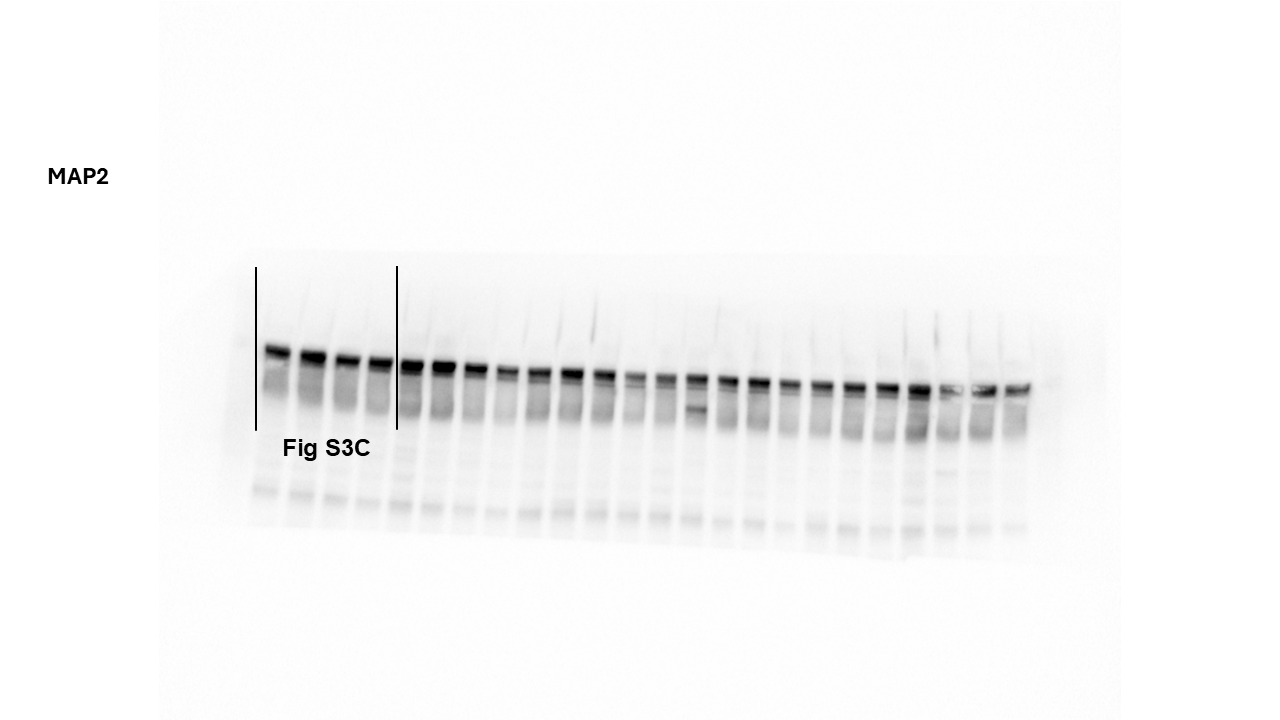


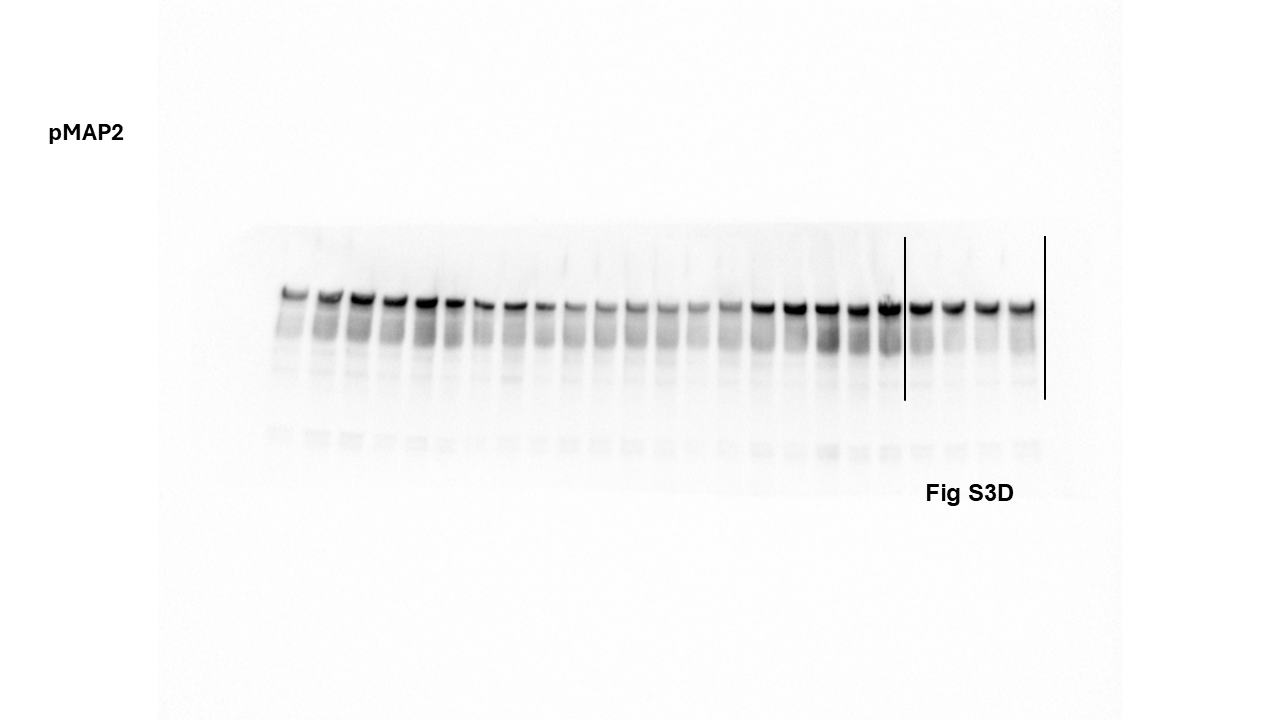

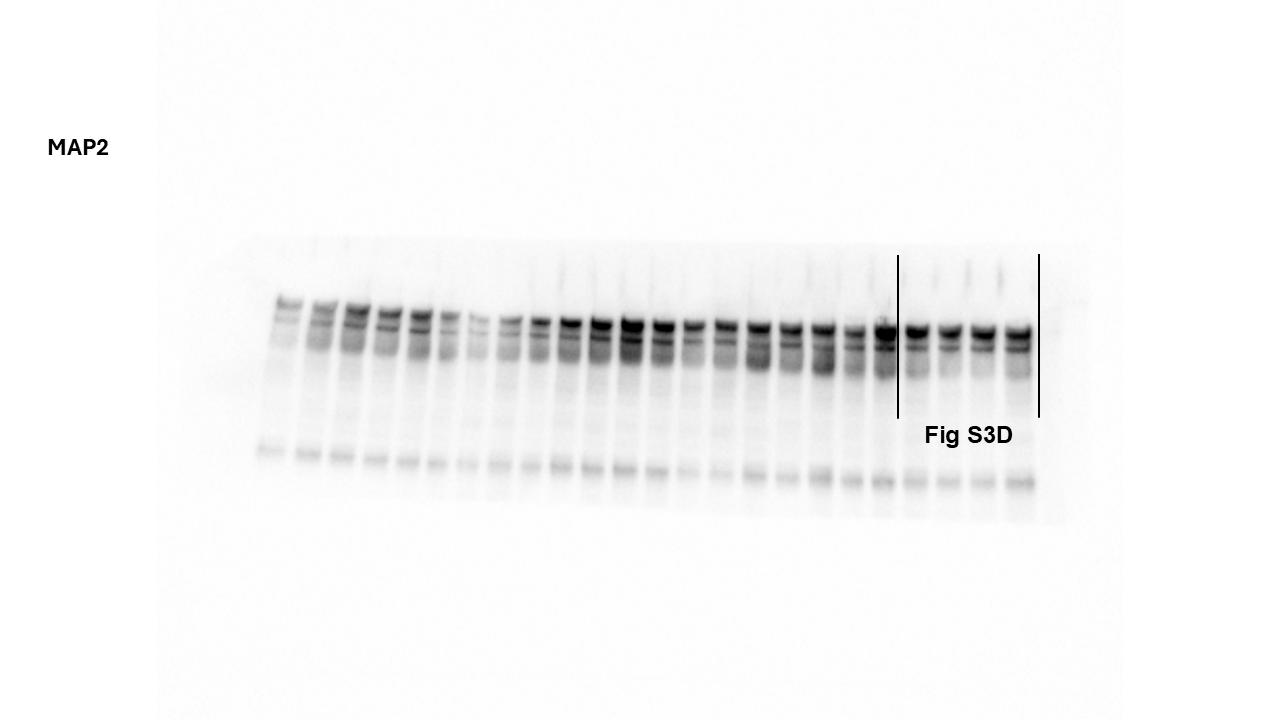

Supplement: Supplementary file 1 — Supplementary Information. [file 41598_2025_31601_MOESM1_ESM.docx]
